# An Intranasal Proteosome-Adjuvanted Trivalent Influenza Vaccine Is Safe, Immunogenic & Efficacious in the Human Viral Influenza Challenge Model. Serum IgG & Mucosal IgA Are Important Correlates of Protection against Illness Associated with Infection

**DOI:** 10.1371/journal.pone.0163089

**Published:** 2016-12-22

**Authors:** Rob Lambkin-Williams, Colin Gelder, Richard Broughton, Corey P. Mallett, Anthony S. Gilbert, Alex Mann, David He, John S. Oxford, David Burt

**Affiliations:** 1 hVIVO Group PLC., Queen Mary BioEnterprises Innovation Centre, London, United Kingdom; 2 University Hospitals Coventry & Warwickshire NHS|| Trust, Clifford Bridge Road, Walsgrave, Coventry, United Kingdom; 3 ID Biomedical Corporation of Québec, 7150 Frederick Banting, Saint-Laurent, Québec, Canada; 4 Analytical Solutions Group, Inc., 14730 Soft Wind Drive, North Potomac, MD, United States of America; 5 Queen Mary’s School of Medicine and Dentistry, London, United Kingdom; Imperial College London, UNITED KINGDOM

## Abstract

**Introduction:**

A Proteosome-adjuvanted trivalent inactivated influenza vaccine (P-TIV) administered intra-nasally was shown to be safe, well tolerated and immunogenic in both systemic and mucosal compartments, and effective at preventing illness associated with evidence of influenza infection.

**Methods:**

In two separate studies using the human viral challenge model, subjects were selected to be immunologically naive to A/Panama/2007/1999 (H3N2) virus and then dosed via nasal spray with one of three regimens of P-TIV or placebo. One or two doses, 15 μg or 30 μg, were given either once only or twice 14 days apart (1 x 30 μg, 2 x 30 μg, 2 x 15 μg) and subjects were challenged with A/Panama/2007/1999 (H3N2) virus. Immune responses to the vaccine antigens were measured by haemagglutination inhibition assay (HAI) and nasal wash secretory IgA (sIgA) antibodies.

**Results:**

Vaccine reactogenicity was mild, predictable and generally consistent with earlier Phase I studies with this vaccine. Seroconversion to A/Panama/2007/1999 (H3N2), following vaccination but prior to challenge, occurred in 57% to 77% of subjects in active dosing groups and 2% of placebo subjects. The greatest relative rise in sIgA, following vaccination but prior to challenge, was observed in groups that received 2 doses.

**Conclusion:**

Intranasal vaccination significantly protected against influenza (as defined by influenza symptoms combined with A/Panama seroconversion) following challenge with A/Panama/2007/1999 (H3N2). When data were pooled from both studies, efficacy ranged from 58% to 82% in active dosing groups for any influenza symptoms with seroconversion, 67% to 85% for systemic or lower respiratory illness and seroconversion, and 65% to 100% for febrile illness and seroconversion. The two dose regimen was found to be superior to the single dose regimen. In this study, protection against illness associated with evidence of influenza infection (evidence determined by seroconversion) following challenge with virus, significantly correlated with pre-challenge HAI titres (p = 0.0003) and mucosal sIgA (p≤0.0001) individually, and HAI (p = 0.028) and sIgA (p = 0.0014) together. HAI and sIgA levels were inversely related to rates of illness.

**Trial Registration:**

ClinicalTrials.gov NCT02522754

## Introduction

Influenza and its associated diseases are a major cause of morbidity and mortality. The United States (US) Advisory Committee on Immunization Practices (ACIP) recommends influenza vaccination for everyone over 6 months of age without contraindications [1]. However, for the 2015–2016 season, the Centers for Disease Control and Prevention reports that national influenza vaccination coverage in the United States reached only 45.6%, slightly lower than the previous season (47.1%). Uptake was highest in the 6–23 months age group (75.3%), and lowest in the 18–49 years age group (32.7%). These rates remain below the US Department of Health and Human Services 'Healthy People 2020 targets' of 70% vaccination coverage for all people over 6 months of age. In addition to developing other preventative and treatment options [2–8], continued efforts are required to ensure that populations with the highest risk of influenza complications (i.e. the elderly, young children, and persons with chronic health conditions) are vaccinated each year.

Intramuscular vaccination can provide systemic antibody-mediated immunity to influenza, yet needle phobia and concern over side effects are among the many reasons people decline such a vaccine.

Although intramuscular administration of viral vaccine antigens has been shown to be effective at preventing influenza infection based primarily on the generation of serum IgG antibodies [9], other correlates of protection are unclear [10] and cellular [11,12] or mucosal immunity [13] may play an important role.

Vaccines administered intranasally have the potential to enhance influenza vaccine acceptability and to enhance mucosal immunity [14,15]. The mucosal immune system consists of a network of lymphoid cells and innate mucosal barriers that may protect against the entry of influenza viruses via the respiratory mucosa, and is a key element in controlling respiratory viral infections [13,16]. The mucosal immune response correlates with reduced infection rate, limited viral replication, and reduced illness in influenza challenge studies [12].

An intranasally administered live attenuated trivalent influenza vaccine is currently licensed as FluMist in the United States and Canada and as Fluenz in Europe. FluMist comprises approximately 10% of the US influenza vaccine supply [17]. For the 2014–2015 season, a quadrivalent vaccine, incorporating H1N1, H3N2 and two B viruses, was launched for both live and inactivated vaccines. However, use of the live vaccine is contraindicated in young children with asthma and/or wheezing, pregnant women, people aged over 49 years, and people with long-term health issues or weakened immune systems. UK government guidelines now recommend Fluenz be administered annually to children from the age of 2 to 7 years [18]. However it is licensed in the EU for up to 18 year olds. Because the live attenuated vaccine must establish infection to elicit an immune response, it may be less effective in older adults and the elderly with substantial immunologic experience with influenza.

An alternative approach to the use of live vaccines is to administer inactivated vaccines intranasally, however this requires mucosal adjuvants in order to elicit sufficiently strong protective immune responses [19–21]. Cholera toxin, *Escherichia coli* heat labile toxin and their variants have been investigated as intranasal vaccine adjuvants, but have been linked to toxicity. There are no adjuvanted intranasal influenza vaccines currently approved for human use [22,23].

Pre-clinical and clinical studies with Proteosome- adjuvanted vaccines administered intranasally have demonstrated strong systemic and mucosal antigen specific immune responses with subsequent protection against disease in several model systems using multiple classes of antigen, including influenza [24–27]. The Proteosome adjuvant system comprises nanoparticles of *Neisseria meningitidis* outer membrane proteins which non-covalently complex with macromolecules, presenting these macromolecules to the mucosal immune system. In mice, intranasal Proteosome-adjuvanted trivalent inactivated influenza vaccine has been shown to induce virus-specific IgG, namely haemagglutination-inhibiting and neutralising antibodies in serum. Additionally, in these studies nasal and pulmonary specific IgA responses were induced and, after intranasal challenge with homologous virus, mice were protected against lethality and weight loss [28,29].

Conducting human viral challenge studies in controlled isolation facilities has provided multiple proof of concepts, including studies with rhinovirus, respiratory syncytial virus [30,31] and influenza [32,33] and has been shown to lead to successful field studies of both vaccines and antivirals [14,24,25].

In this paper we describe two Phase II randomised, double-blind, placebo-controlled studies of an intranasal P-TIV conducted using the human viral challenge model.

Experimental influenza virus infection of healthy volunteers provides a unique opportunity to describe the natural history as the date of infection is known with certainty, nasal virus shedding can be measured, symptoms are recorded prospectively and participants are selected with low pre-haemagglutination inhibition (HAI) antibody titres to ensure statistically significant infection rates with a relatively small number of volunteers [34].

Pioneering studies were performed by Dr David Tyrell at the Common Cold Institute in Salisbury, UK. Volunteers were inoculated by instilling small quantities of influenza virus into the nose to see if this would lead to illness. Challenge studies conducted by the teams of John Treanor and Fred Hayden contributed to the development of the new neuraminidase inhibitors during the 1990s. We began challenge studies in the UK starting in 2001 and have conducted multiple studies with over 1800 volunteers inoculated in multiple proof of concept studies [31,33,34]. The model is well suited to the investigation of novel vaccines such as the P-TIV vaccine described here.

The objectives of both studies described here were (a) to evaluate the safety and tolerability of intranasal administration of P-TIV given in two (first study) or three (second study) dose regimens, (b) to evaluate the magnitude of the immune responses to P-TIV in the serum and mucosal compartments in the different dose regimens, and (c) to develop preliminary data regarding the protective efficacy of P-TIV by evaluating reduction in influenza infection and illness following intranasal challenge with virulent A/Panama/2007/1999 (H3N2).

## Materials and Methods

### Ethics Statement

Two studies, both ethically approved by the North East London Strategic Health Authority, were conducted in accordance with the ethical principles which have their origin in the Declaration of Helsinki, the ICH guidelines for Good Clinical Practice (GCP), and relevant Canadian Food and Drug Regulations. Written informed consent was obtained from all subjects.

### Methodology

Two studies were conducted, one in each of 2002 and 2003. In Study 1 (No. 19896-0001-A-69186), subjects received the intranasal P-TIV vaccine (30 μg per Haemagglutinin antigen) as a one or two-dose regimen, or placebo. Each dose of test article contained the viral Haemagglutinin proteins from A/New Caledonia/20/1999 (H1N1), A/Panama/2007/1999 (H3N2) and B/Victoria/504/2000.

The purpose of Study 2 (No. 19896-0001-A-77383) was to confirm the efficacy of the dose regimens following Study 1 and to evaluate an additional regimen comprising 2 vaccinations of a lower antigen dose (15 μg per antigen). The design of Study 2 was comparable to that of Study 1 which enabled the pooling of efficacy data into a single data set.

Both studies comprised four phases: screening, vaccination, human viral challenge, and follow up. The quarantines were conducted in an adapted hotel environment; other clinical procedures were conducted in clinical suites. Volunteers were recruited primarily from the population of London in accordance with the inclusion / exclusion criteria.

**Fig 1:**
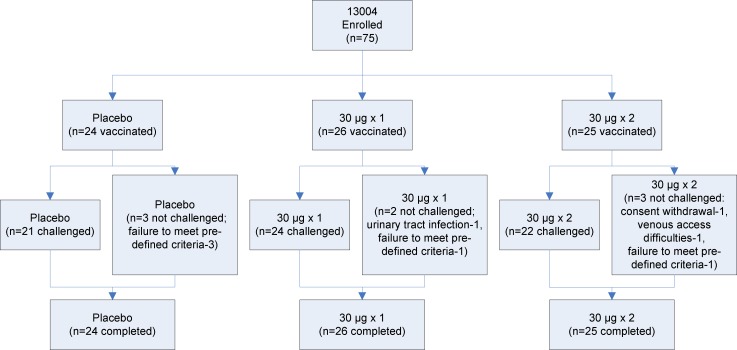
CONSORT Flowchart for Study 1 (Study Protocol 13004)

**Fig 2:**
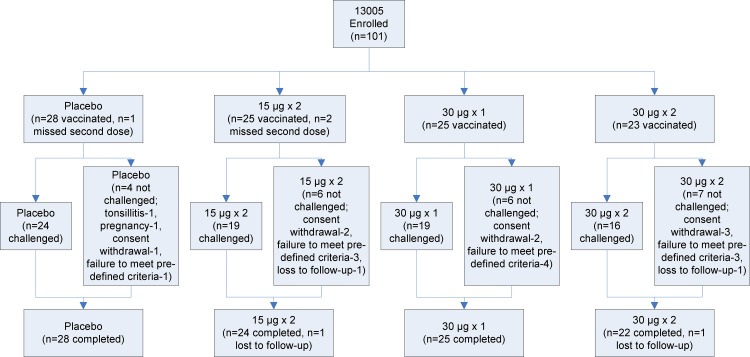
CONSORT Flowchart for Study 2 (Study Protocol 13005)

**Fig 3:**
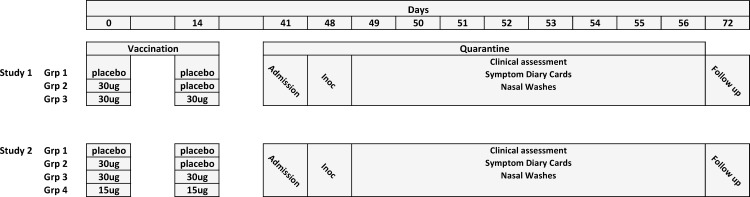
Summary of Study Design for both studies

An overview of the study design is shown in Figs 1–3

### Screening

All subjects attended a screening visit to determine eligibility for the study and susceptibility to the challenge virus A/Panama/2007/1999(H3N2). Subjects were volunteers aged 18–50 years inclusive who were willing to forego the licensed seasonal influenza vaccine until after study completion and provide written informed consent.

Subjects were in good general health as determined by medical history, physical examination, serology (HIV and Hepatitis B and C) and clinical laboratory tests. Female subjects were required to provide a history of reliable contraceptive practice. Susceptibility to A/Panama/2007/1999 (H3N2) (a serum reciprocal HAI titre ≤10) was confirmed at screening. Exclusion criteria included asthma, hypersensitivity to mercurials or chicken eggs, anatomic or neurologic abnormality impairing the gag reflex or contributing to aspiration, chronic nasopharyngeal complaints, abnormal electrocardiogram (ECG), febrile illness or significant symptoms of upper respiratory infection on the day of vaccination or between admission to quarantine and administration of the challenge inoculum.

Subjects were excluded if they used medication or other products for rhinitis or nasal congestion, had received systemic glucocorticoids within 1 month, or cytotoxic or immunosuppressive drugs within 6 months of the study start. Subjects agreed not to smoke during the quarantine phase.

### Method of Assigning Patients to Treatment Groups For Both Studies

On Study Day 0, the investigational pharmacist who had no involvement whatsoever with any clinical outcome measurement was provided with each eligible and consenting subject’s initials, subject ID number (SID), and gender. The investigational pharmacist then employed the gender-specific randomization list provided by the sponsor to assign the next available randomization code and to determine treatment assignment. For each subject randomized, a label bearing the subject’s SID, initials, and randomization code was then prepared and affixed to a metered dose pump. The investigational pharmacist then prepared the appropriate test article for each subject.

For Day 14 doses, the investigational pharmacist followed the same procedure described above.

### Blinding

At the study site, neither study subjects nor investigative site personnel (including the Investigator) were aware of treatment assignment. To ensure the blind of investigative site staff, an investigational pharmacist was designated for this study. The person who served as investigational pharmacist was responsible for application of the randomization, as well as maintenance of the treatment log, test article dose preparation, and day-to-day test article accountability. This person did not perform any clinical safety or efficacy observations during the study. The dark amber reservoir vials of the nasal spray devices and the low test article volumes effectively obscured any differentiation of the slightly opalescent vaccine from the clear placebo by blinded site staff.

Only the sponsor's Study Director, and a representative of the sponsor whose sole responsibility was to review pharmacy documents and test article inventories, had access to subject treatment assignments. Clinical monitoring staff, data management staff, and laboratory staff remained blinded throughout the study.

The initiation packet for each site clearly stated these blinding rules. The initiation packets also specified that in an emergency, the person acting as pharmacist would be allowed to un-blind. In the event of a non-emergent need to unblind, the sponsor requested a call to confer prior to unblinding.

### Vaccination Phase

Eligible subjects attended on two occasions (Day 0 and Day 14 (± 1 day)) for vaccination with the test article or placebo preparations according to the randomisation. Each dose of P-TIV (containing either 15 μg or 30 μg of each of three viral haemagglutinins) was delivered to the nasal mucosae using a nasal sprayer delivering droplets sized to impact in the nasopharynx as described below. Subjects were randomised to one of four dosing regimens:

- 2 x placebo (Studies 1 and 2)
- 1 x 30 μg (placebo as a second dose) (Studies 1 and 2)
- 2 x 30 μg (Studies 1 and 2)
- 2 x 15 μg (Study 2)

### Test Article and Placebo

P-TIV was formulated as described previously.

Stock solutions of P-TIV were appropriately diluted with 10 mM Na/K phosphate isotonic saline, pH 7.4, and 0.01% thimerosal as a preservative. Each 3 mL vial was then filled with 0.9 mL vaccine containing 107 ± 27 μg/mL or 54 ± 13 μg/mL of inactivated detergent split virion vaccine from each of A/New Caledonia/20/1999 (H1N1), A/Panama/2007/1999 (H3N2) and either B/Victoria/504/2000 (Study 1) or B/Shangdong/7/1997 (Study 2) non-covalently bound to Proteosomes, thus respectively producing the 30 μg and 15 μg dosages. Proteosome to HA ratio was approximately 4:1 (the total ratio in the final vaccine ranged from 2.5 to 5.1). Each vial also contained non-HA viral proteins and trace amounts of egg proteins.

Placebo was 10 mL of 10 mM Na/K phosphate buffered isotonic saline, pH 7.4, mixed with 0.01% thimerosal in each 20 mL vial.

### Vaccine Delivery

On the day of vaccination, test article solution was transferred to a Valois VP3/140 nasal spray pump comprising a screw-top vial, actuator, and nosepiece. Each full depression of the actuator delivered a metered dose of liquid as an atomised spray. Delivery was within ± 5% of nominal volume with each discharge, and the median droplet size in the spray was 40–50 μm. Each subject received a dose volume of 140 μL per nostril, (total volume delivered was 280 μL).

### Clinical evaluations

Clinical evaluations were performed before and after each test article dose, on Day 28 (± 2) and Day 39 (± 3). Specimens were collected for clinical chemistry, haematology and urinalysis on Day 28 (± 2) and for assessment of immune response before each test article dose, and on Days 28 (± 2) and 39 (± 3).

### Vaccine Reactogenicity

Subjects were monitored for potential vaccine reactogenicity (common, “expected” adverse reactions to the vaccine, especially excessive immunological responses and associated signs and symptoms) by recording objective findings (via physician examination) and subjective complaints (via subject diaries) during the seven days following each vaccination.

In Study 1, reactogenicity data were collected by interview two days after each dose and by scripted telephone contact seven days after each dose. The nose, throat, ears, and cervical nodes were examined seven days after each dose. In Study 2, reactogenicity data were collected seven days after each dose. Subjects were instructed to report to the clinic if they had moderate to severe complaints. Clinical laboratory tests were repeated on Day 28 (± 3 days). Adverse events (AEs) and changes in concomitant medications were recorded up to Day 39 (± 3 days) following the first dose, and again on Day 56–72.

Reactogenicity complaints and findings listed in the data capture recording forms, solicited by scripted interview or by directed examinations were those reasonably expected to be associated with a nasal vaccine, and were presumed to be drug-related. Immediate complaints were categorized as 1) burning or stinging in the nose, 2) burning or stinging in the throat, 3) itching in the nose, throat or eyes, 4) shortness of breath, 5) light-headedness or dizziness, 6) a new rash that has become itchy and 7) feverishness.

Reactogenicity was self-reported by the subjects and graded as follows:

Grade 1: They noticed the symptom but it did not affect their usual activities significantly.
Grade 2: The symptom was sufficiently bad that they couldn't do a significant part of their usual activities.
Grade 3: The severity of the symptoms was such they were not able to do most of their usual activities.

Immediate and reactogenicity complaints were tabulated separately and only additionally reported as AEs or serious AEs (SAEs) if they a) fulfilled the definition of a SAE, b) persisted beyond seven days after test article administration, or c) were not considered (by the Investigator) to be related to the test article.

### Human Viral Challenge Phase

On Day 39 (± 3 days), subjects who were in good health and without evidence of fever or upper respiratory symptoms were selected for the human viral challenge phase. Eligible subjects were isolated in quarantine and monitored for signs of community acquired illness for two days, before being challenged on Day 41 (± 3 days) with A/Panama/2007/1999 (H3N2). The inoculum was administered by intranasal drops and contained approximately 10^5.5^ EID_50_ (egg infectious dose) of infectious influenza virus, planned to give an ‘Attack rate’ of ≥70% in challenged subjects who had received placebo during the vaccination phase.

Subjects remained in the quarantine unit for a further seven days post viral challenge. Twice daily clinical evaluations for influenza-like illness (symptom diary card and interview) were performed from the evening of Day 41 (± 3 days) through Day 48 (± 3 days). Diary card symptoms recorded included runny nose, stuffy nose, sore throat, earache, malaise, cough, headache, muscle and/or joint ache. Oral temperatures were recorded four times daily and examination of the ears, nose, throat and chest was conducted once daily. Paper tissues were distributed daily during quarantine and collected every 24 hours in order to count the number of tissues used and the weight of the mucus-containing tissues. Treatment with paracetamol (acetaminophen) or oseltamivir was permitted if certain fever and symptom criteria were met.

On Day 47 (± 3 days), subjects began treatment with oseltamivir to ensure virus clearance prior to discharge from quarantine.

### Follow up Phase

The follow-up visits on Day 56–72 included a clinical evaluation and serum sampling for antibody responses to the challenge agent.

### Influenza Illness definitions

Influenza Illness was defined using the following criteria:

#### Fever

Any oral temperature ≥ 37.9°C confirmed by repeat observation not less than 20 minutes and not more than 60 minutes later.

#### Upper respiratory illness

Self-reported moderate or severe rhinorrhea, nasal congestion, or sore throat and/or physician findings of nasal discharge, pharyngitis, otitis or sinusitis on at least two consecutive days, at least one day of which must feature grade 2 severity, or if any of the above attain grade 3 severity once.

#### Lower respiratory illness

Moderate or severe cough and/or physician findings of new râles, rhonchi, or wheezing on at least two consecutive days, at least one day of which must feature grade 2 severity, or if any of the above attain grade 3 severity once.

#### Systemic illness

Moderate or severe headache or myalgia or arthralgia at least once in each of two consecutive days, at least one day of which must feature grade 2 severity, or if any of the above attain grade 3 severity once.

#### Any Illness

Fever, upper respiratory illness, lower respiratory illness, or systemic illness or any combination thereof.

#### Seroconversion

Seroconversion is defined as the change of a serologic test from negative to positive, indicating the development of antibodies in response to infection or immunization. It is defined as a four-fold increase in the antibody titre generated by an antigen whether that be a vaccine or an infection.

In the study we use seroconversion to both describe the effect of the vaccine and challenge virus.

For the vaccine, the samples used are from day 0 to day 39 (+/-3).

When describing seroconversion as result of viral challenge we use samples from Day 39 (+/-3), two days prior to challenge, to the volunteers’ last visit Day 57 to Day 72. We used seroconversion as a surrogate marker of infection. We were unable to use tissue culture as an additional confirmation due to technical difficulties. We accept viral data would have been useful, but seroconversion is widely accepted as a surrogate.

### Immunological Assays

Nasal wash specimens for specific secretory IgA (sIgA) and sera for specific antibody determinations were collected at baseline and on Day 28 and Day 39 (± 3 days) following vaccination and on Day 56 to 72 following challenge. Systemic antibody responses were determined by serum haemagglutination (HAI) inhibition antibody titres specific for the three vaccine strains. Sera were tested by the standard haemagglutination inhibition assay with turkey erythrocytes after treatment with receptor-destroying enzyme to remove non-specific inhibitors of agglutination [24].

Nasal wash samples were collected as previously described [24]. Nasal washes for sIgA assays were subsequently concentrated using Centricon centrifugal filter devices (50,000 molecular weight cut-off) as described by the manufacturer (Millipore Corporation, Billerica, MA). Vaccine strain-specific sIgA responses were measured in concentrated nasal wash by kinetic enzyme-linked immunosorbent assay (KELISA) using biotin-conjugated goat anti-human secretory piece (Nordic Immunological Laboratories, Tilburg, The Netherlands) in tandem with peroxidase-conjugated ExtrAvidin (Sigma-Aldrich, Inc. St. Louis, MO) as previously described [24]. The plates were coated with inactivated whole virus. KELISA plates were read on a Benchmark Plus Microplate spectrophotometer using Microplate Manager software (version 5.2, build 103) to generate a KELISA rate for each sample as previously described [24]. Total sIgA levels in concentrated nasal wash were quantified by radial figure immunodiffusion using BINDARID kits as described by the manufacturer (The Binding Site Ltd., Birmingham, UK). Vaccine strain-specific sIgA KELISA rates in each sample were then normalised to the arithmetic mean of total sIgA determinations for all specimens from the entire study population [24]. Note that arithmetic means used here are for calculation of individual values and are not to be confused with geometric means used for group analyses.

The assays for both HAI and sIgA were paired so that for any given subject, both assays were performed at the same time.

### Statistics

#### Sample Size and Randomisation

Study 1 was designed to have 80% power to detect a moderately frequent occurring safety event, more specifically, to detect at least one event in any given group of 25 subjects for a safety event with 6% probability of occurrence in a single subject, or at least one event in all P-TIV treated subjects (N = 50) for a safety event with 3% probability of occurrence in a single subject. With regard to the flu challenge efficacy outcome, assuming a ≥ 60% yield of the binary outcome “any illness” from the virus challenge inoculum, a group size of ≥20 would yield ≥80% power in detecting a ≥70% reduction in the rate of a binary flu outcome.

Study 2 was designed to have 80% power to detect a moderately frequently occurring safety event, more specifically, to detect at least one event in any given group of 28 subjects for a safety event with 6% probability of occurrence in a single subject, or at least one event in all P-TIV treated subjects (N = 84) for a safety event with 2% probability of occurrence in a single subject. With regard to the flu challenge efficacy outcome, assuming an approximately 50–60% yield of the binary outcome “any illness” from the virus challenge inoculum, a group size of 28 would yield ≥80% power in detecting a ≥70% reduction in the rate of a binary flu outcome.

#### Analyses

Statistical analyses were performed using SAS/STAT, version 9.2 (SAS Institute, Cary, NC). As these two early clinical phase studies were not used to provide definitive evidence of efficacy or safety, p-values of less than 0.05 for two-sided statistical tests were considered significant. Multiple comparison corrections were not considered. All the analyses were based on the observed data only. No attempt was made to impute missing safety, immunogenicity or efficacy data.

Categorical demographic, immunogenicity, efficacy, tissue count/weight, and reactogenicity variables were summarised by frequency distributions (counts and percentage) of subjects within each category and treatment. Continuous demographic variables were summarised with descriptive statistics stratified by treatment. Continuous immunogenicity variables were summarised by geometric means (GM) and 95% confidence intervals around the GMs, stratified by treatment, visit and strain. Adverse events were tabulated by COSTART body system and preferred terms. Subject counts and percentages within each AE type were determined separately within each treatment group.

#### Data Pooling

The potential to pool the data from the two studies in demographic, immunogenicity and efficacy variables was examined. For continuous variables, treatment-by-study interaction was evaluated using generalized linear model models. For binary variables, the test of homogeneity was performed to examine if the treatment effect varied by study. In immunogenicity analyses using the pooled ITT sample (assuming the data can be pooled), Log10-transformed HAI titre and sIgA fold rise outcomes at each visit/strain were modeled with generalized linear models adjusted for study and log10-transformed baseline outcome parameter values (latter when appropriate). For binary responders in HAI outcome, the responder rates were compared by treatment using Cochran-Mantel-Haenszel chi-square tests for nominal and ordinal data, separately by visit and strain. Efficacy influenza challenge outcomes and safety outcomes such as adverse events and reactogenicity were all binary in nature and were handled similarly to that of the HAI responder data. In the analysis to correlate immunogenicity and efficacy outcomes, logistic regression models were used with flu as outcome and log10-transformed HAI titres and sIgA signal prior to influenza challenge as predictors. Efficacy analyses were based on the influenza challenge outcome of influenza symptoms and seroconversion.

### Study Population

Eighty eight male and 88 female subjects were enrolled and vaccinated, with 145 completing the Human Viral Challenge phase (74 females, 71 males). Mean age of subjects enrolled was 25.7 years (SD: 5.7), and subjects challenged was 25.5 years (SD:5.9). Demographic profiles were substantively the same for enrolled and challenged subjects.

A total of 52 subjects received two doses of placebo (24 and 28 in Study 1 and Study 2 respectively) with 45 subsequently being challenged with virus (21 and 24 respectively).

A total of 48 subjects received two doses of 30 μg vaccine (25 and 23 in Study 1 and Study 2 respectively) with 38 subsequently being challenged with virus (22 and 16 respectively).

A total of 51 subjects received one dose of 30 μg vaccine (26 and 25 in Study 1 and Study 2 respectively) with 43 subsequently being challenged with virus (24 and 19 respectively).

A total of 25 subjects received two doses of 15 μg vaccine (Study 2 only) with 19 subsequently being challenged with virus.

Schematics of the dosing regimens for Studies 1 and 2 are shown respectively in Fig 3.

### Vaccine Safety

#### Immediate Complaints

All immediate complaints (Study 1: 22; Study 2: 52) were mild in severity. Twenty were reported in the 15 μg x 2 dose group (Study 2), 17 were reported in the 30 μg x 1 dose (Study 1: 5; Study 2: 12), 11 were reported in the 30 μg x 2 dose group (Study 1: 6, Study 2: 5), and 26 in the placebo group (Study 1: 11; Study 2: 15).

Burning or stinging in the nose (24 complaints), and light-headedness or dizziness (23 complaints) were most commonly reported. Itching in the nose, throat or eyes (16 complaints) and burning or stinging in the throat (6 complaints) were less frequently reported. There were 3 complaints of new rash or rash that has become itchy (2 active dose, 1 placebo).

#### Reactogenicity

Reactogenicity was largely predictable in both studies. Reactogenicity was evaluated via patient diaries (Table 1) and physician examination (Table 2) following doses of test article. Predictably, upper respiratory conditions (nasal discharge, nasal congestion, sneezing, sore throat, and cough) were most commonly reported. Compared to past studies, there was less nasal mucosal inflammation and more cervical adenopathy but for both these findings, no differences between placebo and active treatments.

**Table 1:** Patient Diary Recorded Reactogenicity Following Dose 1 and Dose 2 of Vaccine Pooled Data.

	After Dose 1				After Dose 2			
	Treatment Group				Treatment Group			
Dose	Placebo	15 μg x 2	30 μg x 1	30 μg x 2	Placebo	15 μg x 2	30 μg x 1	30 μg x 2
N	52 (%)	25 (%)	51 (%)	48 (%)	52 (%)	25 (%)	51 (%)	48 (%)
Malaise/ Fatigue	13 (25.0)	4 (16.0)	8 (15.6)	12 (25.0)	8 (15.3)	3 (12.0)	5 (9.8)	12 (25.0)
Anorexia	6 (11.5)	3 (12.0)	6 (11.7)	2 (4.1)	4 (7.6)	2 (8.0)	3 (5.8)	2 (4.1)
Headache	15 (28.8)	6 (24.0)	15 (29.4)	13 (27.0)	9 (17.3)	4 (16.0)	9 (17.6)	9 (18.7)
Myalgia/Arthralgia	3 (5.7)	5 (20.0)	5 (9.8)	4 (8.3)	4 (7.6)	4 (16.0)	1 (1.9)	5 (10.4)
Nasal discharge	14 (26.9)	6 (24.0)	15 (29.4)	22 (45.8)	12 (23.0)	5 (20.0)	13 (25.4)	21 (43.7)
Nasal congestion	15 (28.8)	8 (32.0)	20 (39.2)	21 (43.7)	14 (26.9)	8 (32.0)	13 (25.4)	18 (37.5)
Nasal sting/burn/itch	6 (11.5)	2 (8.0)	4 (7.8)	0	6 (11.5)	3 (12.0)	2 (3.9)	3 (6.2)
Nose bleed	2 (3.8)	0	2 (3.9)	1 (2.0)	2 (3.8)	0	1 (1.9)	1 (2.0)
Red eyes	4 (7.6)	3 (12.0)	3 (5.8)	6 (12.5)	2 (3.8)	3 (12.0)	1 (1.9)	7 (14.5)
Sneezing	16 (30.7)	6 (24.0)	14 (27.4)	14 (29.1)	11 (21.1)	20 (80.0)	10 (19.6)	17 (35.4)
Sore throat	15 (28.8)	4 (16.0)	6 (11.7)	10 (20.8)	11 (21.1)	4 (16.0)	12 (23.5)	3 (20.8)
Cough	10 (19.2)	3 (12.0)	4 (7.8)	8 (16.6)	6 (11.5)	4 (16.0)	12 (23.5)	10 (20.8)
Wheezing or short of breath	3 (5.7)	0	1 (1.9)	0	0	0	0	1 (2.0)

**Table 2:** Physician Examination Reactogenicity in the 7 Days Following Dose 1 and Dose 2 of Vaccine Pooled Data.

	After Dose 1				After Dose 2			
	Treatment Group				Treatment Group			
Dose	Placebo	15 μg x 2	30 μg x 1	30 μg x 2	Placebo	15 μg x 2	30 μg x 1	30 μg x 2
N	52 (%)	25 (%)	51 (%)	48 (%)	52 (%)	25 (%)	51 (%)	48 (%)
Nasal mucosal inflammation	0	0	0	0	1 (1.9)	0	0	1 (2.0)
Nasal discharge	7 (13.4)	1 (4.0)	9 (17.6)	10 (20.8)	5 (10.4)	3 (12.0)	7 (13.7)	9 (18.7)
Pharyngeal inflammation	4 (7.6)	0	1 (1.9)	1 (2.0)	1 (1.9)	0	2 (3.9)	2 (3.9)
Sinusitis	0	0	0	0	0	0	1 (1.9)	0
Cervical/ Post-auricular nodes	10 (19.2)	0	7 (13.7)	10 (20.8)	8 (15.3)	1 (4.0)	13 (25.4)	10 (20.8)
Otic inflammation	1 (1.9)	0	0	0	2 (3.8)	2 (8.0)	3 (5.8)	2 (3.9)

There was no consistent difference in frequency of complaints following placebo or active treatments except for small, non-significant trends toward increased nasal discharge and nasal congestion following active treatments.

There was no apparent worsening of findings following the second dose and a slight trend toward reduced subject reported reactogenicity.

### Adverse Events

There were no SAEs in either study. The AE profile was unremarkable (Table 3). Most frequently reported treatment emergent AEs were respiratory, gastro-intestinal, and thoracic and mediastinal, with no significant differences in the incidence of AEs within system organ classes (SOC) between groups.

**Table 3:** Overall summary of frequency of adverse events by treatment group.

	Treatment Group									
	Placebo			Vaccine Dose Regimen						
				15 μg x 2	30 μg x 1			30 μg x 2		
	Study 1	Study 2	Total	Study 2	Study 1	Study 2	Total	Study 1	Study 2	Total
Number of subjects in group	24	28	52	25	26	25	51	25	23	48
Total number of adverse events	41	23	64	18	41	33	74	50	15	65
Number of subjects with at least 1 adverse event	17 (70.8%)	12 (42.8%)	29 (55.7%)	11 (44.0%)	17 (65.3%)	14 (56.0%)	30 (58.8%)	17 (68.0%)	10 (43.4%)	27 (56.2%)
Number of subjects with at least 1 possibly or probably drug related adverse event	6 (25.0%)	2 (7.1%)	8 (15.3%)	2 (8.0%)	5 (19.2%)	1 (4.0%)	6 (11.7%)	9 (36.0%)	5 (21.7%)	14 (29.1%)

Twelve AEs were severe. Seven of these were reported by one subject in Study 1, and included a cluster of upper respiratory symptoms (sneezing and conjunctival infection) with fever, abdominal pains, diarrhoea, and multiple systemic complaints; the latter were attributed by the investigator to an unrelated intercurrent viral gastroenteritis. The other five severe AEs were in Study 2 and consisted of tonsillitis, pyelonephritis, fatigue, flu-like illness (in a non-challenged subject) and conjunctivitis. All of these were deemed probably not related, or possibly related, to study drug.

In Study 2, there were notably fewer AEs across all dosing groups than in Study 1. Both studies showed respiratory ailments comprising the largest proportion of AEs but the relative proportion was lower in Study 2 (35/89 (39%) vs. 80/132 (61%) of AEs in Study 1), with no apparent differences between dosing groups. An overall summary of AE frequency is shown in Table 3. Overall, there was a higher incidence of drug related AEs in the 30 μg x 2 dose group compared to other active dose groups and placebo.

### Vaccine efficacy: Immunogenicity

#### Immune response: Serum Haemagglutination-Inhibiting Antibodies

Compared to placebo subjects, significantly more subjects in the vaccine treatment groups showed an increase in HAI titre following immunisation with the intranasal vaccine (Table 4). At Day 39, significant (p<0.0001) increases in HAI titre and percentage of subjects showing a ≥4 fold rise in HAI titre, specific to A/Panama/2007/1999 (H3N2), were seen across all active dose groups, with more subjects in those groups that received two active doses groups showing a 4-fold rise than in the single active dose group. Significant (p<0.0001) increases in HAI titre and percentage of subjects showing a ≥4-fold rise in HAI titre, specific to A/New Caledonia/20/1999 (H1N1), were also clearly demonstrated. Responses to the B strains were lower.

**Table 4:** Serum HAI antibody responses at Day 39±3 following first nasal administration of trivalent Proteosome-influenza vaccine.

Dose group	A/New Caledonia (H1N1)			A/Panama (H3N2)			B Strain†		
	Geometric mean titre (95% CI)		4-fold rise (%)	Geometric mean titre (95% CI)		4-fold rise (%)	Geometric mean titre (95% CI)		4-fold rise (%)
	Day 0	Day 39±3		Day 0	Day 39±3		Day 0	Day 39±3	
Placebo	7.6 (5.9–9.8)	9.1 (6.8–12.1)	16	7.3 (6.4–8.2)	8.2 (6.8–9.9)	8	6.8 (5.6–8.1)	7.5 (6.2–9.2)	8
15 μg x 2	6.3 (5.1–7.8)	16.4 (9.9–27.3)*	48*	7.6 (6.4–9.0)	33.9 (20.2–56.9)**	67**	5.6 (5.0–6.3)	7.0 (5.4–9.0)	10
30 μg x 1	7.7 (6.1–9.6)	24.2 (16.0–36.6)**	60**	7.3 (6.4–8.2)	25.7 (18.3–36.0)**	55**	6.1 (5.4–6.9)	9.4 (7.4–11.9)*	19
30 μg x 2	9.5 (7.4–12.2)	36.7 (23.5–57.3)**	75**	7.7 (6.8–8.7)	41.4 (29.0–59.0)**	75**	6.7 (5.6–7.9)	10.0 (7.4–13.6)	23

†B Strain: B/Victoria in Study 13004, B/Shandong in Study 13005.

*p< 0.05 compared to placebo

**p< 0.0001 compared to placebo

For all strains, and for both HAI titres and percentages of subjects showing a ≥4 fold rise in HAI titre, there were no significant differences between active two dose and single dose groups, except that HAI titres to A/Panama/2007/1999 (H3N2) in the 30 μg x 2 group were significantly greater than in the 30 μg x 1 group (p = 0.032).

#### Immune response: Nasal sIgA

Compared to placebo, there were significant (p<0.0001) geometric mean fold rises in nasal sIgA specific to A/Panama/2007/1999 (H3N2) (Table 5) at Day 39 for both active two dose groups, with the greatest increase in the 15 μg x 2 treatment group. The increase was lowest in the 30 μg x 1 treatment group although still significant (p = 0.0002).

**Table 5:** Nasal virus-specific secretory IgA responses at Day 39±3 after first nasal administration of trivalent Proteosome influenza vaccine.

Dose group	A/New Caledonia (H1N1)	A/Panama (H3N2)	B Strain†
	Geometric mean fold rise (95% CI)	Geometric mean fold rise (95% CI)	Geometric mean fold rise (95% CI)
	Day 39±3	Day 39±3	Day 39±3
Placebo	0.93 (0.77–1.12)	1.04 (0.90–1.21)	1.02 (0.88–1.17)
15 μg x 2	4.08 (2.87–5.7)**	4.07 (3.10–5.36)**	2.66 (2.03–3.49)**
30 μg x 1	1.50 (1.13–1.99)*	1.66 (1.33–2.08)*	1.24 (1.03–1.51)*
30 μg x 2	3.93 (2.97–5.”20)**	3.28 (2.46–4.37)**	2.45 (1.91–3.14)**

†B Strain: B/Victoria in Study 1, B/Shandong in Study 2

*p< 0.05 compared to placebo

**p< 0.0001 compared to placebo

There were also significant (p<0.0001) geometric mean fold rises in nasal sIgA specific to A/New Caledonia/20/1999 (H1N1) and the B strains (B/Victoria in Study 1 and B/Shandong in Study 2) at Day 39 for both active two dose groups, with the greatest increases in the 15 μg x 2 treatment group. The increases were lowest in the 30 μg x 1 treatment groups but still significant, p = 0.0007 for A/New Caledonia/20/99 and p = 0.026 for the B strains.

The active two dose groups both showed significantly (p<0.0001) higher increases than the single active dose group for all three strains.

### Vaccine efficacy: Clinical

**Fig 4:**
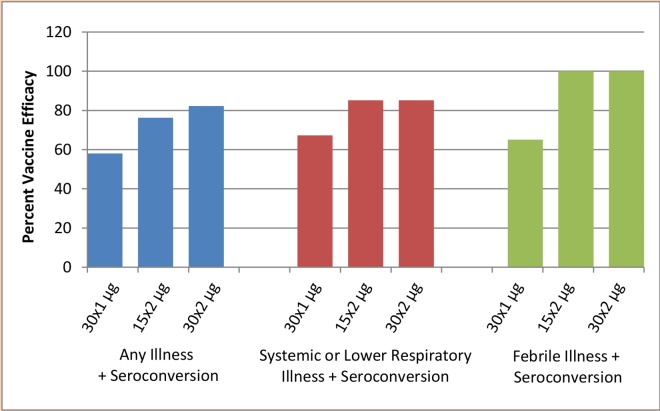
Percentage vaccine efficacy by treatment group

Vaccination with both antigen dose concentrations, whether one or two vaccinations, significantly protected against illness associated with evidence of influenza. The two studies were pooled and efficacy calculated by contrasting vaccine illness incidence rates with those observed in placebo recipients (Table 6). The incidence of any influenza symptoms and seroconversion after influenza challenge was 20/45 (44%), 2/19 (11%), 8/43 (19%) and 3/38 (8%) respectively for the placebo, 15 x 2 μg, 30 x 1 μg and 30 x 2 μg dosing groups. The incidence of systemic or lower respiratory illness and seroconversion was 16/45 (36%), 1/19 (5%), 5/43 (12%) and 2/38 (5%) respectively for the placebo, 15 x 2 μg, 30 x 1 μg and 30 x 2 μg dosing groups. The incidence of febrile illness and seroconversion was 9/45 (20%), 0/19 (0%), 3/43 (7%) and 0/38 (0%) respectively for the placebo, 15 x 2 μg, 30 x 1 μg and 30 x 2 μg dosing groups. Correspondingly, efficacy between active dosing groups ranged from 58% to 82% for any illness and seroconversion, 67% to 85% for systemic or lower respiratory illness and seroconversion, and 65% to 100% for febrile illness and seroconversion. The two dose regimen was generally superior to the one dose regimen (see Fig 4).

**Table 6:** Proportion of subjects in each treatment group meeting specified post-challenge endpoints and apparent efficacy versus Placebo (pooled data).

	Treatment group						
	Placebo	15 μg x 2		30 μg x 1		30 μg x 2	
	N = 45	N = 19		N = 43		N = 38	
Endpoint	No. subjects affected	No. subjects affected	Efficacy %	No. subjects affected	Efficacy %	No. subjects affected	Efficacy %
Any Illness	21	3*	66.2	18	10.3	10	43.6
Any illness and seroconversion	20	2*	76.3	8*	58.1	3*	82.2
Systemic or lower respiratory illness and seroconversion	16	1*	85.2	5*	67.3	2*	85.2
Febrile illness and seroconversion	9	0*	100.0	3	65.1	0*	100.0

Efficacy % = 1- [Attack rate (Vaccinated group)/(Attack rate(placebo group)]

* P<0.05

A listing by dose of the incidence of post-challenge symptoms is provided in Table 7.

**Table 7:** Incidence of Post-Challenge Symptoms on Days 42–49 by Dose: Pooled Data.

	Treatment Group			
Dose	Placebo	15 μg x 2	30 μg x 1	30 μg x 2
N	45	19	43	38
Efficacy Definition	n (%)	n (%)	n (%)	n (%)
Upper Respiratory Symptoms	17 (37.8)	1 (5.3)	17 (39.5)	7 (18.4)
Lower Respiratory Symptoms	2 (4.4)	0	4 (9.3)	1 (2.6)
Systemic Symptoms	17 (37.8)	2 (10.5)	9 (20.9)	3 (7.9)
Fever	9 (20.0)	0	5 (11.6)	0
Any Symptoms	21 (46.7)	3 (15.8)	18 (41.9)	10 (26.3)
Seroconversion	38 (84.4)	6 (31.6)	16 (37.2)	5 (13.2)
Fever and Systemic Symptoms	8 (17.8)	0	4 (9.3)	0
Upper Respiratory and Systemic Symptoms	14 (31.1)	0	8 (18.6)	0
Fever and Upper Respiratory Symptoms	7 (15.6)	0	5 (11.6)	0
Seroconversion and Any Symptoms	20 (44.4)	2 (10.5)	8 (18.6)	3 (7.9)
Seroconversion and Systemic Symptoms	16 (35.6)	1 (5.3)	4 (9.3)	2 (5.3)
Seroconversion and Upper Respiratory Symptoms	17 (37.8)	1 (5.3)	8 (18.6)	1 (2.6)
Seroconversion and Fever	9 (20.0)	0	3 (7.0)	0
Seroconversion and (Lower Respiratory or Systemic Symptoms)	16 (35.6)	1 (5.3)	5 (11.6)	2 (5.3)

### Indirect assessment of functional illness

**Fig 5:**
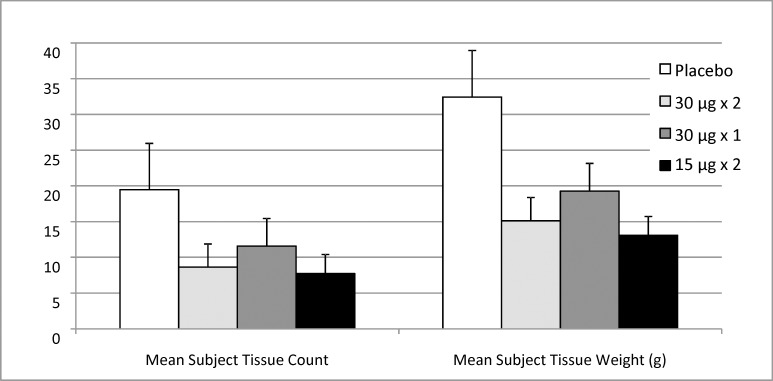
Mean (± SE) Tissue count and tissue weight for all treatment groups in Study 2

In Study 2, the efficacy of the vaccine against functional illness severity was indirectly assessed by used tissue count and mucus weight (Fig 5). Used tissue count and tissue weight were both highest in the placebo group followed by the single 30 μg dose treatment group.

### Correlates of protection

**Fig 6:**
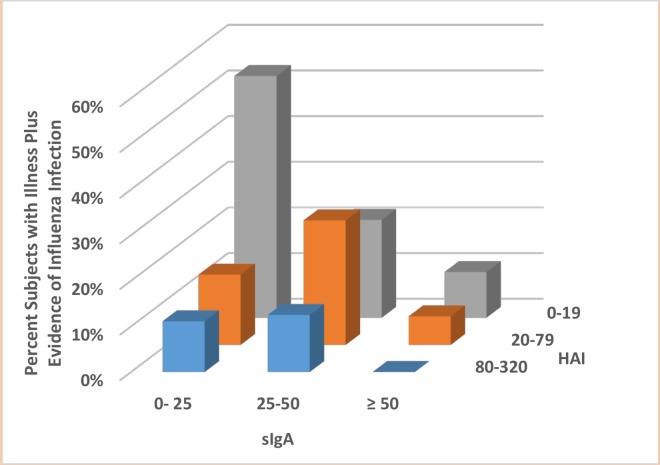
Correlation of serum IgG (HAI) and mucosal sIgA levels at Day 39±3 and rates of any influenza symptoms and seroconversion following viral challenge

Significant associations were observed between immunogenicity on Day 39 and efficacy. Levels of HAI and sIgA, either individually or together, were inversely correlated with rates of seroconversion after exposure (logistic regression for one covariant: p = 0.0003 (HAI) and p = 0.0001 (sIgA); and for two covariates: p = 0.0028 (HAI) and p = 0.0014 (sIgA)). A three dimensional plot of this effect using immunology data from Day 39 is shown in Fig 6.

## Discussion

This was not a field study, but rather a human viral challenge study, where the time of exposure to influenza was known and the time of observation and sampling were controlled.

In the two studies reported here, intranasal Proteosome-adjuvanted vaccine given as either a single dose (30 μg per antigen) or two doses (30 μg x 2 or 15 μg x 2 per antigen) was demonstrated to be safe and well tolerated. Immediate complaints after dosing and reactogenicity were generally mild and predictable, with no clear differentiation of vaccine from placebo.

The vaccine elicited serum and mucosal responses to all three vaccine antigens, i.e. A/New Caledonia/20/1999 (H1N1), A/Panama/2007/1999 (H3N2) and B/Victoria/504/2000 (Study 1), or B/Shangdong/7/1997 (Study 2).

The vaccine protected against illness associated with evidence of A/Panama influenza infection (evidence determined by seroconversion) following challenge with virus. Efficacy increased as endpoints became more specific and more severe, an expected finding in most challenge studies. In the active two dose groups, efficacy was 44% to 66% for any influenza symptoms, 76% to 82% for any influenza symptoms with seroconversion, 85% for systemic or lower respiratory illness with seroconversion and 100% for febrile illness with seroconversion. The efficacy data were independently corroborated by an indirect functional efficacy parameter, i.e. used tissue count and weight.

This study also demonstrates that both serum HAI titre and sIgA are important correlates of protection against illness associated with evidence of influenza, following challenge with virus. Serum HAI and sIgA levels, either individually or together, were significantly inversely correlated with rates of any influenza symptoms combined with seroconversion following challenge with virus.

Two doses of vaccine were more effective than a single dose, even if total dosage was the same. This was reflected in increased immune responses to the active two dose groups compared to the single dose. Interestingly the mucosal sIgA response to the two dose regimens, unlike the HAI response, was significantly higher than to the single dose regimen (and for all strains).

Following this study, a field study was conducted in Canada during the 2003–2004 flu season whereby one of three doses (placebo, 15 μg x 2 or 30 μg x 1) was administered to 1349 adults aged 18 to 64 [35]. As the field study was conducted shortly after the second challenge study, the same three strains were used in both studies (A/New Caledonia/20/1999 (H1N1), A/Panama/2007/1999 (H3N2), B/Shangdong/7/1997). In the field study, the incidence of confirmed influenza was too low to convincingly demonstrate efficacy. However, in subjects receiving active vaccine (both dose regimens combined) vs. placebo, the incidence of culture confirmed influenza with respiratory symptoms was respectively 0.77% (7/904) vs. 2.03% (9/443), a relative risk reduction of 62% (p = 0.045). Incidence was essentially indistinguishable between dosing groups, 0.9% (4/455) and 0.7% (3/450) for the 15 μg x 2 and 30 μg x 1 groups respectively. The efficacy of the vaccine occurred despite the emergence of a drift variant, A/Fujian/411/02-like (H3N2), which accounted for 96.8% of H3N2 isolates in Canada that year. This strain was not well matched to the vaccine. The efficacy of mismatched vaccines has been estimated at 50% (95% CI: 27–65%) [21]. Thus the efficacy of the field study, although modest, is encouraging. This demonstrates the utility of the human viral challenge model in the design of subsequent field studies as previously reported [14].

The definition of influenza illness varied between the challenge and field studies; upper respiratory, lower respiratory or systemic illness plus seroconversion in the former; cough and at least one of sore throat, runny nose or nasal congestion, muscle or joint ache, headache, fatigue, or chills with symptoms sufficient to impede normal daily activities plus culture confirmed influenza (positive nose and throat swab for influenza A or B virus) in the latter study. Despite this, a comparison of equivalent dosing regimens (15 μg x 2 and 30 μg x 1) between the two studies shows a 67% efficacy against a matched challenge strain (non-weighted average for the two dosing groups) and a 62% efficacy against a mismatched field strain. The fact that the 30 μg x 1 dose performed more poorly than the 15 μg x 2 dose in the challenge study but not in the field study is unexplained although the very low rates of confirmed influenza illness in the field study make comparisons difficult.

The results of this previously mentioned field study add to the body of evidence that the human viral challenge model can provide proof of concept and thus be a good predictor of outcomes in subsequent clinical trials.

## Conclusion

Intranasal vaccination significantly protected against illness associated with evidence of A/Panama infection following challenge with A/Panama/2007/1999 (H3N2). Data from both studies were pooled and vaccine efficacy calculated by contrasting incidence rates observed in vaccine recipients with those observed in placebo recipients. Efficacy between active dosing groups ranged from 58% to 82% for any influenza symptoms and seroconversion, 67% to 85% for systemic or lower respiratory illness and seroconversion, and 65% to 100% for febrile illness and seroconversion. The two dose regimen was superior to the one dose regimen.

Correlates of protection against influenza are poorly understood, however in this study we confirmed that protection against illness associated with evidence of influenza infection (evidence determined by seroconversion) following challenge with virus was significantly correlated with the pre-challenge titres of serum HAI antibody (p = 0.0003) and mucosal sIgA (p≤0.0001) individually, and serum HAI titre (p = 0.028) and sIgA level (p = 0.0014) when both are considered together in the model.

## Supporting Information

S1 File IDB-13004.doc. The study protocol, for study entitled IDB-13004
(DOC)

S2 File IDB-13005.doc The study protocol, for study entitled IDB-13005
(DOC)

S3 File CONSORT 2010 Checklist.doc (DOC)
