# Supplementary material for: An Intranasal Proteosome-Adjuvanted Trivalent Influenza Vaccine Is Safe, Immunogenic & Efficacious in the Human Viral Influenza Challenge Model. Serum IgG & Mucosal IgA Are Important Correlates of Protection against Illness Associated with Infection
Source: PLoS One. 2016 Dec 22;11(12):e0163089. doi: 10.1371/journal.pone.0163089 (PMC5179046; doi:10.1371/journal.pone.0163089)
Supplement: S1 File — The study protocol, for study entitled IDB-13004 (DOC) [file pone.0163089.s001.doc]

STUDY TITLE: A Phase II, Randomized, Double-Blind, Placebo-Controlled Study of One- and Two-Dose Regimens of FluINsure™ Proteosome-Trivalent Influenza Vaccine Delivered by the Intranasal Route to Healthy Young Adults, Followed by Intranasal Challenge with Virulent Influenza A Virus

Protocol No.: IDB-13004

Sponsor: ID Biomedical of Québec

7150 Frederick Banting, Suite 200

Ville St-Laurent, QC, H4S 2A1, Canada

Reg. File No.: 19896 / 0001 / A 69186

Principal Investigator: Dr. Robert Lambkin, BSc (Hons), MRPharmS, PhD

Medical Investigator: Dr. Colin Gelder, BSc (Hons), MB (Hons), PhD, MRCP

Study Site: Retroscreen Virology Ltd.

The Medical Building

Queen Mary, University of London

327 Mile End Road, London, E1 4NS, U.K.

Version / Date: Version 6 / 25 Sep, 2002

Sponsor’s Study Director: Louis F. Fries, M.D.

Address as below

Clinical Monitor: Louis F. Fries, M.D.

ID Biomedical

UMBC Technology Center

1450 S. Rolling Rd., Baltimore, MD 21227, U.S.A.

(voice): 410-455-5610 (fax): 410-455-5606

(mobile): 443-253-5298

Do Not Implement This Protocol Unless Signed on the Following Page By The Study Director And All Investigators.

*This protocol is a confidential communication of ID Biomedical, and is prepared for the sole purpose of informing Investigators and their professional assistants who are participating in clinical trials of the Proteosome-Trivalent Influenza Vaccine*

Signature Page:

The Investigators and the Sponsor have discussed and agreed upon the content of this protocol. The Investigators agree to perform this investigation according to protocol and in conformance with cGCP, and to abide by this protocol except in the case of medical emergencies or where departures from the protocol are necessary in the interest of subject safety. In non-emergent situations, such departures will be undertaken only after consultation with the sponsor.

Principal Investigator: _______________________________________________

Dr. Robert Lambkin, BSc (Hons), MRPharmS, PhD Date

Medical Investigator: _______________________________________________

Dr. Colin Gelder, BSc (Hons), MB (Hons), PhD, MRCP Date

Co-Investigator : _______________________________________________

Professor John S. Oxford, BSc (Hons), PhD Date

Sponsor’s Study

Director: _______________________________________________

Louis F. Fries, M.D. Date

Contents:

[Study Synopsis 6](#__RefHeading___Toc21857851)

[List of Abbreviations 10](#__RefHeading___Toc21857852)

[1.0 Introduction and Background: 11](#__RefHeading___Toc21857853)

[2.0 Objectives: 16](#__RefHeading___Toc21857854)

[3.0 Study Administration: 16](#__RefHeading___Toc21857855)

[3.1 Ethical Review and Informed Consent: 16](#__RefHeading___Toc21857856)

[3.2 Record Keeping, Monitoring, and Record Retention: 16](#__RefHeading___Toc21857857)

[4.0 Study Population: 17](#__RefHeading___Toc21857858)

[4.1 Subject Number: 17](#__RefHeading___Toc21857859)

[4.2 Inclusion Criteria: 17](#__RefHeading___Toc21857860)

[4.3 Exclusion Criteria: 17](#__RefHeading___Toc21857861)

[5.0 Study Design: 18](#__RefHeading___Toc21857862)

[5.1 Immunization Component: 19](#__RefHeading___Toc21857863)

[5.1.1 Treatments: 19](#__RefHeading___Toc21857864)

[5.1.2 Safety and Tolerability Evaluation: 20](#__RefHeading___Toc21857865)

[5.1.3 Immunogenicity Evaluation: 20](#__RefHeading___Toc21857866)

[5.2 Challenge Component: 20](#__RefHeading___Toc21857867)

[6.0 Investigational Supplies: 21](#__RefHeading___Toc21857868)

[6.1 Study Drugs: 21](#__RefHeading___Toc21857869)

[6.2 Delivery System for Study Drugs: 22](#__RefHeading___Toc21857870)

[6.3 Storage Requirements for Study Drugs: 22](#__RefHeading___Toc21857871)

[6.4 Test Article Dose Preparation: 22](#__RefHeading___Toc21857872)

[6.4.1 Randomization / Study Day 0: 22](#__RefHeading___Toc21857873)

[6.4.2 Preparation of Individual Test Article Doses: 23](#__RefHeading___Toc21857874)

[6.5 Test Article Dose Administration: 23](#__RefHeading___Toc21857875)

[6.6 Accountability for Study Drugs: 24](#__RefHeading___Toc21857877)

[6.7 Challenge Virus: 24](#__RefHeading___Toc21857878)

[6.8 Storage of Challenge Virus: 24](#__RefHeading___Toc21857879)

[6.9 Disposal of Challenge Virus: 24](#__RefHeading___Toc21857880)

[7.0 Procedures and Methods: 25](#__RefHeading___Toc21857881)

[7.1 Procedures: 25](#__RefHeading___Toc21857882)

[7.1.1 First Screening Visit (days - 90 to - 7): 25](#__RefHeading___Toc21857883)

[7.1.2 Second Screening Visit (days -42 to - 5): 25](#__RefHeading___Toc21857884)

[7.1.3 Final Screening Visit (days -14 to – 2): 25](#__RefHeading___Toc21857885)

[7.1.4 Day 0, First Treatment: 26](#__RefHeading___Toc21857886)

[7.1.5 Day 2 (day 3 acceptable): 27](#__RefHeading___Toc21857887)

[7.1.6 Day 7 (± 1 day) Telephone Follow-up: 27](#__RefHeading___Toc21857888)

[7.1.7 Day 12 (± 1 day): 27](#__RefHeading___Toc21857889)

[7.1.8 Day 14 (± 2 days, but not within 24 hours of the Day 12 visit): 28](#__RefHeading___Toc21857890)

[7.1.9 Day 16 (day 17 acceptable): 28](#__RefHeading___Toc21857891)

[7.1.10 Day 21 (± 1 day) Telephone Follow-Up: 29](#__RefHeading___Toc21857892)

[7.1.11 Day 28 (± 3 days): 29](#__RefHeading___Toc21857893)

[7.1.12 Day 40 (± 3 days), Admission for Challenge: 29](#__RefHeading___Toc21857894)

[7.1.13 Day 40 Post-admission to Day 41: 30](#__RefHeading___Toc21857895)

[7.1.14 Day 42 a.m. (Day of Challenge): 30](#__RefHeading___Toc21857896)

[7.1.15 Day 42 p.m. to Day 49: 31](#__RefHeading___Toc21857897)

[7.1.16 Day 49: 32](#__RefHeading___Toc21857898)

[7.1.17 Day 60 (± 3 days): 32](#__RefHeading___Toc21857899)

[7.2 Laboratory Tests: 33](#__RefHeading___Toc21857900)

[7.2.1 Hematology: 33](#__RefHeading___Toc21857901)

[7.2.2 Clinical Chemistry: 33](#__RefHeading___Toc21857902)

[7.2.3 Clinical Laboratory Serologies: 33](#__RefHeading___Toc21857903)

[7.2.4 Urinalysis: 33](#__RefHeading___Toc21857904)

[7.2.5 Pregnancy Testing: 33](#__RefHeading___Toc21857905)

[7.2.6 Specific Antibody Assays: 34](#__RefHeading___Toc21857906)

[7.2.7 Studies of Cytokine Induction : 34](#__RefHeading___Toc21857907)

[7.2.8 Influenza Virus Detection: 34](#__RefHeading___Toc21857908)

[7.3 Other Measurements: 34](#__RefHeading___Toc21857909)

[7.3.1 History and Physical Examination: 34](#__RefHeading___Toc21857910)

[7.3.2 Brief, Directed Physical Examination after Treatments: 35](#__RefHeading___Toc21857911)

[7.3.3 Directed Physical Examination During Challenge: 35](#__RefHeading___Toc21857912)

[7.3.4 Concomitant Medications: 35](#__RefHeading___Toc21857913)

[7.4 Early Discontinuation: 35](#__RefHeading___Toc21857914)

[8.0 Immediate Complaints, Vaccine Reactogenicity, Findings of Influenza and Adverse Events: 36](#__RefHeading___Toc21857915)

[8.1 Immediate Complaints: 36](#__RefHeading___Toc21857916)

[8.2 Vaccine Reactogenicity: 36](#__RefHeading___Toc21857917)

[8.3 Symptoms and Findings of Influenza: 36](#__RefHeading___Toc21857918)

[8.4 Adverse Events: 37](#__RefHeading___Toc21857919)

[8.4.1 Double Reporting of Reactogenicity Findings/Complaints and Findings of Influenza as Adverse Events: 37](#__RefHeading___Toc21857920)

[8.5 Serious Adverse Events: 39](#__RefHeading___Toc21857921)

[9.0 Analysis Plan: 39](#__RefHeading___Toc21857922)

[9.1 Immunogenicity: 40](#__RefHeading___Toc21857923)

[9.2 Safety: 41](#__RefHeading___Toc21857924)

[9.2.1 Power and Detectable Effect Size: 41](#__RefHeading___Toc21857925)

[9.2.2 Immediate Complaints, Vaccine Reactogenicity Complaints, and Standardized Ear, Nose, and Throat Exams: 42](#__RefHeading___Toc21857926)

[9.2.3 Adverse Events: 42](#__RefHeading___Toc21857927)

[9.2.4 Vital Signs and Clinical Laboratory Measures: 43](#__RefHeading___Toc21857928)

[9.3 Illness Definitions and Analysis for Challenge Component: 43](#__RefHeading___Toc21857929)

[9.3.1 Fever: 43](#__RefHeading___Toc21857930)

[9.3.2 Upper Respiratory Illness: 43](#__RefHeading___Toc21857931)

[9.3.3 Lower Respiratory Illness: 43](#__RefHeading___Toc21857932)

[9.3.4 Systemic Illness: 44](#__RefHeading___Toc21857933)

[9.3.5 Illness (any): 44](#__RefHeading___Toc21857934)

[9.3.6 Illness Score: 44](#__RefHeading___Toc21857935)

[9.3.7 Infection: 44](#__RefHeading___Toc21857936)

[9.3.8 Analysis: 45](#__RefHeading___Toc21857937)

[9.3.8.1 Power Considerations: 45](#__RefHeading___Toc21857938)

[9.3.8.2 Analyses: 45](#__RefHeading___Toc21857939)

[10.0 References: 46](#__RefHeading___Toc21857940)

[Appendix A. Proteosome-Trivalent Influenza](#__RefHeading___Toc21857941) [Vaccine Dose Preparation Worksheet 50](#__RefHeading___Toc21857942)

[Appendix B. Table B.1 52](#__RefHeading___Toc21857943)

[Appendix B. Table B.2 53](#__RefHeading___Toc21857944)

[Appendix C. Brief Examination Worksheet (Post-Immunization) 54](#__RefHeading___Toc21857945)

[Appendix D. Prototype Memory Aid Page 55](#__RefHeading___Toc21857947)

[Appendix E. Immediate Complaints Questionnaire 56](#__RefHeading___Toc21857949)

[Appendix F. Influenza Symptom Diary card 57](#__RefHeading___Toc21857950)

[Appendix G. Challenge Physical Examination Worksheet 58](#__RefHeading___Toc21857951)

# Study Synopsis

| Company: | ID Biomedical of Québec |
| --- | --- |
| Trial Number: | IDB - 13004 |
| Finished Product: | FluInsure™ Proteosome – Trivalent Influenza Vaccine |
| Active Ingredient(s): | Hemagglutinin (HA) from each of A/New Caledonia / 20 /99 (H1N1), A/Panama/2007/99 (H3N2), and B/Victoria/504/2000 viruses and meningococcal outer membrane proteins |
| Title of the Trial: | A Phase II Randomized, Double-blind, Placebo-Controlled Study of One- and Two-Dose Regimens of FluINsure™ Proteosome-Trivalent Influenza Vaccine Delivered by the Intranasal Route to Healthy Young Adults, Followed by Intranasal Challenge with Virulent Influenza A Virus |
| Development Phase: | Phase II |
| Principal Investigator: | Dr. Robert Lambkin, BSc (Hons), MRPharmS, PhD |
| Co-Investigator: | Professor John Oxford, BSc (Hons), PhD |
| Medical Investigator: | Dr. Colin Gelder, BSc (Hons), MB (Hons), PhD, MRCP |
| Trial Centers: | Retroscreen Virology Ltd., London |
| Planned Trial Period: | Two months to complete screening and enrollment, treatment and detailed safety assessment; approximately eight (8) days of inpatient confinement for challenge, final follow-up 14 days post discharge. |
| Objectives: | 1. To evaluate safety and tolerability of FluInsure™ when delivered intranasally in two different regimens to healthy adults, 18 – 50 y.o., selected for pre-existing susceptible immune status with regard to A/Panama//2007/99  2. To evaluate the magnitude of immune responses, in the serum and mucosal compartments, to FluInsure™ given in two different regimens.  3. To develop preliminary data regarding the protective efficacy of FluInsure™ by evaluating reduction in influenza-like illness and viral shedding following intranasal challenge with virulent A/Panama/2007/99 |
| Trial Design: | Immunization: Randomized, blinded, and placebo-controlled; featuring the following treatment groups:   1. 30 μg of each of the three HAs, IN x 1, and buffered saline placebo IN x 1, (N = 25) 2. 30 μg of each of the three HAs, IN x 2 (N = 25) 3. Buffered saline placebo, IN x 2 (N = 25)   Challenge: Intranasal challenge of approximately 20 subjects randomly drawn from each of the above three treatment groups at 42 days after the first test article dose. Challenge will be administered by nasal drops containing viable, egg-grown A/Panama/2007/99 |
| Planned Sample Size: | 75 healthy adult volunteers treated, 60 – 75 challenged |
| Inclusion Criteria: | 1. Age 18 to 50 years 2. Good general health status as determined by screening evaluation no greater than 42 days prior to immunization 3. Comprehension of the study requirements, including willingness to forego the licensed 2002 – 2003 intramuscular influenza vaccine until protocol participation is complete; expressed availability to fulfill the study requirements; signed informed consent 4. For female subjects, provision of a history of reliable contraceptive practices 5. Serum reciprocal hemagglutination inhibition titer for A/Panama/2007/99 of ≤10 at screening prior to the immunization phase |

| Exclusion Criteria: | 1. Presence of significant acute or chronic, uncontrolled medical or psychiatric illness; significant abnormalities in baseline serum chemistry, hematology, or urinalysis parameters 2. Positive serologic test for HIV 1 or 2 3. In female subjects, a positive urine β-HCG on the day of any test article dose or virus challenge 4. Chronic use of any medication or other product, prescription or over-the-counter, for symptoms of rhinitis or nasal congestion, or any chronic nasopharyngeal complaint 5. Any history of asthma of any etiology in adulthood 6. Smokers unwilling/unable to desist for the inpatient component of the trial 7. Acute use of any medication or other product, prescription or over-the-counter, for symptoms of rhinitis or nasal congestion within seven (7) days prior to the first test article dose 8. Abnormal ECG 9. Any anatomic or neurologic abnormality impairing the gag reflex or conducive to aspiration, or history suggestive of such a problem 10. Receipt of systemic glucocorticoids (in a dose ≥ 5 mg prednisone daily or equivalent) within one month, or any other cytotoxic or immunosuppressive drug within six months 11. Receipt of any investigational drug within one month, or participation in a clinical trial of any influenza vaccine or any influenza challenge within one year 12. Presence of any febrile illness or significant symptoms of upper respiratory infection on the day of immunization or between admission for influenza challenge and administration of the challenge inoculum 13. History of hypersensitivity to mercurials or chicken eggs |
| --- | --- |
| Investigational Product: | FluInsure™ Proteosome Trivalent Influenza Vaccine:  Form: liquid (0.9 mL per 3 mL vial)  Dose: 30 μg of each of three (3) influenza hemagglutinins complexed to proteosomes; total volume administered = 0.28 mL by nasal spray  Each vial contains:   1. 150 ± 38 μg / mL of hemagglutinin (HA) from *each* of A/New Caledonia/20/99 (H1N1), A/Panama/2007/99 (H3N2) and B/Victoria/504/2000 viruses, non-covalently bound to outer membrane proteins (OMP) of *Neisseria meningitidis* strain 8047 (OMP to HA ratio is approximately 4:1). 2. 10 mM Na/K phosphate-buffered isotonic saline, pH 7.4, with 0.01% thimerosal 3. Non-hemagglutinin viral proteins 4. Trace amounts of egg proteins |
| Control Product: | Phosphate buffered normal saline diluent / placebo:  Form: liquid (10 mL per 20 mL vial)  Dose volume: 0.28 mL by nasal spray  Each vial contains:  10 mM Na/K phosphate-buffered isotonic saline, pH 7.4, with 0.01% thimerosal |
| Challenge: | A/Panama/2007/99, lot I B 44/3, prepared in eggs in compliance with GMP, sterile and mycoplasma-free |
| Trial Time Lines: | Immunization in November 2002. Challenge January 2003. |
| Study Schedule | Test article administrations will occur on days 0 and 14 (± 1). Clinical evaluations will be performed at screening, before and after each test article dose, and at days 28 (± 2) and 40. Specimens for clinical chemistry, hematology and urinalysis will be collected at screening (prior to day 0) and on day 28 (± 2). Specimens for assessment of immune response will be collected at screening, before each test article dose, and on days 28 (± 2) and 40.  Subjects will be admitted to an inpatient challenge facility on day 40 and be observed for signs of spontaneous illness for 48 hours. On day 42, the intranasal challenge will be administered. Subjects will undergo twice-daily clinical evaluation for influenza-like illness from the evening of day 42 through day 49. In addition, daily throat swabs and nasal washes will be performed to monitor virus shedding. On day 48, subjects will begin treatment with a neuraminidase inhibitor to ensure virus clearance prior to discharge. A follow-up visit on day 60 will include a clinical evaluation and serum sampling for antibody responses to the challenge agent.  A detailed study schedule appears in appendix B. |

| Follow-up Duration: | Volunteers will be followed for 60 days after the day 0 immunization. |
| --- | --- |
| Assessment Methods: | 1. Standard clinical parameters for evaluating the safety of a biologic or vaccine product including: standardized diaries/questionnaires for local and systemic vaccine reactions, repeated vital signs and physical examinations, 60-day follow-up for adverse events and concomitant medication changes, monitoring of clinical chemistries (liver and renal function), urinalysis, and hematology.   2. Immunogenicity assessments will include serum hemagglutinin-inhibiting (HAI) antibody specific for the three viruses included in the vaccine, and levels of secretory IgA for these viruses present in nasal wash fluids.  3. Evaluation of challenge induced influenza will include repeated vital signs and examinations of the ears, nose, throat and lungs, and tympanometry. In addition, a standardized symptom questionnaire will be administered twice daily. Once-daily nasal washes and throat swabs will be performed for quantitative culture of influenza virus. |
| Statistical Methods:  Statistical Methods (cont’d.) | Demography: descriptive statistics will be provided.  Vaccine reactogenicity: Analyses concern graded severities for local and systemic reactogenicity: Comparing one group with another during a treatment period (the design has two periods following the first and second test article exposures), these are two-sample, cross-sectional comparisons of binary, ordinal, or continuous data. Comparing a group with itself between periods, these are paired, repeated measures data. Two-sample, cross-sectional comparisons are based on Fisher’s exact and Cochran-Mantel-Haenszel tests for binary and ordinal outcomes, respectively. Student’s-t and Wilcoxon tests are used for continuous outcomes. These methods permit statistical tests to address the following topics:   - 1. comparison of active and placebo after test article dose 1   2. comparison of active and placebo after test article dose 2, with and without testing for the impact of, and adjustment for, outcomes after dose 1   3. comparison of active and placebo in successive test article doses   4. comparison of placebo in successive test article doses   5. comparison of change from active to active with change from placebo to placebo   Items c. and d. are paired comparisons, which may be performed by McNemar tests or, more flexibly, by GEE models with a binary or continuous outcome. Items b. and e. are tests of interaction: dose 1 by dose 2 in item b., and period by vaccine in item e. and also accessible by GEE models.  Adverse events: Adverse events will be tabulated by body system using the COSTART dictionary, by severity, by seriousness, by relationship to study drug, and by elapsed time since last exposure to study drug. This yields primarily binary data (for each subject, the given COSTART event did or did not occur) which may be analyzed in the same manner as the binary reactogenicity events.  Vital signs & clinical laboratory measures: Mean values and S.D.s will be plotted separately by treatment group. Extreme values and/or outliers will be discussed individually. For clinical laboratory measures within cross-over groups, quantitative outcomes at baseline and day 28 will be compared by paired Student’s t-tests and Wilcoxon signed rank tests. Binary outcomes will be compared by McNemar’s tests.  Immunogenicity: Immunogenicity measures will include serum hemagglutination-inhibiting (HAI) antibody titers specific for the strains included in the vaccine, levels of secretory IgA (sIgA) specific for these viruses in nasal wash fluids. For the HAI titers, analyses will concern geometric mean titers (GMT), proportions of subjects with titer ≥ 40, and proportions with ≥ four-fold increase over baseline. For specific sIgA levels, analyses will concern geometric means and fold-rise from baseline. HAI GMTs and geometric mean nasal specific sIgA levels will be accompanied by 95% confidence intervals. Comparing active-to-placebo and active-to-active groups, titers/antibody levels will be contrasted on day 28 by t-tests on log_10_(titer). Within the groups, paired tests will be used (Student’s t and Wilcoxon signed rank). Fold-rises may be examined with and without covariate-adjustment for previous titers. Without such adjustment, there are two binary fold-rise outcomes, and these are compared within and between groups by chi-square and stratified chi-square tests. (In the latter case, 2 x 2 tables of treatment by outcome are stratified by previous titers.) With covariate adjustment, the fold-rise outcome is the log_10_-ratio of early and later titers. Adjustment after the first treatment is for pre-treatment titer and adjustment after the second treatment is for both baseline titer and for titers after the first treatment.  Response to viral challenge: The analysis concerns between-group comparisons of the three groups regarding clinical and immunologic binary outcomes. Alternative definitions provide six binary clinical outcomes that can be compared between groups by chi-square tests. More flexible analyses use logistic regression (with the presence or absence of illness, separately for each definition, as the outcome. Covariates are cross-over study group, age, gender, race, and, for some models, titers after vaccinations 1 and 2.  Additional analyses will be based on illness score and on virus shedding assessed by two methods: peak and a time-weighted average calculated as a trapezoidal-rule AUC. For both peak and AUC, viral shedding is in units of log_10_(titer). These continuous data will be compared between cross-over groups by Student’s t-tests and Wilcoxon tests and, in a more flexible analysis, by linear model adjusted for the demographic and clinical covariates cited above.  Finally, the three binary measures of influenza and the two viral load estimates (peak titer and AUC) will be assessed relative to the two immunologic measures, HAI reciprocal titer and nasal secretory IgA. For the binary measures, mean viral load (for each measure) is compared by t-tests between those with and without influenza (for each definition). For the two immunologic measures, the test of association with viral load will be based on Pearson correlations of log-titers. |

# List of Abbreviations

AE Adverse event

ALT Serum alanine amino transferase

AST Serum aspartate amino transferase

β- HCG *Beta* – human chorionic gonadotropin

COSTART Coding Symbols For Thesaurus Of Adverse Reaction Terms

CRF Case report form

ECG Electrocardiogram

ELISA Enzyme linked Immunosorbent Assay

ERC Ethical review committee

GCP Good Clinical Practice

HA (Influenza) Hemagglutinin antigen

HAI (Influenza) Hemagglutination Inhibiting (antibody)

HIV Human Immunodeficiency Virus

ICH International Conference on Harmonization

IgG Immunoglobulin G

Na / K Sodium / Potassium

LD_50_ 50% Lethal dose

OMP Outer Membrane Protein

RR Relative risk

SAE Serious Adverse Event

sIgA Secretory Immunoglobulin A

SID Subject Identification Number

1.0 Introduction and Background:

Influenza remains a major cause of morbidity and mortality in most areas of the world (1). Attack rates are highest in young children (especially infants 6 to 12 months of age [2]), but hospitalization and death are highest in the elderly and in patients with underlying metabolic, cardiovascular and pulmonary diseases. The average seasonal incidence of influenza-related death was reported to be 9.1 per 100,000 for the 1972-1992 period in the United States. In non-pandemic years, over 90% of deaths occur among persons 65 years of age or older (3). Although only a small proportion of influenza-related death occurs in the pediatric population, children have the highest morbidity rates and are also the major disseminators of the virus. Effective vaccination coverage of school-age children has resulted in a three-fold reduction in the overall rate of illness in all age groups (4).

Beneficial effects of the current inactivated, trivalent vaccines are most evident in healthy, young adults. A recent meta-analysis suggested that the frequently quoted estimate of 70% efficacy against serologically-confirmed influenza illness is borne out by the literature pertaining to the young adult age group (5). The virus-specific serum antibodies that are elicited in this group are pivotal elements in limiting or aborting spread of the virus and preventing illness, but are relatively ineffective at preventing infection *per se* or shortening viral shedding (1, 6, 7). In children, the current vaccine is immunogenic if two doses are given, but the requirement for two parenteral doses, repeated yearly, inhibits acceptance. In the elderly, more modest protection is achievable with the current vaccines, perhaps on the order of 40-60% (8, 9). This relative failure of protection in the elderly has been attributed to various factors, including lack of stimulation of a mucosal immune response (see below), and the senescence of T and B-cells and reduced IL-2 production (3, 10).

During natural infection influenza viruses gain entry through, and are essentially restricted to, respiratory mucosal surfaces. Consequently, mucosal immunity is a pivotal element in controlling these respiratory viral infections. The mucosal immune system consists of an integrated network of lymphoid cells working in concert with the innate mucosal barriers to promote host defense. The humoral arm of the mucosal immune system is comprises principally locally-synthesized polymeric IgA antibodies. Secretory IgA (sIgA) constitutes the majority (>80%) of all antibodies produced in mucosae-associated lymphoid tissues in humans and forms a first line of immune defense. The induction of sIgA antibodies has a key role in the *prevention* of mucosae-restricted respiratory infections. Secretory IgA functions in host defense have been demonstrated at three levels: a) in the lamina propria IgA antibodies bind antigens and cause them to be cleared into the lumen (11); b) antiviral IgA antibodies in transit through epithelial cells inhibit virus production by an intracellular action (12); and c) IgA antibodies secreted into the lumen can prevent antigens from adhering to and penetrating the epithelium (13). The mucosal immune response, as monitored by specific sIgA in the nose, has been shown to be a strong correlate of reduced infection rate, limited viral replication, and reduced illness in influenza challenge studies in adults (1, 6).

Although influenza immunization rates are increasing, the desired impact on morbidity and mortality has not been obtained. In order to address pediatric and elderly populations, mucosal immunization strategies, of which the most intensively investigated has been nasal immunization with live, cold-adapted reassortant viruses, have been developed. Assessment of the immunogenicity and efficacy of cold-adapted influenza vaccines have demonstrated that they can induce mucosal immune responses and show protective efficacy in children and in healthy young adult volunteers (5, 14 – 17). In naïve children, immune responses to these vaccines are vigorous in both the serum and mucosal compartments, whereas in seropositive children, and even sero-negative adults, serum responses are modest. In studies performed in the elderly, cold-adapted influenza vaccines have offered minimal or no advantage over inactivated virus vaccines in terms of serum or secretory antibody or local immunological memory induction in this group (18). A recent meta-analysis concluded that the efficacies of inactivated intramuscular and live, cold-adapted intranasal vaccines were similar, but included predominantly young adult data (19).

An alternative approach to intranasal influenza immunization is the use of inactivated vaccine antigens via the nose. Inactivated influenza antigens of various types, when given intranasally, have been safe in a large cumulative number of adults and children. They have induced significant mucosal and systemic immune responses, but crude, unfractionated virus and/or potentially uneconomic doses of more purified antigens were frequently required (20 - 28). Limited data suggest that inactivated influenza antigens given intranasally can have protective efficacy comparable to intramuscular vaccine (reviewed in 5, see also 20, 21, 23, 24). Lipid-based delivery systems have been reported to enhance influenza antigen immunogenicity via the nasal route (29). Native *E. coli* heat-labile toxin has also been shown to have potent nasal adjuvant properties for influenza antigens (30). However, this adjuvant has been shown to enter neural tissues and cause inflammatory pathology when given intranasally to rodents (31) – and may be associated with unacceptable safety concerns, such as facial nerve palsies, in humans.

Proteosomes are hydrophobic, proteinaceous nanoparticles comprising *Neisseria meningitidis* outer membrane proteins. Proteosomes can non-covalently associate with macromolecules containing hydrophobic domains and successfully present these macromolecules to the mucosal immune system (32). The proteosome mucosal vaccine delivery system is one of the limited number of

sub-unit approaches capable of eliciting mucosal and systemic responses *in vivo.* It is well-suited to the delivery of amphophilic viral membrane glycoproteins such as the influenza hemagglutinin and neuraminidase. Animal studies of mucosally-applied proteosome-formulated vaccines have demonstrated strong systemic and mucosal antigen-specific immune responses and protection against disease in several model systems using multiple classes of antigen, including influenza (32 - 34). Recently, intranasal administration to adult humans of proteosome-based vaccines against influenza or bacillary dysentery, the latter in doses containing up to 1.5 mg of proteosome protein, has proven safe, well-tolerated and immunogenic in both the systemic and mucosal compartments (35, and ID Biomedical unpublished data).

Murine studies of intranasally-delivered proteosome-influenza vaccines have shown induction of virus-specific serum IgG, hemagglutination-inhibiting, and virus-neutralizing antibodies in titers equivalent to those induced by classical inactivated intramuscular vaccine antigens given in the same dose, or by sub-lethal infection with homologous virus. Induction of statistically-significant virus-specific serum IgA titers (which in mice reflect mucosal production), nasal and pulmonary IgA titers was also observed. Neutralizing antibodies were observed in lung washes of mice that received intranasal proteosome-influenza vaccine, but not those of mice that received intramuscular vaccine. Mice immunized intranasally with proteosome-influenza vaccine showed solid protection against an intranasal challenge with 4 LD_50_ of homologous mouse-adapted virus. (36)

In humans, ID Biomedical initially carried out a phase I dose-escalating trial of a prototoype proteosome-A/Beijing/262/95 monovalent vaccine in 54 healthy young adults sero-negative for A/Beijing/262/95 by serum HAI titers (37). The vaccine used a U.S.-licensed, detergent-split, ether-extracted, monovalent A/Beijing antigen. Vaccinees received two intranasal doses containing 7.5, 15, or 30 μg of A/Beijing/262/95 antigen (measured as HA), formulated with a four (4)-fold weight excess of proteosomes, at a 14 day interval. Control groups received a commercial vaccine containing A/Beijing/262/95 antigen given intramuscularly, or, alternatively, A/Beijing antigen without proteosomes given intranasally. Dose-escalation proceeded without difficulty. Immediate complaints after vaccine administration were minimal. Repeated ear, nose, and throat examinations at two (2) and seven (7) days after each dose revealed an association of the intranasal proteosome-influenza vaccine with mild, transient nasal mucosal erythema (up to 46% of subjects after one or both doses) and occasional scant, clear rhinorrhea (up to 38% of subjects after one or both doses). Subjects maintained a symptom diary for seven (7) days after each dose. Systemic symptoms (malaise, headache, etc.) occurred with similar frequency in all groups. Rhinorrhea and nasal congestion were clearly associated with the intranasal proteosome-influenza vaccine, but were almost exclusively mild and short-lived.

At doses containing 15 or 30 μg of A/Beijing/262/95 antigen, 46-54% of proteosome-A/Beijing vaccinees had ≥ 4-fold rises in serum HAI antibodies; the majority of these attaining titers ≥ 40, which are historically associated with protection. Fifty-four (54) % of proteosome-A/Beijing intranasal vaccine recipients in these same groups had statistically-significant (>2.75-fold) rises in nasal wash sIgA specific for A/Beijing262/95. While HAI responses were strongly dose-responsive, nasal wash specific sIgA responses were not; hence the optimal intranasal dose was not well defined. Further, immune response kinetics did not clearly demonstrate the benefit of a second dose at day 14 versus a single larger dose initially.

ID Biomedical then carried out an expanded phase I study of the same monovalent prototype (38). In this 100-subject trial, dose regimens providing either 15 or 30 μg of A/Beijing/262/95 antigen given intranasally twice at a 14-day interval were compared to single-dose regimens providing 30 or 45 μg of A/Beijing/262/95 antigen in a population unselected for baseline immunity to influenza. Subjects in groups receiving single active vaccine dose also received a dose of saline placebo, in a blinded fashion and in random order, at a 14-day interval. These subjects permitted a crossover analysis of reactogenicity within the same subject. Safety and immunogenicity analyses showed that there was no apparent qualitative or quantitative difference in the tolerability of single doses containing up to 45 μg of influenza antigen, and no significant difference in the safety profile of any dosing regimen. Rhinorrhea and nasal congestion in the seven days post-dosing were associated with the active vaccine (p < 0.1 versus placebo in the crossover analysis), but these were exclusively graded as mild and had median durations of ≤ two (2) days. No subject became febrile, and repeated physical examinations of the nasopharynx after dosing were benign. Immunogenicity in subjects with pre-existing serum HAI titers of ≥ 40 (and presumptively already immune) was modest, but strong serum and nasal antibody responses similar to the data cited previously were observed in susceptible subjects with baseline serum HAI titers < 40. Approximately 68% of all subjects in the two-dose treatment groups had statistically-signficant increases in specific nasal wash sIgA levels, rising to 90% when only susceptible subjects were considered. The corresponding proportions in one-dose treatment groups were 51 and 58%. There was little influence of dose level. Overall, 78% of previously non-immune subjects manifested a potentially-protective response in serum, nasal fluid, or both. The immunogenicity data showed a relatively shallow dose-response. No clear advantage of two-dose over one-dose regimens was shown with regard to serum HAI responses. Although all vaccine groups had specific sIgA responses in nasal fluids, two-dose groups appeared to have larger and more durable increases.

A phase I trial (which is nearing completion) and two phase II trials of trivalent proteosome-influenza vaccine formulations are currently ongoing in Canada. The vaccines in these trials include influenza antigens prepared by manufacturers serving the E.U. market. These trials are placebo-controlled, and are evaluating a range of dose regimens, including single doses containing 15, 30, or 45 μg of each hemagglutinin and two-dose regimens delivering 15 or 30 μg of each hemagglutinin twice, at a 14-day interval. Initial safety data appear essentially identical to the safety results of the monovalent trials described above, including data from subjects receiving doses containing up to 45 μg of *each* of the three hemagglutinins. The only signficant reactogenicity of the trivalent products appears to be mild and transient rhinorrhea and nasal congestion. Initial serum HAI data indicate that, for each of the three virus strains in the trivalent preparation, immunogenicity is as good as, or superior to, the performance seen in the monovalent trials. Nasal wash sIgA data demonstrate the induction of statistically significant increases in virus-specific nasal fluid antibody to each of the three virus strains. Increases in nasal wash specific sIgA levels range from two (2)- to four(4)-fold, and are equal to, or greater than, published responses to live, cold-adapted vaccine viruses in the young adult study population (16).

In summary, ID Biomedical has developed data in over 200 healthy adults indicating that monovalent and trivalent intranasal proteosome-influenza vaccines, prepared using egg-grown influenza antigens from three different commercial sources, are immunogenic in both the serum and mucosal compartments. They are also well-tolerated. Vaccine reactions appear to consist primarily of mild and self-limited clear rhinorrhea and nasal congestion, neither of which significantly impairs normal daily activities. Additional detail may be found in the Investigators’ Brochure.

As noted by Beyer *et al*., both increased serum HAI titers and specific nasal wash secretory IgA have been associated with protection against influenza (19). The relative importance of these factors in the induction of protection by non-living mucosal vaccines remains to be fully defined, rendering protective efficacy difficult to predict from immunogenicity data alone. Intranasal challenge of healthy young adults with egg-grown virulent influenza virus has been used extensively and safely in the United States and the United Kingdom to evaluate the immunogenicity and protective potential of a variety of live single-gene reassortant and subunit vaccine candidates (39, 40), to characterize the efficacy of the live, cold-adapted vaccine candidate and compare it to the intramuscular vaccine (41 – 43), and to test anti-viral compounds (44, 45). The current protocol proposes to study the capacity of a proteosome-trivalent influenza vaccine (FluINsure™) given intranasally as a one- or two-dose regimen, to prevent influenza-like illness and modulate influenza virus infection in healthy young adults challenged with A/Panama/2007/99 (H3N2).

# 2.0 Objectives:

1. To assess the safety and tolerability of FluINsure™ when delivered intranasally to healthy adults 18 to 50 y.o. who are selected for susceptibility to A/Panama/2007/99. Regimens delivering one or two doses containing 30 of each of the three hemagglutinin (HA) antigens will be compared to a placebo treatment.

B. To assess the kinetics and magnitude of the immune responses to the above intranasal FluINsure™ regimens in healthy adults 18 to 50 y.o. who are selected for susceptibility to A/Panama/2007/99.

C. To evaluate the capacity of the one- and two-dose regimens of FluINsure™ to provide protective immunity to previously-susceptible healthy young adults challenged with A/Panama/2007/99.

# 3.0 Study Administration:

## 3.1 Ethical Review and Informed Consent:

The clinical protocol will be conducted in conformance with the principles of the Declaration of Helsinki (Edinburgh, 2000), the International Ethical Guidelines for Biomedical Research Involving Human Patients, the ICH Consolidated Guideline for Good Clinical Practice, and all relevant local laws and regulations. The protocol and informed consent document will be reviewed and approved by a properly-constituted ethics committee and evidence of approval must be provided to ID Biomedical in writing prior to initiation of the trial. All aspects of the study will be explained in detail to prospective subjects and they will be informed of the voluntary nature of their participation. Written informed consent will be obtained from each subject using the approved documents, and each subject will receive a copy of his/her signed consents. Consent to screening will be obtained from each subject prior to obtaining an initial blood specimen for the sole purpose of determining sero-eligibility. Consent for participation in the immunization and challenge study will be obtained from sero-eligible subjects prior to any other study-related evaluations, procedures, or treatments.

## 3.2 Record Keeping, Monitoring, and Record Retention:

All required subject data will be recorded on case report forms (CRFs) or other study-specific media designed and provided by ID Biomedical. CRFs will be completed in a timely manner and be kept available, along with supporting source documents, for periodic monitoring by an ID Biomedical representative. The Investigator is responsible for the accuracy of the data recorded on the CRFs. One copy of all CRFs and all documentation surrounding the trial must be securely retained by the Investigator for twenty-five (25) years.

# 4.0 Study Population:

## 4.1 Subject Number:

Seventy-five healthy adults will be enrolled in the immunization portion of the study, i.e., 25 in each of three (3) treatment groups. Sixty of these will be randomly selected to participate in the challenge portion of the study (i.e., approximately 20 per treatment group).

## 4.2 Inclusion Criteria:

To be eligible for study, each subject will fulfill all of the following criteria:

• Age 18 to 50 years, inclusive,

• Comprehension of the study requirements, including willingness to forego the licensed 2002-2003 intramuscular influenza vaccine until completion of study participation; availability for the required study period, ability to attend scheduled study visits, and willingness to participate in the inpatient challenge,

- Willingness to provide written consent for participation after reading the Consent Form and after having adequate opportunity to discuss the study with an investigator or qualified deputy,
- Good general health status as determined by a screening evaluation no greater than 42 days prior to the first immunization,
- For female subjects, provision of a history of reliable contraceptive practices (hysterectomy or bilateral tubal ligation, oral or implanted contraceptive use, intrauterine device, barrier method plus spermicide, history of a single male partner with vasectomy, or a history of abstinence deemed credible by the investigator). *The provision of this history does NOT replace the requirement to perform, and obtain negative results in, pregnancy tests as per section 4.2.*
- Reciprocal titer of serum hemagglutination-inhibiting (HAI) antibody to A/Panama/2007/99 (H3N2) ≤ 10.

## 4.3 Exclusion Criteria:

Subjects with any of the following will be excluded prior to enrollment in the immunization component of the study:

- Presence of significant acute or chronic, uncontrolled medical or psychiatric illness (subjects with uncomplicated chronic diagnoses stable and treated for ≥ three [3] months, e.g., mild hypertension well-controlled with medication, may be enrolled - provided the condition and its therapy are not known to be associated with an immunocompromised state or increased risk of complications of influenza),

**•** Positive serologic test for HIV 1&2,

- ALT or AST > twice the upper limit of normal; or any clinical laboratory value deemed by the investigator to indicate significant undiagnosed illness,
- Abnormal ECG,

• In female subjects, a positive urine β-HCG on the day of any test article dose, or on the day of challenge,

• Chronic use (*more than once a week in any two [2] of the four [4] weeks preceding the first test article dose*) of any medication or other product (prescription or over-the-counter), for symptoms of rhinitis or nasal congestion or any chronic nasopharyngeal complaint, or chronic use of *any* intranasal medication for any indication,

- Any history during adulthood of asthma of any etiology,
- Subjects who smoke and who are unwilling or unable to desist for the duration of the inpatient challenge component of the study,
- Acute use of any medication or other product, prescription or over-the-counter, for symptoms of rhinitis or nasal congestion within seven (7) days prior to the first test article dose,
- Any anatomic or neurologic abnormality impairing the gag reflex or associated with an increased risk of aspiration, or history suggestive of such a problem,

• Receipt of systemic glucocorticoids (in a dose ≥ 5 mg prednisone daily or equivalent) within one (1) month, or any other cytotoxic or immunosuppressive drug within six (6) months,

• Receipt of any investigational drug within one (1) month, or prior participation in a clinical trial of any influenza vaccine within one (1) year,

- Presence of any febrile illness or symptoms (greater than grade 1, “mild”) of upper respiratory infection on the day of first test article administration. (Such subjects may be re-evaluated for enrollment after resolution of the illness),
- Presence of any febrile illness or symptoms suggestive of influenza between admission for influenza challenge and administration of the challenge inoculum,
- History of hypersensitivity to mercurials (found in antiseptics such as merthiolate, some vaccines and skin test reagents, and contact lens solutions) or chicken eggs.

# 5.0 Study Design:

This is a randomized, double-blind, placebo-controlled phase II study of the safety, immunogenicity, and protective efficacy against challenge of FluINsure™ proteosome-trivalent influenza vaccine delivered as a nasal spray. The study has two (2) sequential components, as detailed below.

5.1 Immunization Component:

FluINsure™ will be delivered as a one-dose or two-dose treatment regimen, with each individual dose containing 30 μg of each of three viral hemagglutinins (HA) regardless of the number of doses. The study will enroll approximately 75 healthy adult subjects selected for susceptibility to A/Panama/2007/99 into the immunization component.

Healthy normal subjects of both genders and 18 to 50 y.o. will be invited to give informed consent for preliminary screening phlebotomy, and then have a serum specimen obtained for baseline A/Panama/2007/99 HAI antibody testing. Screened subjects with serum reciprocal HAI titers ≤ 10 will be invited to return for a detailed explanation of the clinical trial and asked to give informed consent for participation in the trial itself. Consenting subjects will then undergo an assessment of their medical history, physical examination, ECG, and selected clinical laboratory tests to ensure eligibility according to the protocol. Two nasal wash samples for measurement of secretory IgA (nasal sIgA) specific for the vaccine virus strains will be obtained during screening.

5.1.1 Treatments:

Eligible subjects will be stratified by gender and then assigned to a treatment group according to a randomisation code prepared by the Sponsor. Treatment groups will receive:

Treatment Group A) Two intranasal spray doses, each dose containing 30 μg of each of three hemagglutinins (HA) formulated with proteosomes. The first dose will be given on day 0 and the second given after a 14-day interval.

Treatment Group B) Two intranasal spray doses, the first containing 30 μg of each of three hemagglutinins (HA) formulated with proteosomes, and the second containing buffered saline. The first dose will be given on day 0 and the second given after a 14-day interval.

Treatment Group C) Two intranasal spray doses of buffered saline. The first dose will be given on day 0 and the second given after a 14-day interval.

All dose volumes will be 0.28 mL (as 0.14 mL per nostril). Each treatment group will include approximately 25 subjects.

5.1.2 Safety and Tolerability Evaluation:

Following each test article administration, each subject will maintain a written memory aid of potential vaccine reactogenicity for seven (7) days; and this data will be collected by in-clinic interview two (2) days after each dose and by scripted telephone contact seven (7) days after each dose. In addition, a brief, directed examination of the nose, throat, ears, and cervical nodes will be done in the clinic two (2) days after each test article dose to detect reactions. Repeat clinical laboratory studies will be performed on day 28. Ascertainment of adverse events and changes in concomitant medications will be carried out through day 40 following the first test article dose, and again on day 60. Specific definitions and grading for vaccine reactogenicity and adverse events are outlined in section 8.2 and related appendices.

5.1.3 Immunogenicity Evaluation:

Venous blood and nasal wash specimens will be collected at specified intervals to examine the systemic and mucosal specific immune response. At day 40 following the first test article administration, a physical examination, venous blood collection for antigen-specific serologies, and collection of nasal wash specimens will complete the immunization component of the study.

5.2 Challenge Component:

On day 40, all subjects will be admitted to an inpatient isolation facility. Following admission, subjects will have vital signs ascertained four times per day, and will be questioned twice daily regarding symptoms of influenza or upper respiratory infection. Any subject developing fever (oral temperature > 37.9º C on two observations separated by at least 20 minutes) or complaints of rhinorrhea, nasal congestion, pharyngitis, or cough will be discharged to prevent introduction of an intercurrent upper respiratory infection to the population and thereby confounding of the clinical assessment.

On the morning of day 42, all remaining eligible subjects will receive a dose of viable, egg-grown A/Panama/2007/99 by intranasal drops. The challenge inoculum will be selected so as to achieve illness and infection rates ≥ 70%.

Between study days 42 (p.m.) and 49 (a.m.), subjects will complete a standard diary card of symptoms of influenza twice each day. During these days, oral temperatures will be measured approximately every six hours , and contents of the diary cards will be elicited by a directed interview and recorded, approximately every twelve (12) hours. Once per day, between days 42 and 49 and at approximately 24 hour intervals, each subject will have a directed physical examination of the ears, nose, throat, and chest. Once per day, between days 42 and 49 and at approximately 24 hour intervals, each subject will have a nasal wash and throat swabs for virus detection and titration. Beginning on day 48, each subject will begin five (5) days of twice-a-day treatment with a neuraminidase inhibitor (zanamivir or oseltamivir) to ensure termination of virus shedding. After completion of the a.m. diary card and vital sign determination on day 49, subjects who are afebrile and asymptomatic will be discharged. Any subject who is either febrile (temperature ≥ 37.9º C) or has persistent symptoms of grade 2 or greater (see diary card) will be retained in the facility for an additional 24 hours. Subjects remaining symptomatic will be referred for appropriate medical care. (Illness following inpatient influenza challenge is typically somewhat milder than community- acquired influenza, and short lived. This inpatient period, even without additional treatment with a neuraminidase inhibitor, extends beyond the known duration of virus shedding in this model.)

All subjects (challenged and unchallenged) will return on study day 60 and be queried regarding adverse events or changes in concomitant medications since discharge; a physical examination will be performed as needed to evaluate complaints and a follow-up ECG will be obtained. Serum and nasal wash specimens will be obtained for specific antibody determinations and the subjects discharged from the study.

# 6.0 Investigational Supplies:

## 6.1 Study Drugs:

Proteosome-trivalent influenza vaccine (*PINK LABEL*): a sterile, colorless to yellowish opalescent liquid containing 150 ± 30 μg / mL of hemagglutinin (HA) from each of A/New Caledonia/20/99 (H1N1), A/Panama/2007/99 (H3N2), and B/Victoria/504/2000 viruses in 10 mM Na/K phosphate-buffered isotonic saline, pH 7.4, with 0.01% thimerosal. Non-hemagglutinin viral proteins are also present. The virus components are formulated with outer membrane proteins (OMP) of *Neisseria meningitidis* strain 8047, at an initial ratio of OMPs to HA of 4:1. The overall total protein to hemagglutinin ratio in the final vaccine product is 2.5 – 5:1 (varies slightly with viral strain and non-hemagglutinin protein losses during the complexing process). The vaccine also contains trace amounts of egg proteins. The vaccine is delivered in 3 mL borosilicate glass vials containing a 0.9 mL fill.

Phosphate-buffered normal saline diluent/placebo (*WHITE LABEL*): a sterile, colorless liquid comprising 10 mM Na/K phosphate-buffered isotonic saline, pH 7.4, with 0.01% thimerosal, delivered in 20 mL borosilicate vials with a 10 mL fill.

## 6.2 Delivery System for Study Drugs:

Valois VP3/140 nasal spray pumps comprising a screw-top vial, an actuator, and a nose piece will be supplied. These devices deliver a metered dose of 140 μL of liquid as an atomized spray with each full depression of the actuator. Delivery of the test article volume by this device has been shown to be ± 5% of nominal volume with each discharge, and median droplet size in the spray is 40 – 50 μm.

## 6.3 Storage Requirements for Study Drugs:

Proteosome-trivalent influenza vaccine should be stored at a controlled temperature of 5 ± 3° C in a secured refrigerator until use. It should NOT be frozen. The vaccine should be used within eight (8) hours of dilution of the stock material and within four (4) hours of removal from refrigeration. While the vaccine contains thimerosal, stock vaccine vials are NOT intended for repeated use over multiple days. Thus, while several doses may be prepared from a single vial of stock vaccine on a given day, residual contents of an opened vial must NOT be used thereafter.

Phosphate-buffered saline should be stored at room temperature away from direct light. Once opened, the contents of a vial may be used for up to eight (8) hours, but residual contents must NOT be used thereafter.

## 6.4 Test Article Dose Preparation:

### 6.4.1 Randomization / Study Day 0:

On Study Day 0, the investigational pharmacist (or another unblinded member of site staff who is designated to serve as pharmacist and will have NO involvement whatever with any clinical outcome measurement) will be provided with each eligible, and consenting subject’s initials, subject ID number (SID), and gender. The investigational pharmacist will use the gender-specific randomization list provided by the sponsor to assign the next available randomization code and determine treatment assignment. For each subject randomized, a label bearing the subject’s SID, initials, and randomization code (a two-digit code that does not itself, in the absence of the unblinded treatment list, reveal treatment assignment) will be prepared and affixed to a metered dose pump. In addition, the SID will be transcribed onto the Treatment Group Enrollment Log. The investigational pharmacist will then prepare the appropriate test article for each subject.

For Day 14 doses, the investigational pharmacist will consult the Treatment Group Enrollment Log to confirm the appropriate treatment group for each subject, and hence the appropriate test article, and then prepare a label bearing the subject’s SID, initials, and randomization code and affix it to a metered dose pump.

### 6.4.2 Preparation of Individual Test Article Doses:

A test article dose will be prepared for each subject to be dosed by placing a total of 0.9 mL of appropriately diluted active vaccine or placebo/diluent, as dictated by the treatment group assignment, into the vial of the previously labeled metered dose pump, after which the actuator and nose piece will be assembled to the pump. Since the target concentrations of each of the three HA species in the vaccine are approximately equal, dosing and dilution will be based on the mean HA concentration in the trivalent (i.e., the average of the concentrations of the three different HAs). Detailed instructions and dose preparation worksheets are provided as Appendix A. After assembly of the pump, the pharmacist will hold the metered dose pump upright, gently swirl once again, remove the nosepiece and then the safety collar, and firmly and fully depress the actuator three (3) times to prime the pump. The collar and then the nosepiece cover will be replaced after priming, and the labeled, filled, and primed pump will be delivered to the clinic.

After test article preparation is completed for a given day, any opened stock vaccine and/or saline placebo vials will be restoppered and retained for retrieval by an ID Biomedical representative.

## 6.5 Test Article Dose Administration:

All study drugs are to be administered under the supervision of the Investigator or a qualified physician subinvestigator / study doctor designated to ID Biomedical in writing prior to the trial and trained in both the protocol and contents of the Investigators’ Brochure. Under no circumstances will the Investigator allow study drugs to be used other than as directed by this protocol.

### Test articles will be administered as follows:

• The nosepiece cover will be removed, and then the safety collar.

- The nose piece of the metered dose pump will be placed in the subject’s right nostril while the subject occludes the left.

• As the subject gently inhales through the nose, the actuator will be firmly and fully depressed.

• The nose piece of the metered dose pump will be placed in the subject’s left nostril while the subject occludes the right.

- As the subject gently inhales through the nose, the actuator will be firmly and fully depressed.

• The used pump will be retained for drug accountability and eventual return to the sponsor.

## 6.6 Accountability for Study Drugs:

The Investigator or a designee is responsible for maintaining complete drug inventory records accounting for receipt, storage, dispensation, and final disposition using forms supplied by (or local equivalents approved by) ID Biomedical. These records will be reviewed by ID Biomedical representatives. At the conclusion of the trial all vials of study drugs, used and unused, and all metered dose pumps will be returned to the sponsor.

6.7 Challenge Virus:

The challenge virus is A/Panama/2007/99 (H3N2) lot I B 44/3 produced in eggs by Berna Biotech Ltd. This material is sterile and mycoplasma-free. The titer of this virus as provided by the manufacturer is 10^8.46^ egg ID_50_ / mL, and it is provided in cryovials. Prior to challenge, the virus will be diluted to the predetermined desired challenge inoculum in sterile isotonic phosphate-buffered saline.

6.8 Storage of Challenge Virus:

Challenge virus should be stored at ≤ - 70º C in a secured freezer until use. Vials should be thawed just prior to dilution. Once thawed and entered, vials of stock virus should NOT be reused for human studies.

6.9 Disposal of Challenge Virus:

Used challenge virus stock vials and dilutions will be disposed of according to Retroscreen Ltd’s SOPs for the destruction of biohazardous waste.

# 7.0 Procedures and Methods:

## 7.1 Procedures:

The following procedures will be done at the indicated times (see also Appendix B: IDB-13004 Time and Events Tables B.1 and B.2).

### 7.1.1 First Screening Visit (days - 90 to - 7):

• Written, witnessed informed consent for screening will be obtained.

- A brief medical history will be obtained to ensure that the subject meets historical criteria for enrollment, and approximately 10 mL of blood will be drawn for A/Panama/2007/99 serum HAI antibody screening determination (see section 7.2).

### 7.1.2 Second Screening Visit (days -42 to - 5):

- This visit will occur within the above interval, but not prior to completion of A/Panama/2007/99 HAI determination on the “first screening” specimen.
- Subjects’ eligibility for enrollment based on history and HAI data will be reviewed.
- A detailed explanation of the clinical trial will be provided to subjects remaining eligible, and written, witnessed informed consent to trial participation will be elicited.
- Vital signs will be recorded.
- An ECG will be performed.
- Approximately 25 mL of blood will be drawn and a urine specimen obtained for baseline clinical laboratory tests, and HIV 1 & 2 serologies (see section 7.2).
- A nasal wash specimen for specific influenza antibody assay will be obtained (section 7.2).

7.1.3 Final Screening Visit (days -14 to – 2):

- This visit should take place not less than three (3) days after from the second screening visit.
- Subjects’ eligibility for enrollment based on ECG and clinical laboratory data from the prior screening visit will be reviewed.
- Approximately 40 mL of blood will be drawn for baseline HAI antibody testing and study of cytokine secretion by mononuclear cell fractions (see section 7.2).
- A nasal wash specimen for specific influenza antibody assay will be obtained (section 7.2).
- A complete baseline physical examination, with vital signs, may be performed and recorded at this visit or deferred to Day 0 (see section 7.1.5).

### 7.1.4 Day 0, First Treatment:

• An interval medical history will be performed to ensure that each subject continues to meet all inclusion and no exclusion criteria.

- A urine specimen will be collected from each female subject and tested in the clinic for evidence of pregnancy. No test article will be administered until a negative result is obtained and documented.
- A complete baseline physical examination, including vital signs, will be performed and recorded if not completed at the final screening visit. This examination will also include a directed examination of the ears, cervical lymph nodes, and nasopharynx, the results of which will be recorded using the worksheet provided as Appendix C. Subjects for whom a complete baseline examination was recorded at the second screening visit will have only vital signs and the directed examination of ears, cervical lymph nodes and nasopharynx performed and recorded.
- Each subject will be provided with a Memory Aid (Appendix D) and a digital thermometer and instructed to take his/her temperature and complete a Memory Aid page each evening from Day 0 to Day 6. Volunteers will complete the baseline Memory Aid observations prior to dosing.
- Volunteers with any grade 2 physical findings or grade 2 symptoms among their baseline Memory Aid observations will not be given a test article dose. Such volunteers may return at a later time point and be considered for enrollment if findings/symptoms resolve.
- Test article will be administered intranasally as described in section 6.5. (*Subjects who receive any amount of test article will be considered enrolled and followed through day 60 for safety and immunogenicity as specified below and in section 8.0. No additional follow-up will be required of subjects who are screened but do not receive any test article*.)

• Subjects will be observed for thirty (30) minutes after dosing. At the end of this interval, each subject will have a repeat oral temperature measured and recorded and complete a questionnaire eliciting immediate post-immunization symptoms (present at the time of observation or in the preceding 30 minutes). Each will be graded as: none, mild (just noticeable), moderate (unpleasant/uncomfortable, but not incapacitating), or severe (preventing resumption of normal activities). The questionnaire is provided as Appendix E.

• Subjects will be instructed to return to the clinic (Retroscreen) on Day 2, with their Memory Aid.

### 7.1.5 Day 2 (day 3 acceptable):

• Vital signs will be taken and recorded.

• Each subject’s Memory Aid will be reviewed and the data abstracted and recorded. Subjects who have lost their Memory Aids will be provided with new documents, which will be completed from memory with the assistance of the interviewer. Such data will be clearly annotated as RETROSPECTIVE.

• Subjects will be queried regarding the occurrence of adverse events not specified in the Memory Aid, and any new concomitant medications taken, and the results recorded.

• A brief, directed examination of the ears, cervical lymph nodes, and naso-pharynx will be performed to detect objective signs of vaccine-induced reactogenicity. The examiner will complete the brief exam worksheet provided as Appendix C.

• Subjects will be instructed to maintain their Memory Aids, will be given an appointment for scripted telephone interview on Day 7, and an appointment to return to the clinic on Day 12 ± 1.

### 7.1.6 Day 7 (± 1 day) Telephone Follow-up:

• Each subject will be contacted by telephone and the symptoms listed on the Memory Aid will be elicited by scripted interview and recorded.

• Subjects will be queried regarding the occurrence of adverse events not specified in the Memory Aid, and any new concomitant medications taken, and the results recorded.

• Any subject reporting an ongoing grade 3 complaint, or another adverse event which is moderate or severe in intensity, will be asked to return to the clinic for examination.

• Subjects will be reminded to return for their next clinic visit on day 12 ± 1.

7.1.7 Day 12 (± 1 day):

- Vital signs will be taken and recorded.
- Subjects will be queried regarding the occurrence of adverse events not specified in the Memory Aid, and any new concomitant medications taken, and the results recorded.
- A nasal wash specimen for specific antibody assay will be obtained (section 7.2).
- Approximately 15 mL of blood will be drawn from each subject for specific serologies (see section 7.2).
- Subjects will be reminded to return for their Day 14 visit.

### 7.1.8 Day 14 (± 2 days, but not within 24 hours of the Day 12 visit):

- Vital signs will be taken and recorded
- An interval medical history will be performed to ensure that each subject continues to meet all inclusion and no exclusion criteria.
- A urine specimen will be collected from each female subject and tested in the clinic for evidence of pregnancy. No test article will be administered until a negative result is obtained and documented.
- A directed examination of the ears, cervical lymph nodes, and nasopharynx will be performed, the results of which will be recorded using the worksheet provided as Appendix C.
- Each subject will be provided with a Memory Aid (Appendix D) and a new digital thermometer (if needed) and instructed to take his/her temperature and complete a Memory Aid page each evening from Day 14 to Day 21. Volunteers will complete the baseline Memory Aid observations prior to dosing.
- Volunteers with any grade 2 physical findings or grade 2 symptoms among their baseline Memory Aid observations will not be given a test article dose.
- These subjects may return to the clinic up to day 21 and, if symptoms or findings have resolved, receive the second test article dose. Up to day 16, this will not be recorded as a protocol deviation; from day 17 to 21 a deviation will be recorded but the dose will be given. Such subjects will complete all protocol-mandated follow-up per schedule and *will* be eligible for challenge.
- If grade 2 symptoms or findings persist beyond day 21, the second dose will be withheld. Such subjects will not be eligible for challenge, but will complete all other protocol-mandated follow-up.

• Test article will be administered intranasally as described in section 6.5.

• Subjects will be observed for thirty (30) minutes after dosing. At the end of this interval, each subject will have an oral temperature measured and recorded and complete a questionnaire eliciting immediate post-immunization symptoms (present at the time of observation or in the preceding 30 minutes). Each will be graded as: none, mild (just noticeable), moderate (unpleasant/uncomfortable, but not incapacitating), or severe (preventing resumption of normal activities). The questionnaire is provided as Appendix E.

• Subjects will be instructed to return on Day 16, with their Memory Aid.

### 7.1.9 Day 16 (day 17 acceptable):

• Vital signs will be taken and recorded.

• Each subject’s Memory Aid will be reviewed and the data abstracted and recorded. Subjects who have lost their Memory Aids will be provided with new documents, which will be completed from memory with the assistance of the interviewer. Such data will be clearly annotated as RETROSPECTIVE.

• Subjects will be queried regarding the occurrence of adverse events not specified in the Memory Aid, and any new concomitant medications taken, and the results recorded.

• A brief, directed examination of the ears, cervical lymph nodes, and naso-pharynx will be performed to detect objective signs of vaccine-induced reactogenicity. The examiner will complete the brief exam worksheet provided as Appendix C.

• Subjects will be instructed to maintain their Memory Aids, will be given an appointment for scripted telephone interview on Day 21, and an appointment to return to the clinic on Day 28 ± 2.

7.1.10 Day 21 (± 1 day) Telephone Follow-Up:

- Each subject will be contacted by telephone and the symptoms listed on the Memory Aid will be elicited by scripted interview and recorded.

• Subjects will be queried regarding the occurrence of adverse events not specified in the Memory Aid, and any new concomitant medications taken, and the results recorded.

• Any subject reporting an ongoing grade 3 complaint, or another adverse event which is moderate or severe in intensity, will be asked to return to the clinic for examination.

• Subjects will be reminded to return for their next clinic visit on day 28 ± 3.

### 7.1.11 Day 28 (± 3 days):

- Subjects will be queried regarding the occurrence of adverse events and any new concomitant medications taken, and the results recorded.
- A physical examination will be performed *if required* to evaluate any complaints, and new abnormalities will be captured as adverse events.

• A nasal wash specimen for specific antibody assay will be obtained from each subject (see section 7.2).

• Approximately 60 mL of blood will be drawn from each subject for HAI antibodies, studies of cytokine secretion by mononuclear cells, and clinical laboratory tests (see section 7.2).

- Each subject will provide a urine specimen for determination of blood, glucose, and protein.

### 7.1.12 Day 40 (± 3 days), Admission for Challenge:

• All subjects will be admitted to the isolation unit.

- An interval medical history will be performed to ensure that each subject continues to meet all inclusion and no exclusion criteria (excepting influenza vaccine and serum HAI criteria, which will have become irrelevant due to the immunization component of this protocol).
- Subjects will be queried regarding the occurrence of adverse events and changes in concomitant medications, and the results recorded.
- Subjects will complete an Influenza Symptom Diary Card (see Appendix F), and will be interviewed by a physician re: potential symptoms of influenza or upper respiratory infection.
- A complete physical examination, including vital signs with oral temperature, will be performed and recorded.
- An ECG will be performed.

• A nasal wash specimen for specific antibody assay will be obtained from each subject (see section 7.2).

• Approximately 15 mL of blood will be drawn from each subject for specific serologies (see section 7.2).

7.1.13 Day 40 Post-admission to Day 41:

• Subjects will have oral temperature measured and recorded approximately every six (6) hours.

- Subjects will complete an Influenza Symptom Diary Card (see Appendix F), and will be interviewed by a physician, approximately every 12 hours.
- Subjects will be queried regarding occurrence of AEs not specified in the Influenza Symptom Diary Card, and these will be recorded.
- Any subject with complaints consistent with influenza and/or an oral temperature ≥ 37.9 º C (confirmed by repetition at an interval of not less than 20 minutes) will be immediately segregated from the remaining subjects, examined by the physician, and discharged if any suspicion of influenza or other respiratory infection exists. The examining physician will provide medical referral for outpatient care if deemed necessary. The cause of such discharge will be recorded as an adverse event. The subject will be instructed to return for the day 60 outpatient visit.

### 7.1.14 Day 42 a.m. (Day of Challenge):

- Subjects will complete a morning Influenza Symptom Diary Card (see Appendix F).
- Subjects will be queried regarding occurrence of AEs not specified in the Influenza Symptom Diary Card, and these will be recorded.
- A urine specimen will be collected from each female subject and tested in the clinic for evidence of pregnancy. No challenge virus will be administered until a negative result is obtained and documented.
- Addressing the items on the Challenge Physical Examination Worksheet (see Appendix G), a baseline physical examination, including vital signs, will be performed and recorded. Baseline tympanometry will be performed and the results recorded.
- Any subject with complaints consistent with influenza and/or an oral temperature ≥ 37.9 º C (confirmed by repetition at an interval of not less than 20 minutes) will be immediately segregated from the remaining subjects and discharged if any suspicion of influenza or other respiratory infection exists. The examining physician will provide medical referral for outpatient care if deemed necessary. The cause of such discharge will be recorded as an adverse event. The subject will be instructed to return for the day 60 outpatient visit.

• Each subject remaining eligible will provide a throat swab for molecular detection of influenza virus (see section 7.2).

• Each subject remaining eligible will be challenged with A/Panama/2007/99 delivered nasally by dropper.

7.1.15 Day 42 p.m. to Day 49:

- Subjects will complete an Influenza Symptom Diary Card (see Appendix F), and will be interviewed by a physician, approximately every 12 hours.
- Subjects will have an oral temperature measured and recorded approximately every six (6) hours.
- Subjects will be queried regarding occurrence of AEs not specified in the Influenza Symptom Diary Card, and these will be recorded.
- Using the Challenge Physical Examination Worksheet (see Appendix G), a physical examination, including vital signs, will be performed and recorded approximately every 24 hours.
- Beginning on day 43, tympanometry will be performed and the results recorded approximately every 24 hours.
- Every day (beginning day 43), *after physical examinations are completed*, a nasal wash for virus culture and a throat swab for molecular detection of influenza virus will be obtained from each subject (see section 7.2).
- Symptomatic treatment with paracetamol (650 mg orally no more than q.i.d.) will not be offered, but may be given upon subject request *if and only if*:
  - A subject has an oral temperature > 38.9º C on two occasions separated by not less than 20 minutes, or
  - A subject has an oral temperature of 37.9 – 38.9º C, and has met the definition for any two of upper respiratory illness, lower respiratory illness, or systemic illness (section 9.3).
- After the a.m. physician interview of day 48, all subjects will initiate treatment with zanamivir (10 mg inhaled) or oseltamivir (75 mg orally), either drug to be administered twice daily at approximately 12 hour intervals for five (5) days.

7.1.16 Day 49:

- Zanamivir or oseltamivir treatment will continue as per 7.1.15.
- Subjects will be queried regarding occurrence of AEs not specified in the Influenza Symptom Diary Card, and these will be recorded.
- A complete physical examination, including vital signs, will be performed and recorded.
- An ECG will be performed.
- After the physical examination, a nasal wash for viral culture and a throat swab for molecular detection of influenza virus will be obtained from each subject (see section 7.2)
- Subjects who are afebrile (oral temperature < 37.9º C) and free of symptoms of influenza-like illness may be discharged with instructions to return to the clinic for the day 60 outpatient visit. Subjects will be supplied with sufficient zanamivir or oseltamivir to complete their five-day regimen, and reminded to do so. Any subject who is either febrile (temperature > 37.9º C) or has persistent symptoms of grade 2 or greater (see diary card) will be retained in the facility for 24 hours additional observation and drug treatment. Subjects remaining symptomatic thereafter will be referred for appropriate medical care.

7.1.17 Day 60 (± 3 days):

- Subjects will be queried regarding the occurrence of adverse events and any new concomitant medications taken, and the results recorded.
- A complete physical examination, including vital signs, will be performed and recorded.
- An ECG will be performed.
- A nasal wash specimen for specific antibody assay will be obtained from each subject (see section 7.2).
- Subjects will provide a 40 mL sample of blood for HAI antibody testing and studies of cytokine secretion by mononuclear cells (see section 7.2).
- This visit marks completion of protocol IDB-13004.

## 7.2 Laboratory Tests:

### 7.2.1 Hematology:

The following will be done at the second screening visit and on day 28 (± 3 days), and if clinically indicated to evaluate illness during challenge.

• Hematocrit

• Hemoglobin

• White blood cell count and differential

• Platelet count

### 7.2.2 Clinical Chemistry:

The following will be done at the second screening visit and on day 28 (± 3 days) and if clinically indicated to evaluate illness during challenge.

• ALT and AST

• Blood urea nitrogen

• Creatinine

### 7.2.3 Clinical Laboratory Serologies:

The following serum determinations will be done at the second screening visit:

• Anti-HIV 1 & 2 antibodies

### Urinalysis:

The following analytes will be recorded at the second screening visit and on day 28 (± 3 days) and if clinically indicated to evaluate illness during challenge.

- Glucose
- Protein
- Hemoglobin

Any sample with any positive glucose, or any protein or hemoglobin > trace, will trigger performance of a complete urinalysis, including microscopic examination.

### 7.2.5 Pregnancy Testing:

Urine β-HCG determination will be performed on a freshly-obtained urine specimen on the day of each test article dose and on the day of challenge dosing on each female subject not having a verifiable history of hysterectomy. A negative result must be obtained and recorded before any test article or challenge inoculum is administered.

### 7.2.6 Specific Antibody Assays:

• Serum will be obtained for influenza-specific special serologies (serum hemagglutination-inhibition titers) at the first screening visit (A/Panama/2007/99 only), at the final screening visit (all three strains tested for this and subsequent samples), and on days 14 (± 1), 28 (± 3), 40 (± 3), and 60 (± 3). Should hemagglutination-inhibition prove unworkable for the influenza B virus component, single radial hemolysis assay may be utilized.

- Nasal washes will be obtained for influenza-specific IgA at the second and final screening visits, and on days 12 (± 1), 28 (± 3), 40 (± 3) and 60 (± 3).

7.2.7 Studies of Cytokine Induction :

Approximately 25 mL of venous blood will be obtained for purification of peripheral blood mononuclear cells at the final screening visit (days -14 to -2), on day 28 (± 3) and on day 60 (± 3). Cells will be restimulated *in-vitro* with inactivated whole influenza virus to detect shifts in patterns of cytokine elaboration induced by immunization.

### 7.2.8 Influenza Virus Detection:

A nasal wash for influenza virus titration in tissue culture and by quantitative polymerase chain reaction (PCR) assay, and a throat swab for PCR will be obtained daily from day 42 to day 49.

## 7.3 Other Measurements:

### 7.3.1 History and Physical Examination:

A complete physical examination and medical history/review of systems will be performed and recorded before the first test article dose. Vital signs will be included in all physical examinations. Significant abnormalities in the physical

examination will be recorded on the case report forms. The medical history should record significant problems active at the time of screening or within the last year. Problems which have been inactive within the last year, but which might alter the subject’s current or future management, should also be noted (e.g., known mitral valve prolapse, history of seizure disorder, etc.) The physical examination will be repeated as needed for evaluation of complaints during the trial, and a complete physical examination will be repeated prior to challenge (on day 40 ± 3). A third complete examination will be performed on day 49 prior to discharge from the isolation facility. Additional repeat examinations will be performed on day 60 only as necessary to evaluate any new complaints. Any new and clinically-significant abnormalities will be recorded as adverse events.

### 7.3.2 Brief, Directed Physical Examination after Treatments:

A brief, directed physical examination will be performed on days 0, 2 or 3, 14 and 16 or 17 to ensure eligibility for treatment (days 0 and 14) and to evaluate the presence of objective findings of vaccine reactogenicity (days 2 or 3 and 16 or 17). This examination will encompass the ears, nose, throat, and cervical lymph nodes, and will be recorded using a standardized worksheet (Appendix C). It is emphasized to site staff that continuity of observer for any given subject is important for these examinations and should be maintained if at all practicable.

7.3.3 Directed Physical Examination During Challenge:

From day 42 a.m. to day 49 a.m., vital signs will be recorded at least every 12 hours. A directed physical examination will be performed and recorded approximately every 24 hours in accordance with the Challenge Physical Examination Worksheet (see Appendix G). The physician investigator may, at his/her judgement, perform any additional vital signs or physical examination necessary to evaluate or manage clinical illness. It is emphasized to site staff that continuity of observer is again desirable and should be maintained if at all practicable.

### 7.3.4 Concomitant Medications:

Subjects should be instructed not to introduce new medications without consulting or notifying the investigator or his designee. Interval medication cards (Appendix F) will be provided as memory aids and subjects will be questioned regarding new medications at each visit through day 60 ± 3 days; and any such medications recorded on the case report form.

## 7.4 Early Discontinuation:

Every reasonable effort should be made to ensure that each subject complies with the protocol and completes all study visits. However, a subject may withdraw or be withdrawn from participation if:

• The subject withdraws consent,

- The investigator recommends discontinuation in the interest of the subject’s safety or because of significant and irremediable protocol non-compliance,

• ID Biomedical, the ethics committee, or the MCA terminates the clinical trial.

Subjects must be stringently counseled that early withdrawal from the challenge isolation portion of the trial will be strongly discouraged, as it poses a risk both to the subject and to his/her contacts. Any subject insisting on early withdrawal during challenge isolation will be started immediately on a five (5) day course of a neuraminidase inhibitor, and will be encouraged to remain in the isolation unit as long as possible.

A complete Day 60 evaluation and Study Termination case report form should be completed on any subject prematurely withdrawn from the clinical trial. No subject prematurely withdrawing/withdrawn will be replaced.

# 8.0 Immediate Complaints, Vaccine Reactogenicity, Findings of Influenza and Adverse Events:

## Immediate Complaints:

A selection of immediate complaints reasonably anticipated to occur as a result of receipt of the test articles is provided as a questionnaire (Appendix E) to be completed approximately 30 minutes after each test article dose and transcribed to the case report forms. The data will be reported as immediate complaints and presumed to be related to the test article. *These findings should NOT be additionally recorded as adverse events (see below) unless they fulfill the criteria set forth in section 8.4.1.*

## 8.2 Vaccine Reactogenicity:

A selection of subjective complaints reasonably anticipated to occur as a result of receipt of the test articles are provided in the Memory Aid (see sample in Appendix D), and a selection of potential physical findings also reasonably anticipated to occur as a result of receipt of the test articles are provided on the Brief Examination Worksheet (Appendix C) to be used on days 0, 2, 14, and 16. Findings from these two sources will be recorded on the case report forms, reported as vaccine reactogenicity, and presumed to be related to the test article(s). *These findings should NOT be additionally recorded as adverse events (see below) unless they fulfill the criteria set forth in section 8.4.1.*

Definitions used to grade reactogenicity events are included as an integral part of each Brief Examination Worksheet (Appendix C) and Memory Aid page (Appendix D).

8.3 Symptoms and Findings of Influenza:

During the challenge period (Days 42 – 49), symptoms and signs of influenza will be collected using the Influenza Symptom Diary Card (see Appendix F) and the Challenge Physical Examination Worksheet (see Appendix G) and their integral definitions. During this interval, findings captured by these two tools will be presumed to represent influenza consequent to challenge, and *will not be additionally captured as adverse events (see below) unless they fulfill the criteria set forth in section 8.4.1.*

## 8.4 Adverse Events:

An adverse event (AE) is any unfavorable, harmful, or pathologic change in a research subject as indicated by physical signs, symptoms and/or clinically significant laboratory abnormalities that occurs in association with the use of a product (trial-related), whether or not considered to be product-related. This includes intercurrent illnesses, injuries, worsening of pre-existing conditions, and events occurring as a result of product abuse or overdose. Stable pre-existing conditions and/or elective procedures to address them are not adverse events. Clinical laboratory findings are considered to be adverse events if regarded as clinically significant by the investigator, or if these cause (or should have caused) a change in the investigational drug regimen, a further diagnostic evaluation, or institution of any therapy.

All events fulfilling any part of the AE definition must be recorded on the adverse event case report form (with the exception of pre-defined immediate complaints or symptoms/signs of vaccine reactogenicity in the first seven [7] days following immunizations, and pre-defined symptoms and signs of influenza during the challenge (days 42 – 49); see sections 8.1, 8.2, 8.3, and 8.4.1).

Treatments and procedures are not adverse events; rather, the illness which precipitates them should be recorded. For example, “cholecystectomy,” is not an AE, but the diagnosis of “cholecystitis” or “gall stones” leading to the surgical (or medical) treatment is an AE. Where possible, specific diagnoses are preferred in reporting AEs. Individual complaints or findings may be reported as AEs, but when multiple complaints or findings occur together and can be logically and defensibly assembled into a single clinical syndrome or diagnosis, the latter is preferred. For example, an isolated finding of elevated AST and ALT would be recorded as “elevated transaminases.” If accompanied by fever, anorexia, nausea, and jaundice, however; a report of “hepatitis” would be preferable to a list of individual findings. If the new presence of HBsAg is also found, a more specific diagnosis of “hepatitis B” would be recorded.

### 8.4.1 Double Reporting of Reactogenicity Findings/Complaints and Findings of Influenza as Adverse Events:

As per sections 8.1, and 8.2 above, findings and/or complaints specifically captured by the Immediate Complaints Questionnaire (Appendix E), the Brief Examination Worksheet (Appendix C), or the Memory Aid (Appendix D) should NOT, in general, be additionally recorded as AEs if they occur within seven (7) days of a test article dose.

The rationale for not in general doubly-reporting reactogenicity events as AEs is as follows. The specified range of reactogenicity complaints and findings in Appendices C, D, and E are those reasonably expected (theoretically and by experience) to be associated with a nasal vaccine. They are collected, *presumed to be drug-related*, analyzed, and reported to investigators and regulatory authorities in detail as expected vaccine reactions. Separation of these numerically very frequent, but minor and anticipated, vaccine reactions from other classes of adverse events prevents the obscuration of other potentially important, but less common, types of events in the statistical analysis of AEs.

There are *three exceptions* to the rule regarding double reporting:

1. Any of these findings or complaints which fulfills the definition of “serious adverse event” (see section 8.5) should be recorded as an AE (and reported as per section 8.5),
2. Any of these findings or complaints that persists beyond seven (7) days after a test article dose should be recorded as an AE,
3. Any of these findings or complaints which the investigator *unequivocally* categorizes, based on strong clinical evidence, as unrelated to the test article should be recorded as an AE. In this case the investigator *must* assign a causality of “probably not related” (not “unknown”) and record a clear rationale in source documents.

If a reactogenicity finding or complaint is also recorded as an AE, site personnel should take care to record a verbatim AE identical to the wording of the reactogenicity finding/complaint, and also an AE start date identical to the onset

date implied by the reactogenicity case report forms. The sponsor will report these events as both reactogenicity and AE findings, but will be able to link the two reports in the database for discussion and analysis purposes.

As per section 8.3 above, the symptoms and physical findings of influenza captured by the Influenza Symptom Diary Card (Appendix F) and the Challenge Physical Examination Worksheet (Appendix G) will also not in general be additionally recorded as AEs. The sole exception to this rule will be that symptoms or physical findings of influenza which fulfill, singly or collectively, the definition of “serious” as per section 8.5 will be recorded as serious AEs and be reported in accordance with section 8.5.

## 8.5 Serious Adverse Events:

A serious adverse event (SAE) is any adverse event that results in any of the following outcomes: a) death, b) an immediate threat to life, c) inpatient hospitalization or prolongation of an existing hospitalization, d) persistent or significant disability / incapacity, or e) a congenital anomaly / birth defect. Important medical events that do not result in one of these outcomes, but, based on appropriate medical judgement, are deemed to jeopardize the subject or require medical or surgical intervention to avert one of the listed outcomes, may also be considered SAEs. Serious adverse events must be reported to ID Biomedical within 24 hours of the investigator’s learning of the event. The initial report may be verbal, or may utilize the SAE worksheet provided by ID Biomedical and be transmitted by facsimile. Reports may be made to:

Dr. Louis F. Fries, at:

• 01−410-455-5610 (office)

• 01−410-455-5606 (fax)

• 01−443-253-5298 (mobile)

• 01−410-964-1326 (home)

Or alternatively: Dr. Peter Vink, a back-up contact, at:

• 01−410-455-5613 (office)

• 01−410-455-5606 (fax)

In the case of a verbal report, a written report using the ID Biomedical, SAE worksheet (which may subsequently be updated if additional data become available) should be provided by facsimile within 72 hours. All SAEs must also be reported as soon as possible to the ethics committee reviewing and approving the clinical trial.

# 9.0 Analysis Plan:

This is a phase II, three-group, placebo-controlled, randomized clinical trial with a cross-over design. Approximately 25 subjects will be treated in each group. With treatments separated by two weeks, active and placebo test articles are used in the two treatments, yielding four possible combinations (from active-active to placebo-placebo) of which three appear in this study (the placebo-to-active sequence is omitted). In the immediate post-treatment period, outcomes are pre-specified local and systemic reactions, general adverse event rates, and immune responses assessed by antibody measurements.

On day 42, 60 subjects, 20 per cross-over group, are challenged and then followed for an additional week regarding evidence of illness (by several alternative definitions), and also concerning virologic outcomes.

All analyses will be done with SAS, version 8.2 or later, primarily PROC UNIVARIATE, FREQ, LOGISTIC (for logistic regression with challenge data), and GENMOD (SAS PROC GENMOD implements the repeated measures regression model of Zeger and Liang [46].) Chi-square tests in PROC FREQ are exact. P-values of 0.05 or less for two-sided statistical tests will be considered significant and multiple comparison corrections will not be used although the rather large number of significance tests makes it likely that the type I error rate will be elevated.

Simple methods will be used to impute missing data. For binary outcomes, such as the presence/absence of an adverse event, alternative imputations will assume the event did, and did not, occur and the results of these contrary assumptions will be compared. For continuous outcomes, such as antibody titers, last-value-carried-forward will be used except that two consecutive missing values will not be imputed.

## 9.1 Immunogenicity:

Immunogenicity measures will include serum hemagglutination-inhibiting (HAI) antibody titers specific for the three virus strains included in the vaccine, and levels of secretory IgA (sIgA) specific for these viruses measured in nasal wash fluids. Antibody levels in nasal wash specimens will be assayed by kinetic ELISA and normalized on total content of sIgA. For the HAI titers, analyses will concern geometric mean titers (GMT), proportions of subjects with titer ≥ 40, and proportions with ≥ four-fold increase over baseline. For specific sIgA levels, analyses will concern geometric means and fold-rise from baseline.

HAI GMTs and geometric mean nasal specific sIgA levels will be accompanied by 95%, Student’s t-based confidence intervals. Between active-to-placebo and active-to-active cross-over groups, titers/antibody levels will be compared on day 28 by t-tests on log_10_ (titer). Within the groups, paired tests will be used (Student’s t and Wilcoxon signed rank). Fold-rises may be examined with and without covariate-adjustment for previous titers. Without such adjustment, there are two binary fold-rise outcomes, and these are compared within and between groups by chi-square and stratified chi-square tests. (In the latter case, 2 x 2 tables of treatment by outcome are stratified by previous titers.) With covariate adjustment, the fold-rise outcome is the log_10_-ratio of early and later titers. Adjustment after the first treatment is for baseline titer and after the second treatment is for both baseline titer and titer after the first treatment. This facilitates contemporaneous between-group comparisons of placebo and active after both periods 1 and 2.

9.2 Safety:

### 9.2.1 Power and Detectable Effect Size:

In regard to the immediate post-treatment component of the study design, we address the following two questions: What must be the chance of a given reactogenicity event/finding or adverse event in a single subject for there to be a high likelihood (probability 0.80) of a few occurrences in 25 and 50 subjects? (No more than 50 subjects will receive the active product.) Comparing two cross-over groups in the same period, what must be the relative risk of a reactogenicity event/finding or adverse event for the groups to differ significantly in risk? Exact calculations are based on the binomial distribution.

| Chance of an event in a single subject so that there is an 80% chance of at least the given number of events in 25 or 50 subjects | | | |
| --- | --- | --- | --- |
|  | ≥ 1 | ≥ 2 | ≥ 3 |
| n = 25 | 0.0625 | 0.115 | 0.164 |
| n = 50 | 0.032 | 0.058 | 0.083 |

Thus, events that occur with probability 6% and 3% in a single subject imply an 80% chance of seeing at least one event in, respectively, 25 and 50 subjects. This phase II study is not intended to be powered to detect rare events.

Turning to comparison of any two cross-over groups, 25 subjects in each, in the same period, we compute the relative risk (RR) that gives 80% power, type I error 5%, to detect RR ≠ 1.

| Chance of an adverse event in a placebo vaccinee so that a given risk-ratio yields a significant effect (80% power, 5% type I error) | | | |
| --- | --- | --- | --- |
| relative risk | 5-fold | 3-fold | 2-fold |
| placebo rate | 0.095 | 0.203 | 0.405 |

Thus, for a five-fold relative risk of an adverse event, active-to-placebo, to be detectable (with good power), the chances of an adverse event in the placebo group must be 0.095. With a lower adverse event rate among placebo subjects, there would not be good power to detect even a five-fold increase in risk However, for the most frequent reactogenicity events observed with intranasal proteosome-influenza vaccine, mild rhinorrhea and nasal congestion, prior experience indicates that placebo rates will range from 0.2 – 0.3. Thus, there will be adequate power to detect two- to three-fold increases in risk in active product recipients.

### 9.2.2 Immediate Complaints, Vaccine Reactogenicity Complaints, and Standardized Ear, Nose, and Throat Exams:

Immediate complaint data derived from 30 minute post-dose questionnaires will be tabulated for each treatment group by categories of complaints and by severity. Vaccine reactogenicity data derived from memory aids and physician examinations will be similarly tabulated by treatment group, categories of complaints or findings, severity, and duration.

Comparison of graded severities for local and systemic reactogenicity: Comparing one group with another during a period (the cross-over design has two periods), these are two-sample, cross-sectional comparisons of binary, ordinal, or continuous data. Comparing a group with itself between periods, these are paired, repeated measures data. Two-sample, cross-sectional comparisons are based on Fisher’s exact and Cochran-Mantel-Haenszel tests for binary and ordinal outcomes, respectively. Student’s-t and Wilcoxon tests are used for continuous outcomes. These methods permit statistical tests to address the following topics:

- 1. comparison of active and placebo after test article dose 1
  2. comparison of active and placebo after test article dose 2, with and without testing for the impact of, and adjustment for, outcomes after dose 1
  3. comparison of active and placebo in successive test article doses
  4. comparison of placebo in successive test article doses
  5. comparison of change from active to active with change from placebo to placebo

Items c. and d. are paired comparisons, which may be performed by McNemar tests or, more flexibly, by General Estimating Equation (GEE) models with a binary or continuous outcome. Items b. and e. are tests of interaction: dose 1 by dose 2 in item b., and period by vaccine in item e. and also accessible by GEE models.

### 9.2.3 Adverse Events:

Adverse events will be tabulated by body system using the COSTART dictionary, by severity, by seriousness, by relationship to study drug, and by elapsed time since last exposure to study drug. This yields primarily binary data (for each subject, the given COSTART event did or did not occur) which may be analyzed in the same manner as the binary reactogenicity events. Since multiple comparison corrections will not be used in this safety analysis, this analysis is vulnerable to grossly elevated type I error.

### 9.2.4 Vital Signs and Clinical Laboratory Measures:

Mean values for each measure will be plotted separately by treatment group, including ± one standard deviation. Extreme values and/or outliers will be discussed individually. For clinical laboratory measures within cross-over groups, quantitative outcomes at baseline and day 28 will be compared by paired Student’s t-tests and Wilcoxon signed rank tests. Binary outcomes will be compared by McNemar’s tests. This analysis is also vulnerable to elevated type I error.

## 9.3 Illness Definitions and Analysis for Challenge Component:

9.3.1 Fever:

Fever will be defined as any oral temperature ≥ 37.9°C which is confirmed by a repeat observation at an interval of not less than 20 minutes.

9.3.2 Upper Respiratory Illness:

A subject will be considered to have upper respiratory illness if, on at least one observation in each of two (2) consecutive days, he/she reports:

- Rhinorrhea (runny nose) of grade 2 or greater, or
- Nasal congestion (stuffy nose) of grade 2 or greater, or
- Sore throat of grade 2 or greater.

And/Or, on at least two (2) consecutive days, he/she has physical findings of:

- Nasal discharge, or
- Pharyngitis, or
- Otitis, or
- Sinusitis

9.3.3 Lower Respiratory Illness:

A subject will be considered to have lower respiratory illness if, on at least one observation in each of two (2) consecutive days, he/she reports:

- Cough of grade 2 or greater

And/Or, on at least two (2) consecutive days, he/she has physical findings of:

- Râles, rhonchi, or wheezing which were not present on the day of challenge.

9.3.4 Systemic Illness:

A subject will be considered to have systemic illness if, on at least one observation in each of two (2) consecutive days, he/she reports:

- Headache of grade 2 or greater
- Myalgia or athralgia of grade 2 or greater

9.3.5 Illness (any):

A subject will be considered to have illness if he/she fulfills the criteria for fever, or upper respiratory illness, or lower respiratory illness, or systemic illness or any combination thereof.

9.3.6 Illness Score:

Illness score will be calculated for each subject as the sum of the following over study days 42 to 49:

- One (1) point for each 24 hour period in which fever is observed
- One (1) point for each 24 hour period in which one or more criteria for upper respiratory illness is present
- One (1) point for each 24 hour period in which one or more of the criteria for lower respiratory illness is present
- One (1) point for each 24 hour period in which the criterion for systemic illness is present

9.3.7 Infection:

A subject will be deemed to be infected if he/she has any one of:

- Influenza virus shedding in nasal wash at any level, or
- Influenza virus detected in nasal wash or throat swab specimens by PCR on more than one day, or
- A ≥ 4-fold increase in serum A/Panama/2007/99 HAI antibody titer between day 40 (± 3) and day 60 (± 3) and absent clinical disease suggesting influenza between discharge from the isolation unit and the day 60 visit.

9.3.8 Analysis:

9.3.8.1 Power Considerations:

The analysis will consider both binary outcomes (e.g., illness vs. no illness; virus shedding vs. no virus shedding, etc.) and continuous outcomes (e.g., illness score, peak viral shedding and shedding AUC, etc.). The variability, and potential magnitudes of the differences in the latter are *a priori* unknown. However, the virus challenge inoculum will be selected to yield at least 60% (but less than 100%) of the binary outcome “any illness.” (Closer control of this rate is difficult to ensure.) Assuming that a minimal efficacy level of 70% (where efficacy = [rate _controls_ -rate_treated_ ] x 100 / rate _controls_) is of interest to justify further development of a given dose or formulation, it is possible to calculate that a group size of at least 20 will yield ≥ 0.80 power to detect, with a two-tailed α error of 0.05, a ≥ 70% reduction in the rate of the binary outcome “any illness” when the rate of “any illness” in controls is ≥ 60%.

9.3.8.2 Analyses:

The analysis concerns between-group comparisons of the three cross-over groups regarding clinical and immunologic binary outcomes. Alternative definitions provide six binary clinical outcomes (fever, upper respiratory illness, lower respiratory illness, systemic illness, illness [any], and illness + infection) that can be compared between groups by chi-square tests. More flexible analyses use logistic regression (SAS PROC LOGISTIC) with the presence or absence of influenza, separately for each definition, as the outcome. Covariates are cross-over study group, age, gender, race, and, for some models, titers after vaccinations 1 and 2.

Additional analyses will be based on illness score and on virus shedding assessed by two methods: peak and a time-weighted average calculated as a trapezoidal-rule AUC. For both peak and AUC, viral shedding is in units of log_10_ (titer). These continuous data will be compared between cross-over groups by Student’s t-tests and Wilcoxon tests and, in a more flexible analysis, by linear model adjusted for the demographic and clinical covariates cited above.

Finally, the three binary measures of influenza and the two viral load estimates (peak titer and AUC) will be assessed relative to the two immunologic measures, HAI reciprocal titer and nasal secretory IgA. For the binary measures, mean viral load (for each measure) is compared by t-tests between those with and without influenza (for each definition). For the two immunologic measures, the test of association with viral load will be based on Pearson correlations of log-titers.

# 10.0 References:

1. Betts RF, Treanor JJ. 2000. Approaches to improved influenza vaccination. Vaccine 18:1690-5.

2. Glezen WP, Taber LH, Frank A, *et al*. 1997. Influenza virus infection in infants. Pediatr Infect Dis J 16:1065-8.

3. Webster RG. 2000. Immunity to influenza in the elderly. Vaccine 18:1686-9.

4. Monto AS, Davenport FM, Napier JA, *et al*. 1969. Effect of vaccination of a school-age population upon the course of an A2-Hong Kong influenza epidemic. Bull World Health Org 41:537-42.

1. Demicheli V, Jefferson T, Rivetti D, *et al*. 2000. Prevention and early treatment of influenza in healthy adults. Vaccine 18:957-1030.

6. Clements ML, Betts RF, Tierney EL, *et al*. 1986. Serum and nasal wash antibodies associated with resistance to experimental challenge with influenza A wild-type virus. J Clin Microbiol 24:157-60.

7. Clements ML, Murphy BR. 1986. Development and persistence of local and systemic antibody response in adults given live attentuated or inactivated influenza A virus vaccine. J Clin Microbiol 23:66-72.

1. Nichol KL, Margolis KL, Wuorenma J, *et al*. 1994. The efficacy and cost-effectiveness of vaccination against influenza among elderly persons living in the community. N Eng J Med 331:778-84.
2. Gross PA, Hermogenes AW, Sacks HA, et al. 1995. The efficacy of influenza vaccine in elderly persons. Ann Intern Med 123:518-27.

10. Murasko DM, Goonewardene IM. 1990. T-cell function in aging: mechanisms of decline. Ann Rev Gerontol Geriatr 10:71-96.

11. Lamm ME, Robinson JK, Kaetzel CS. 1992. Transport of IgA immune complexes across epithelial membranes: new concepts in mucosal immunity. Adv Exp Med Biol 327:91-4.

12. Mazanec MB, Kaetzel CS, Lamm ME, *et al*. 1992. Intracellular neutralization of virus by immunoglobulin A antibodies. Proc Natl Acad Sci USA 89:6901-5.

13. Lamm ME. 1998. Current concepts in mucosal immunity. IV. How epithelial transport of IgA antibodies relates to host defense. Am J Physiol 274:614-7.

14. Boyce TG, Gruber WC, Coleman-Dockery SD, *et al*. 2000. Mucosal immune response to trivalent live attenuated intranasal influenza vaccine in children. Vaccine 18:82-8.

15. Belshe RB, Mendelman PM, Treanor J, *et al*. 1998. The efficacy of live attenuated, cold-adapted, trivalent intranasal influenza virus vaccine in children. New Eng J Med 338: 1405-12.

16. Treanor JJ, Kotloff K, Betts RF, *et al*. 2000. Evaluation of trivalent, live, cold-adapted (CAIV-T) and inactivated (TIV) influenza vaccines in prevention of virus infection and illness following challenge of adults with wild-type influenza A (H1N1), A (H3N2) and B viruses. Vaccine 18:899-906.

17. Nichol KL, Mendelman PM, Mallon KP *et al*. 1999. Effectiveness of live, attenuated intranasal influenza virus vaccine in healthy working adults: a randomized clinical trial. JAMA 282:137-44.

18. Powers DC, Fries LF, Murphy BR, *et al*. 1991. In elderly persons live attenuated influenza A virus vaccines do not offer an advantage over inactivated virus vaccine in inducing serum or secretory antibodies or local immunologic memory. J Clin Microbiol 29:498-505.

19. Beyer WEP, Palache AM, deJong JC, Osterhaus ADME. 2002. Cold-adapted live influenza vaccine versus inactivated vaccine: systemic vaccine reactions, local and systemic antibody response, and vaccine efficacy. A meta-analysis. Vaccine 20:1340-53.

20. Waldman RH, Bond JO, Levitt LP, *et al*. 1969. An evaluation of influenza immunization; influence of route of administration and vaccine strain. Bull World Health Org 41:543-8.

1. Waldman RH, Mann JJ, Small PA. 1970. Immunization against influenza. JAMA 207:520-4.
2. Waldman RH, Wood SH, Torres EJ, Small PA. 1970. Influenza antibody response following aerosol administration of inactivated virus. Am J Epidemiol 91:575-84.
3. Liem KS, Jacobs J, Marcus EA, van Strik R. 1973. The protective effect of intranasal immunization with inactivated influenza virus vaccine. Postgrad Med J 49:175-9.
4. Fukumi H. Experience of nasal application of inactivated influenza vaccine. Develop Biol Stand 33:155-61.
5. Oh Y, Ohta K, Kuno-Sakai H, *et al*. 1992. Local and systemic influenza haemagglutinin-specific antibody responses following aerosol and subcutaneous administration of inactivated split influenza vaccine. Vaccine 10:506-11.
6. Muszkat M, Friedman G, Schein MH, *et al*. 2000. Local SIgA response following administration of a novel intranasal inactivated influenza virus vaccine in community residing elderly. Vaccine 18:1696-9.
7. Muszkat M, Ben Yehuda A, Schein MH, *et al*. 2000. Local and systemic immune response in community-dwelling elderly after intranasal or intramuscular immunization with inactivated influenza vaccine. J Med Virol 61:100-6.
8. Kuno-Sakai H, Kimura M, Ohta K, *et al*. 1994. Developments in mucosal influenza virus vaccines. Vaccine 12 (14);1303-10.
9. Wilschut J, de Haan A, Geerligs HJ, *et al*. 1994. Liposomes as a mucosal adjuvant system: an intranasal liposomal influenza subunit vaccine and the role of IgA in nasal anti-influenza immunity. J Liposome Res 4:301-14.
10. Glück R, Mischler R, Durrer, et al. 2000. Safety and immunogenicity of intranasally administered inactivated trivalent virosome-formulated influenza vaccine containing Escherichia coli heat-labile toxin as a mucosal adjuvant. J Infect Dis 181:1129-32.
11. Bourguignon P, Bisteau M, Veenstra S, et al. 2001. Reactogenicity and passage into the brain of enterotoxins and CPG-oligonucleotides administered intranasally to mice. Abstracts of the Fourth Annual Conference on Vaccine Research, Arlington VA, 23 - 25 April, 2001; p. 53.
12. Lowell GH. 1997. Proteosomes for improved nasal, oral or injectable vaccines. *In:* Levine MM, Woodrow GC, Kaper JB, Cobon GS (ed.), New Generation Vaccines, 2^nd^ ed. Marcel Dekker, New York, p. 193-206.
13. ElGuink N, Kris RM, Goodman-Snitkoff G, *et al*. 1989. Intranasal immunization with proteoliposomes protects against influenza. Vaccine 7:147-151
14. Levi R, Aboud-Pirak E, LeClerc C, *et al*. 1995. Intranasal immunization of mice against influenza with synthetic peptides anchored to proteosomes. Vaccine 13:1353-9.
15. Fries LF, Montemarano AD, Mallett CP, et al. 2001. Safety and immunogenicity of a proteosome-*Shigella flexneri* 2a lipopolysaccharide vaccine administered intranasally to healthy adults. Infect Immun 69: 4545-53.
16. Plante M, Jones T, Allard F, Torossian K, *et al*. 2002. Nasal immunizations with subunit proteosome influenza vaccines induces serum HAI, mucosal IgA, and protection against influenza challenge. Vaccine 20:218-25.
17. Treanor J, Burt D, Lowell G, et al. 2001. Phase I evaluation of an intranasal proteosome-influenza vaccine in healthy adults. Abstracts of the Fourth Annual Conference on Vaccine Research, Arlington VA, 23 - 25 April, 2001; p. 55.
18. Fries L, Treanor J, Burt D, et al. 2001. Safety and Immunogenicity of one- and two-dose regimens of proteosome-monovalent influenza vaccine given intranasally to healthy young adults. IVth International Symposium on Viral Respiratory Infections, Willemstadt, Curaçao, 29 Nov – 02 Dec, 2001.
19. Clements ML, Subbarao EK, Fries LF, et al. 1992. Use of single-gene reassoratant viruses to study the role of avian influenza A virus genes in attenuation of wild type human influenza A virus for squirrel monkeys and adult human volunteers. J Clin Microbiol 30:655-62.
20. Fries LF, Dillon SB, Hildreth JEK, *et al*. 1993. Safety and immunogenicity of a recombinant protein influenza A vaccine in adult human volunteers, and protective efficacy against wild-type H1N1 virus challenge. J Infect Dis 167:593-601.
21. Clements ML, Betts RF, Tierney EL, Murphy BR. 1986. Resistance of adults to challenge with influenza A wild-type virus after receiving live or inactivated virus vaccine. J Clin Microbiol 23:73-6.
22. Treanor JJ, Kotloff K, Bets RF, et al. 2000. Evaluation of trivalent, live cold-adapted (CAIV-T) and inactivated (TIV) influenza vaccines in prevention of virus infection and illness following challenge of adults with wild-type influenza A (H1N1), A (H3N2), and B viruses. Vaccine 18:899-906.
23. Clark A, Potter CW, Jennings R, et al. 1983. A comparison of live and inactivated influenza A (H1N1) virus vaccines 1. Short term immunity. J Hyg Cambridge 90:351-9.
24. Hayden FG, Treanor JJ, Betts RF, et al. 1996. Safety and efficacy of the neuraminidase inhibitor GG167 in experimental human influenza. JAMA 275:295-9.
25. Calfee DP, Peng AW, Cass LM, et al. 1999. Safety and efficacy of intravenous zanamavir in preventing experimental human influenza A virus infection. Antimicrob Agents Chemother 43:1616-20.
26. Zeger SL, Liang K-Y. 1986. Longitudinal data analysis for discrete and continuous outcomes, Biometrics 42:121-30.

# Appendix A. Proteosome-Trivalent Influenza

# Vaccine Dose Preparation Worksheet

Date: ___________________ (dd/mm/yy)

Lot no. of proteosome-trivalent influenza vaccine: 0964

Mean Influenza hemagglutinin (HA) concentration (per strain) 170 μg / mL

Step 1. Estimate required volume of vaccine required at each dose level today:

| For 30 μg dose: | ______________ | X | 1.0 mL | = | ________ mL (volume B) |
| --- | --- | --- | --- | --- | --- |
|  | No. of subjects to receive 30 μg dose |  |  |  |  |
|  |  |  |  |  |  |

Step 2. Calculate number of stock vaccine vials needed today:

|  |  | |  |  |  |  |
| --- | --- | --- | --- | --- | --- | --- |
| For 30 μg dose: | _____ mL | | X | 0.63 | = | _______mL (volume E) |
|  | (volume B) | |  |  |  |  |
|  |  | |  |  |  |  |
| Number of vials | = | volume E | | | ÷ | 0.9 |
|  |  | |  |  |  |  |

Step 3. Remove requisite number of stock vaccine vials (PINK label) from refrigeration. Obtain sufficient vials of diluent/placebo (WHITE label). Diluent/placebo vials contain 10 mL of diluent; enough in one vial to yield ten (10) placebo doses or provide diluent for approximately 25 active doses. Obtain vaccine dilution vials.

Step 4. Prepare a nasal spray pump reservoir vial labeled with the ID no. of each subject to be dosed.

Step 5. Label vaccine dilution vials (vials and labels provided by sponsor) for the active and placebo test articles.

Initial this page: ___________

Vaccine Dose Preparation Worksheet, page 2.

Step 6. Swirl stock vaccine vial(s) before withdrawing vaccine to ensure complete mixing. Prepare the final vaccine dilution and placebo by adding the following volumes to the vaccine dilution vial(s), using the calculations performed on page 1 of this worksheet.

|  |  | Stock Vaccine |  | Placebo/Diluent |
| --- | --- | --- | --- | --- |
|  |  |  |  |  |
| 30 μg active |  | _____ mL | + | ______ mL |
|  |  | Volume E |  | (Volume B – Volume E) |
|  |  |  |  |  |
| Placebo |  | 0 | + | ______ mL |
|  |  |  |  | (number of placebo subjects today X 1.0 mL) |

Swirl each dilution vial gently to ensure complete mixing.

Step 7. Referring to the treatment assignment list, determine the appropriate test article for each subject and place 0.9 mL of active vaccine dilution or placebo in the reservoir vial of the nasal spray device. Assemble the nasal spray pump onto the reservoir vial. Enter the required information for the subject in question into the Dose Preparation Log.

Step 8. Remove the nosepiece cover and then the safety collar from the nasal spray pump. Hold the assembled pump upright and fully depress the actuator three (3) times to prime the pump. Replace the safety collar, then the nosepiece cover. Deliver the pump to the clinic for test article administration.

Step 9. All stock vaccine and placebo vials and all vaccine dilution vials should be recapped and retained in a separate, secure location (which may be at room temperature) for return to the sponsor. NO VIAL THAT HAS BEEN PREVIOUSLY ENTERED SHOULD BE RE-USED.

Sign and date this form: ______________________________ _____________

Signature Date

# Appendix B. Table B.1

IDB-13004 Time and Events Schedule: Immunization Component

| **Day** | **-90 to**  **-7** | **-42 to - 5** | **-14 to -2** | **0** | **1** | **2** | **3** | **4** | **5** | **6** | **7** | **12** | **14** | **15** | **16** | **17** | **18** | **19** | **20** | **21** | **28** |
| --- | --- | --- | --- | --- | --- | --- | --- | --- | --- | --- | --- | --- | --- | --- | --- | --- | --- | --- | --- | --- | --- |
| **Screening consent** | x |  |  |  |  |  |  |  |  |  |  |  |  |  |  |  |  |  |  |  |  |
| **Medical history** | x |  |  | x |  |  |  |  |  |  |  |  | x |  |  |  |  |  |  |  |  |
| **A/Panama HAI titer** | x |  |  |  |  |  |  |  |  |  |  |  |  |  |  |  |  |  |  |  |  |
| **Clinical Trial Consent** |  | x |  |  |  |  |  |  |  |  |  |  |  |  |  |  |  |  |  |  |  |
| **ECG** |  | x |  |  |  |  |  |  |  |  |  |  |  |  |  |  |  |  |  |  |  |
| **Clinical hematology/chem.** |  | x |  |  |  |  |  |  |  |  |  |  |  |  |  |  |  |  |  |  | x |
| **Urinalysis** |  | x |  |  |  |  |  |  |  |  |  |  |  |  |  |  |  |  |  |  | x |
| **HIV serology** |  | x |  |  |  |  |  |  |  |  |  |  |  |  |  |  |  |  |  |  |  |
| **Nasal wash for spec. IgA** |  | x | x |  |  |  |  |  |  |  |  | x |  |  |  |  |  |  |  |  | x |
| **Serum for HAI antibody** |  |  | x |  |  |  |  |  |  |  |  | x |  |  |  |  |  |  |  |  | x |
| **Blood for cytokine studies** |  |  | x |  |  |  |  |  |  |  |  |  |  |  |  |  |  |  |  |  | x |
| **β-HCG (females)** |  |  |  | x |  |  |  |  |  |  |  |  | x |  |  |  |  |  |  |  |  |
| **Physical exam (complete)** |  |  | x | |  |  |  |  |  |  |  |  |  |  |  |  |  |  |  |  |  |
| **Vital signs** |  | x | x | |  | x |  |  |  |  |  | x | x |  | x |  |  |  |  |  |  |
| **Brief directed examination** |  |  |  | x |  | x |  |  |  |  |  |  | x |  | x |  |  |  |  |  |  |
| **Test article dose** |  |  |  | **X** |  |  |  |  |  |  |  |  | **X** |  |  |  |  |  |  |  |  |
| **Immed. complaint quest.** |  |  |  | x |  |  |  |  |  |  |  |  | x |  |  |  |  |  |  |  |  |
| **Memory Aid** |  |  |  | x | x | x | x | x | x | x |  |  | x | x | x | x | x | x | x |  |  |
| **Telephone contact** |  |  |  |  |  |  |  |  |  |  | x |  |  |  |  |  |  |  |  | x |  |
| **AEs/Con. meds.** |  |  |  | x |  | x |  |  |  |  | x | x | x |  | x |  |  |  |  | x | x |

**Note: Study days following day 0 represent the nominal study schedule. Acceptable variability at each time point is indicated in protocol text.**

# Appendix B. Table B.2

IDB-13004 Time and Events Schedule: Challenge Component

| **Day** | **40** | **41** | **42** | **43** | **44** | **45** | **46** | **47** | **48** | **49** | **50** | **51** | **52** | **60** |
| --- | --- | --- | --- | --- | --- | --- | --- | --- | --- | --- | --- | --- | --- | --- |
| **Admit to isolation** | x |  |  |  |  |  |  |  |  |  |  |  |  |  |
| **Interval medical history** | x |  |  |  |  |  |  |  |  |  |  |  |  |  |
| **ECG** | x |  |  |  |  |  |  |  |  | x |  |  |  | x |
| **Nasal wash for spec. IgA** | x |  |  |  |  |  |  |  |  |  |  |  |  | x |
| **Serum for HAI antibody** | x |  |  |  |  |  |  |  |  |  |  |  |  | x |
| **Blood for cytokine studies** |  |  |  |  |  |  |  |  |  |  |  |  |  | x |
| **Vital signs** | x | x | x | x | x | x | x | x | x | x |  |  |  | x |
| **Physical ex. (complete)** | x |  |  |  |  |  |  |  |  | x |  |  |  | x |
| **Symptom Diary Card** | x | x | x | x | x | x | x | x | x | x |  |  |  |  |
| **β-HCG (females)** |  |  | x |  |  |  |  |  |  |  |  |  |  |  |
| **Throat swab** |  |  | x | x | x | x | x | x | x | x |  |  |  |  |
| **Challenge inoculum** |  |  | **X** |  |  |  |  |  |  |  |  |  |  |  |
| **Directed physical examination** |  |  | x | x | x | x | x | x | x | x |  |  |  |  |
| **Tympanometry** |  |  | x | x | x | x | x | x | x | x |  |  |  |  |
| **Nasal wash for virus titration** |  |  |  | x | x | x | x | x | x | x |  |  |  |  |
| **Neuraminidase inhibitor rx.** |  |  |  |  |  |  |  |  | x | x | x | x | x |  |
| **Discharge from isolation** |  |  |  |  |  |  |  |  |  | x |  |  |  |  |
| **AEs/Con. meds.** | x | x | x | x | x | x | x | x | x | x |  |  |  | x |

**Note: Study days represent the nominal study schedule. Acceptable variability at each time point is indicated in protocol text.**

# Appendix C. Brief Examination Worksheet (Post-Immunization)

Study IDB-13004 Subject ID:_____________ Date:________ Observer’s Initials ______________

Study Day: 0 2 or 3 14 16 or 17

For each category of observation, place an “X” in the box most descriptive of the observations made.

|  | **Grade 0** | **Grade 1** | **Grade 2** | **Grade 3** |
| --- | --- | --- | --- | --- |
| **Nasal mucosal inflammation** | None | Erythema or edema | Erythema and edema | Ulceration |
| **Nasal discharge** | None | Clear, serous, scant | Purulent | Bloody ± purulent |
| **Pharyngeal inflammation** | None | Mild or patchy erythema | Severe erythema | Purulent exudate |
| **Sinusitis** | None |  | Mild tenderness | Severe tenderness or overlying erythema |
| **Cervical/ post-auricular nodes** | None | Minimal enlargement, firm, nontender | Moderate enlargement, firm, slight tenderness | V. enlarged, soft/fluctuant, severe tenderness |
| Otic inflammation | None | Dull tympanic membrane | Injected tympanic membrane | Retracted or bulging tympanic membrane, fluid |

# Appendix D. Prototype Memory Aid Page

**SID #:** __________  **Initials: ___ ____ ____**

|  | Study Day X | | | | | | | |  | | | | | | | | | | | | | | | | |  |
| --- | --- | --- | --- | --- | --- | --- | --- | --- | --- | --- | --- | --- | --- | --- | --- | --- | --- | --- | --- | --- | --- | --- | --- | --- | --- | --- |
| Today’s date (day/month/year) |  | |  | | 200__ | | | |  | |  | | | |  | | | |  | | | | | | | |
| Evening oral temperature: | °C | | | | | | | |  | | | | | | | | | |  | | | | | | | |
|  | Grade (Mark the appropriate box) | | | | | | | |  | | | | | | | | | | | | | | | | |  |
|  | **0** | **1** | | **2** | | **3** | | |  | | | |  | | | |  | | | |  | | | | |  |
| Did you feel tired or have less energy than usual? |  |  | |  | |  | | |  | | | |  | | | |  | | | |  | | | | |  |
| Was your appetite poor? |  |  | |  | |  | | |  | | | |  | | | |  | | | |  | | | | |  |
| Did you have a headache? |  |  | |  | |  | | |  | | | |  | | | |  | | | |  | | | | |  |
| Did you have muscle or joint aches? |  |  | |  | |  | | |  | | | |  | | | |  | | | |  | | | | |  |
| Did you have a runny nose? |  |  | |  | |  | | |  | | | |  | | | |  | | | |  | | | | |  |
| Did you have a stuffy nose? |  |  | |  | |  | | |  | | | |  | | | |  | | | |  | | | | |  |
| Did your nose burn or itch? |  |  | |  | |  | | |  | | | | | | | | | | | | | | | | |  |
| Did you have bleeding from your nose? |  |  | |  | |  | | |  | | |  | |  | |  | |  | |  | | |  | |  | |
| Did you have red or puffy eyes? |  |  | |  | |  | | |  | | |  | |  | |  | |  | |  | | |  | |  | |
| Did you have any sneezing? |  |  | |  | |  | | |  | | |  | | | | | | | | | |  | | | | |
| Did you have a sore throat? |  |  | |  | |  | | |  | | |  | | | | | | | | | |  | | | | |
| Did you have a cough? |  |  | |  | |  | | |  | | |  | | | | | | | | | |  | | | | |
| Did you have shortness of breath or wheezing? |  |  | |  | |  | | |  | | | |  | | | |  | | | |  | | |  | | |
|  |  | | | | | | |  | |  | | |  | | | | | |  | | | | | | | |
| Did you take any medications on this day? | No | | | | | | Yes | | | | | | | | | | | |  | | | | | | | |
| *If yes,* list medication(s) below: | | | | | | | | | | | | | | | | | | |  | | | | | | | |
|  | | | | | | | | | | | | | | | | | | |  | | | | | | | |
|  | | | | | | | | | | | | | | | | | | |  | | | | | | | |
|  | | | | | | | | | | | | | | | | | | |  | | | | | | | |
|  | | | | | | | | | | | | | | | | | | |  | | | | | | | |
| Grade Definitions:  *Grade 0* = Not at all.  *Grade 1* = I noticed it, but it didn’t really interfere with any of my usual activities significantly.  *Grade 2* = I had it, and it was bad enough that I couldn’t do a significant part of my usual activities.  *Grade 3* = I had it, and it was bad enough that I was not able to do most of my usual activities, or I had to get prescription medicine from a doctor. | | | | | | | | | | | | | | | | | | |  | | | | | | | |

# Appendix E. Immediate Complaints Questionnaire

Please help us learn more about the vaccine in this study by telling us whether you had any of the following symptoms in the half hour immediately after your vaccine dose. Place an “X” in the column which describes the worst degree of each problem or symptom that you experienced. If you simply didn’t have a certain problem or symptom, put your “X” in the “NONE” column.

Your Subject ID Number:__________ Your initials: __________ Dose 1 2

|  | **NONE** | **MILD** | **MODERATE** | **SEVERE** |
| --- | --- | --- | --- | --- |
| You might say:  Symptoms: | “I didn’t have it at all.” | “I had it; but it wouldn’t stop me from doing anything.” | “I couldn’t have gone back to my usual activities if it lasted; but it went away.” | “It’s still bad enough that I can’t go back to my normal activities.” |
| Burning or stinging in the nose |  |  |  |  |
| Burning or stinging in the throat |  |  |  |  |
| Itching in the nose, throat, or eyes |  |  |  |  |
| Shortness of breath |  |  |  |  |
| Light-headedness or dizziness |  |  |  |  |
| A new or itchy skin rash you didn’t have before |  |  |  |  |
| Feverishness |  |  |  |  |

If you had another bothersome or worrisome symptom not listed here that started after getting the vaccine, *please tell the study doctor before you leave the clinic*. Appendix F. Influenza Symptom Diary Card

Appendix F. Influenza Symptom Diary card

Your Study ID Number: _______ Your Initials: ___ ___ ___

Today’s date: ____ (day) ______ (month) ______ (year) Morning Evening (tick one)

Place an “X” in the box in each symptom row that best describes your experience at the moment and since completing the last diary card. Grade your symptoms using the scale below. Use the space to the right to record any other symptoms you want to discuss with the doctor.

| **Grade:** | **0** | **1** | **2** | **3** | **Other symptoms:** |
| --- | --- | --- | --- | --- | --- |
| **SYMPTOM:** |  |  |  |  |
| **Runny nose** |  |  |  |  |
| **Stuffy nose** |  |  |  |  |
| **Sore throat** |  |  |  |  |
| **Cough** |  |  |  |  |
| **Headache** |  |  |  |  |
| **Muscle or joint aches** |  |  |  |  |

0 = None. I don’t have it.

1 = I can notice it, but it’s not too bothersome.

2 = It is quite bothersome to me; under ordinary circumstances it could keep me from work.

3 = It would definitely keep me from work. I would stay in bed and/or consult a doctor about it.

# Reviewed by: ________________________________ Date: ____________ Time: (24 hour clock) __________

# Appendix G. Challenge Physical Examination Worksheet

Subject ID Number: _______ Study Day: 42 43 44 45 46 47 48 49 (circle one)

Date: ____ (day) ______ (month) ______ (year) Time: (24 hour clock): __________ Initials: ____________

| **Oral temp. ______ º C Pulse ________ bpm** | **Absent** | | **Present** | | |
| --- | --- | --- | --- | --- | --- |
| **Blood pressure _______/ _______ mm Hg** |  |  | Mild | | Mod-sev. |
| **Finding:** |  |  | |  | |
| **Upper respiratory:** |  |  | |  | |
| **Nasal discharge (clear or purulent)** |  |  | |  | |
| **Otitis (inflamed tympanic membrane, new fluid)** |  |  | |  | |
| **Pharyngitis (erythema, exudate, tonsillar enlargement)** |  |  | |  | |
| **Sinus tenderness** |  |  | |  | |
| **Lower respiratory:** |  |  | |  | |
| **New râles, rhonchi, or wheezing** |  |  | |  | |

# Tympanometry done? Yes ____ No ____ If no, why? _______________________________

# _____________________________________________________________________________

# Additional Vital Signs:

# Time (24 hour clock): __________ Oral temp. _____ -ºC; Pulse ______ bpm; Blood pressure _____/_____ mm Hg

# Time (24 hour clock): __________ Oral temp. _____ -ºC; Pulse ______ bpm; Blood pressure _____/_____ mm Hg

# 
