# Supplementary material for: An Intranasal Proteosome-Adjuvanted Trivalent Influenza Vaccine Is Safe, Immunogenic & Efficacious in the Human Viral Influenza Challenge Model. Serum IgG & Mucosal IgA Are Important Correlates of Protection against Illness Associated with Infection
Source: PLoS One. 2016 Dec 22;11(12):e0163089. doi: 10.1371/journal.pone.0163089 (PMC5179046; doi:10.1371/journal.pone.0163089)
Supplement: S2 File — The study protocol, for study entitled IDB-13005 (DOC) [file pone.0163089.s002.doc]

STUDY TITLE: A Phase II, Randomized, Double-Blind, Placebo-Controlled Study of Three Regimens of FluINsure™ Proteosome-Trivalent Influenza Vaccine Delivered by the Intranasal Route to Healthy Young Adults, Followed by Intranasal Challenge with Virulent Influenza A Virus

Protocol No.: IDB-13005

Sponsor: ID Biomedical of Québec

7150 Frederick Banting, Suite 200

Ville St-Laurent, QC, H4S 2A1, Canada

Reg. File No.: **19896-0001-A-77383**

Principal Investigator: Dr. Robert Lambkin, BSc (Hons), MRPharmS, PhD

Medical Investigator: Dr. Colin Gelder, BSc (Hons), MB (Hons), PhD, MRCP

Study Site: Retroscreen Virology Ltd.

The Medical Building

Queen Mary, University of London

327 Mile End Road, London, E1 4NS, U.K.

Version / Date: **Version 3.1 / 24 June 2003**

Sponsor’s Study Director: Louis F. Fries, M.D.

Address as below

Clinical Monitor: Louis F. Fries, M.D.

ID Biomedical

UMBC Technology Center

1450 S. Rolling Rd., Baltimore, MD 21227, U.S.A.

(voice): 410-455-5610 (fax): 410-455-5606

(mobile): 443-253-5298

Do Not Implement This Protocol Unless Signed on the Following Page By The Study Director And All Investigators.

*This protocol is a confidential communication of ID Biomedical, and is prepared for the sole purpose of informing Investigators and their professional assistants who are participating in clinical trials of the Proteosome-Trivalent Influenza Vaccine*

Signature Page:

The Investigators and the Sponsor have discussed and agreed upon the content of this protocol. The Investigators agree to perform this investigation according to protocol and in conformance with cGCP, and to abide by this protocol except in the case of medical emergencies or where departures from the protocol are necessary in the interest of subject safety. In non-emergent situations, such departures will be undertaken only after consultation with the sponsor.

Principal Investigator: _______________________________________________

Dr. Robert Lambkin, BSc (Hons), MRPharmS, PhD Date

Medical Investigator: _________________________________________________

Dr. Colin Gelder, BSc (Hons), MB (Hons), PhD, MRCP Date

Co-Investigator : _______________________________________________

Professor John S. Oxford, BSc (Hons), PhD Date

Sponsor’s Study

Director: _______________________________________________

Louis F. Fries, M.D. Date

Contents:

[Study Synopsis 6](#__RefHeading___Toc44328593)

[List of Abbreviations 10](#__RefHeading___Toc44328594)

[1.0 Introduction and Background: 11](#__RefHeading___Toc44328595)

[2.0 Objectives: 16](#__RefHeading___Toc44328596)

[3.0 Study Administration: 16](#__RefHeading___Toc44328597)

[3.1 Ethical Review and Informed Consent: 16](#__RefHeading___Toc44328598)

[3.2 Record Keeping, Monitoring, and Record Retention: 16](#__RefHeading___Toc44328599)

[4.0 Study Population: 17](#__RefHeading___Toc44328600)

[4.1 Subject Number: 17](#__RefHeading___Toc44328601)

[4.2 Inclusion Criteria: 17](#__RefHeading___Toc44328602)

[4.3 Exclusion Criteria: 18](#__RefHeading___Toc44328603)

[5.0 Study Design: 19](#__RefHeading___Toc44328604)

[5.1 Immunization Phase: 19](#__RefHeading___Toc44328605)

[5.1.1 Treatments: 19](#__RefHeading___Toc44328606)

[5.1.2 Safety and Tolerability Evaluation: 20](#__RefHeading___Toc44328607)

[5.1.3 Immunogenicity Evaluation: 20](#__RefHeading___Toc44328608)

[5.2 Challenge Phase: 21](#__RefHeading___Toc44328609)

[6.0 Investigational Supplies: 22](#__RefHeading___Toc44328610)

[6.1 Study Drugs: 22](#__RefHeading___Toc44328611)

[6.2 Delivery System for Study Drugs: 22](#__RefHeading___Toc44328612)

[6.3 Storage Requirements for Study Drugs: 22](#__RefHeading___Toc44328613)

[6.4 Test Article Dose Preparation: 23](#__RefHeading___Toc44328614)

[6.4.1 Randomization / Immunization Phase Day 0: 23](#__RefHeading___Toc44328615)

[6.4.2 Preparation of Individual Test Article Doses: 23](#__RefHeading___Toc44328616)

[6.5 Test Article Dose Administration: 24](#__RefHeading___Toc44328617)

[6.6 Accountability for Study Drugs: 24](#__RefHeading___Toc44328618)

[6.7 Challenge Virus: 24](#__RefHeading___Toc44328619)

[6.8 Storage of Challenge Virus: 25](#__RefHeading___Toc44328620)

[6.9 Disposal of Challenge Virus: 25](#__RefHeading___Toc44328621)

[7.0 Procedures and Methods: 25](#__RefHeading___Toc44328622)

[7.1 Procedures: 25](#__RefHeading___Toc44328623)

[7.1.1 First Screening Visit (days - 150 to - 7): 25](#__RefHeading___Toc44328624)

[7.1.2 Second Screening Visit (days -90 to - 5): 25](#__RefHeading___Toc44328625)

[7.1.3 Immunization Phase Day 0, First Treatment: 26](#__RefHeading___Toc44328626)

[7.1.4 Immunization Phase Days 0 – 6 (0 to 6 days after the First Treatment) 27](#__RefHeading___Toc44328627)

[7.1.5 Immunization Phase Day 7 (± 1 day, Follow-up Visit One): 27](#__RefHeading___Toc44328628)

[7.1.6 Immunization Phase Day 14 (± 2 days; Second Treatment): 27](#__RefHeading___Toc44328629)

[7.1.7 Immunization Phase Days 14 to 20 (0 – 6 days after the Second Treatment) 28](#__RefHeading___Toc44328630)

[7.1.8 Immunization Phase Day 21 (± 2 days; Follow-up Visit Two): 29](#__RefHeading___Toc44328631)

[7.1.9 Immunization Phase Day 28 (± 3 days; Follow-up Visit Three): 29](#__RefHeading___Toc44328632)

[7.1.10 Challenge Phase Day 0 (39 ± 3 days after First Treatment; Admission to Quarantine): 29](#__RefHeading___Toc44328633)

[7.1.11 Challenge Phase Day 1: 30](#__RefHeading___Toc44328634)

[7.1.12 Challenge Phase Day 2, Morning (Day of Challenge): 30](#__RefHeading___Toc44328635)

[7.1.13 Challenge Phase Day 2 Afternoon to Challenge Phase Day 9 Morning.: 31](#__RefHeading___Toc44328636)

[7.1.14 Challenge Phase Day 9 p.m.: 32](#__RefHeading___Toc44328637)

[7.1.15 Challenge Phase Day 20 – 30 (56 – 72 days after First Treatment; Final Follow-up Visit) 32](#__RefHeading___Toc44328638)

[7.2 Laboratory Tests: 33](#__RefHeading___Toc44328639)

[7.2.1 Hematology: 33](#__RefHeading___Toc44328640)

[7.2.2 Clinical Chemistry: 33](#__RefHeading___Toc44328641)

[7.2.3 Clinical Laboratory Serologies: 33](#__RefHeading___Toc44328642)

[7.2.4 Urinalysis: 33](#__RefHeading___Toc44328643)

[7.2.5 Pregnancy Testing: 34](#__RefHeading___Toc44328644)

[7.2.6 Specific Immune Response Assays : 34](#__RefHeading___Toc44328645)

[7.2.7 Influenza Virus Detection: 34](#__RefHeading___Toc44328646)

[7.3 Other Measurements: 34](#__RefHeading___Toc44328647)

[7.3.1 History and Physical Examination: 34](#__RefHeading___Toc44328648)

[7.3.2 Brief, Directed Physical Examination after Treatments: 35](#__RefHeading___Toc44328649)

[7.3.3 Temperature Measurements During the Challenge Phase: 35](#__RefHeading___Toc44328650)

[7.3.4 Directed Physical Examination During the Challenge Phase: 35](#__RefHeading___Toc44328651)

[7.3.5 Concomitant Medications: 36](#__RefHeading___Toc44328652)

[7.4 Early Discontinuation: 36](#__RefHeading___Toc44328653)

[8.0 Immediate Complaints, Vaccine Reactogenicity, Findings of Influenza and Adverse Events: 36](#__RefHeading___Toc44328654)

[8.1 Immediate Complaints: 36](#__RefHeading___Toc44328655)

[8.2 Vaccine Reactogenicity: 37](#__RefHeading___Toc44328656)

[8.3 Symptoms and Findings of Influenza: 37](#__RefHeading___Toc44328657)

[8.4 Adverse Events: 37](#__RefHeading___Toc44328658)

[8.4.1 Double Reporting of Reactogenicity Findings/Complaints and Findings of Influenza as Adverse Events: 38](#__RefHeading___Toc44328659)

[8.5 Serious Adverse Events: 39](#__RefHeading___Toc44328660)

[9.0 Analysis Plan: 40](#__RefHeading___Toc44328661)

[9.1 Immunogenicity: 41](#__RefHeading___Toc44328662)

[9.2 Safety: 41](#__RefHeading___Toc44328663)

[9.2.1 Power and Detectable Effect Size: 41](#__RefHeading___Toc44328664)

[9.2.2 Immediate Complaints, Vaccine Reactogenicity Complaints, and Standardized Ear, Nose, and Throat Exams: 42](#__RefHeading___Toc44328665)

[9.2.3 Adverse Events: 43](#__RefHeading___Toc44328666)

[9.2.4 Vital Signs and Clinical Laboratory Measures: 43](#__RefHeading___Toc44328667)

[9.3 Illness Definitions and Analysis for Challenge Component: 44](#__RefHeading___Toc44328668)

[9.3.1 Fever: 44](#__RefHeading___Toc44328669)

[9.3.2 Upper Respiratory Illness: 44](#__RefHeading___Toc44328670)

[9.3.3 Lower Respiratory Illness: 45](#__RefHeading___Toc44328671)

[9.3.4 Systemic Illness: 45](#__RefHeading___Toc44328672)

[9.3.5 Illness (Any): 45](#__RefHeading___Toc44328673)

[9.3.6 Infection: 45](#__RefHeading___Toc44328674)

[9.3.7 Laboratory-confirmed Influenza Illness: 46](#__RefHeading___Toc44328675)

[9.3.8 Analysis: 46](#__RefHeading___Toc44328676)

[9.3.8.1 Power Considerations: 46](#__RefHeading___Toc44328677)

[9.3.8.2 Analyses: 46](#__RefHeading___Toc44328678)

[10.0 References: 48](#__RefHeading___Toc44328679)

[Appendix A. Proteosome-Trivalent Influenza Vaccine Dose Preparation Worksheet 52](#__RefHeading___Toc44328680)

[Appendix B. Table B.1 54](#__RefHeading___Toc44328681)

[Appendix B. Table B.2 55](#__RefHeading___Toc44328682)

[Appendix C. Brief Examination Worksheet (Post-Immunization) 56](#__RefHeading___Toc44328683)

[Appendix D. Prototype Memory Aid Page 57](#__RefHeading___Toc44328685)

[Appendix E. Immediate Complaints Questionnaire 58](#__RefHeading___Toc44328687)

[Appendix F. Influenza Symptom Diary Card 59](#__RefHeading___Toc44328688)

[Appendix G. Challenge Physical Examination Worksheet 60](#__RefHeading___Toc44328689)

# Study Synopsis

| Company: | ID Biomedical of Québec |
| --- | --- |
| Trial Number: | IDB - 13005 |
| Finished Product: | FluInsure™ Proteosome – Trivalent Influenza Vaccine |
| Active Ingredient(s): | Hemagglutinin (HA) from each of A/New Caledonia /20/99 (H1N1), A/Panama/2007/99 (H3N2), and B/Shangdong/7/97 viruses and meningococcal outer membrane proteins |
| Title of the Trial: | A Phase II Randomized, Double-blind, Placebo-Controlled Study of Three Regimens of FluINsure™ Proteosome-Trivalent Influenza Vaccine Delivered by the Intranasal Route to Healthy Young Adults, Followed by Intranasal Challenge with Virulent Influenza A Virus |
| Development Phase: | Phase II |
| Principal Investigator: | Dr. Robert Lambkin, BSc (Hons), MRPharmS, PhD |
| Co-Investigator: | Professor John Oxford, BSc (Hons), PhD |
| Medical Investigator: | Dr. Colin Gelder, BSc (Hons), MB (Hons), PhD, MRCP |
| Trial Centers: | Retroscreen Virology Ltd., London |
| Planned Trial Period: | Approximately three months to complete screening; two months to complete enrollment, treatment and detailed safety assessment; approximately nine (9) days of inpatient confinement for challenge, final follow-up 11 -19 days post discharge. |
| Objectives: | 1. To evaluate safety and tolerability of FluInsure™ when delivered intranasally in **three** different regimens to healthy adults, 18 – 50 y.o., selected for pre-existing susceptible immune status with regard to A/Panama//2007/99  2. To evaluate the magnitude of immune responses, in the serum and mucosal compartments, to FluInsure™ given in three different regimens.  3. To develop preliminary data regarding the protective efficacy of FluInsure™ by evaluating reduction in influenza-like illness and viral shedding following intranasal challenge with virulent A/Panama/2007/99 |
| Trial Design: | Immunization: Randomized, blinded, and placebo-controlled; featuring the following treatment groups:   1. 15 μg of each of the three HAs, IN x 2, (N = 28) 2. 30 μg of each of the three HAs, IN x 1, and buffered saline placebo IN x 1 (N = 28) 3. 30 μg of each of the three HAs, IN x 2 (N = 28) 4. Buffered saline placebo, IN x 2 (N = 28)   Challenge: Intranasal challenge of approximately 100 subjects randomly drawn from each of the above four treatment groups at 42 days (± 7) after the first test article dose. Challenge will be administered by nasal drops containing viable, egg-grown A/Panama/2007/99 |
| Planned Sample Size: | Up to 112 healthy adult volunteers treated, approximately 100 challenged |
| Inclusion Criteria: | 1. Age 18 to 50 years 2. Good general health status as determined by screening evaluation no greater than 42 days prior to immunization 3. Comprehension of the study requirements, including willingness to forego the licensed 2003 – 2004 intramuscular influenza vaccine until protocol participation is complete; expressed availability to fulfill the study requirements; signed informed consent 4. For female subjects, provision of a history of reliable contraceptive practices 5. Serum reciprocal hemagglutination inhibition titer for A/Panama/2007/99 of ≤10 at screening prior to the immunization phase |

| Exclusion Criteria: | 1. Presence of significant acute or chronic, uncontrolled medical or psychiatric illness; significant abnormalities in baseline serum chemistry, hematology, or urinalysis parameters 2. Inadequate venous access for study phlebotomies 3. Positive serologic test for HIV, or **evidence of illicit drug use by urine screening,** 4. In female subjects, a positive urine β-HCG on the day of any test article dose or virus challenge 5. Chronic use of any medication or other product, prescription or over-the-counter, for symptoms of rhinitis or nasal congestion, or any chronic nasopharyngeal complaint 6. Any history of asthma of any etiology in adulthood 7. Smokers unwilling/unable to desist for the inpatient component of the trial 8. Acute use of any medication or other product, prescription or over-the-counter, for symptoms of rhinitis or nasal congestion within seven (7) days prior to the first test article dose 9. Abnormal ECG 10. Any anatomic or neurologic abnormality impairing the gag reflex or conducive to aspiration, or history suggestive of such a problem 11. Receipt of systemic glucocorticoids (in a dose ≥ 5 mg prednisone daily or equivalent) within one month, or any other cytotoxic or immunosuppressive drug within six months 12. Receipt of any investigational drug within one month, or participation in a clinical trial of any influenza vaccine or any influenza challenge within one year 13. Presence of any febrile illness or significant symptoms of upper respiratory infection on the day of immunization or between admission for influenza challenge and administration of the challenge inoculum 14. History of hypersensitivity to mercurials or chicken eggs |
| --- | --- |
| Investigational Product: | FluINsure™ Proteosome Trivalent Influenza Vaccine:  Form: liquid (**0.9** mL per 3 mL vial)  Dose: either 15 μg or 30 μg of each of three (3) influenza hemagglutinins complexed to proteosomes, total volume administered will be 0.28 mL by nasal spray  Route: Intranasal  Lot number: 1066  Each vial contains:   1. 150 ± 30 μg / mL of hemagglutinin (HA) from *each* of A/New Caledonia/20/99 (H1N1), A/Panama/2007/99 (H3N2) and B/Shangdong/7/97 viruses, non-covalently bound to outer membrane proteins (OMP) of *Neisseria meningitidis* strain 8047 (OMP to HA ratio is approximately 4:1). 2. 10 mM Na/K phosphate-buffered isotonic saline, pH 7.4, with 0.01% thimerosal 3. Non-hemagglutinin viral proteins 4. Trace amounts of egg proteins |
| Control Product: | Phosphate buffered normal saline diluent / placebo:  Form: liquid (10 mL per 20 mL vial)  Dose volume: 0.28 mL by nasal spray  Each vial contains:  10 mM Na/K phosphate-buffered isotonic saline, pH 7.4, with 0.01% thimerosal |
| Challenge: | A/Panama/2007/99, lot I B 44/3, prepared in eggs in compliance with GMP, sterile and mycoplasma-free |
| Trial Time Lines: | Screening in March 2003. Immunization in June 2003. Challenge July 2003. |

| Study Schedule | Test article administrations will occur on immunization phase days 0 and 14 (± 1). Clinical evaluations will be performed at screening, before and after each test article dose, and at days 28 (± 3) and 39 (± 3) . Specimens for clinical chemistry, hematology and urinalysis will be collected at screening (prior to day 0) and on day 28 (± 2). Specimens for assessment of immune response will be collected at screening and on days 28 (± 2) and 40.  Subjects will be admitted to an inpatient challenge facility 39 ± 3 days after the first immunization and be observed for signs of spontaneous illness for 48 hours. On the morning of the second full challenge phase day, the intranasal challenge will be administered. Subjects will undergo temperature monitoring four times daily, provide twice-daily symptom reports based on a standardized questionnaire of influenza-like symptoms, and have a daily physician examination from the evening of the second challenge day through the afternoon-evening of the ninth challenge phase day. In addition, daily nasal washes and throat swabs will be performed to monitor virus shedding. On the morning of the eighth challenge phase day, subjects will begin treatment with a neuraminidase inhibitor to ensure virus clearance prior to discharge. A follow-up visit on the 20^th^ to 30^th^ challenge phase day will include a clinical evaluation and serum sampling for antibody responses to the challenge agent.  A detailed study schedule appears in appendix B. |
| --- | --- |
| Follow-up Duration: | Volunteers will be followed for 60-70 days after the day 0 immunization. |
| Assessment Methods: | 1. Standard clinical parameters for evaluating the safety of a biologic or vaccine product including: standardized diaries/questionnaires for local and systemic vaccine reactions, repeated vital signs and physical examinations, 60-70 day follow-up for adverse events and concomitant medication changes, monitoring of clinical chemistries (liver and renal function), urinalysis, and hematology.   2. Immunogenicity assessments will include serum hemagglutinin-inhibiting (HAI) antibody specific for the three viruses included in the vaccine, and levels of secretory IgA for these viruses present in nasal wash fluids. In addition, whole blood samples will be used to assess cellular cytokine responses to stimulation with influenza viral antigen.  3. Evaluation of challenge induced influenza will include repeated vital signs and examinations of the ears, nose, throat and lungs. In addition, a standardized symptom questionnaire will be administered twice daily. Once-daily nasal washes and throat swabs will be performed for **detection and / or quantitation of influenza virus using culture, antigen detection and / or molecular biology techniques.** |
| Statistical Methods:  Statistical Methods (cont’d.) | Demography: descriptive statistics will be provided.  Vaccine reactogenicity: Analyses concern graded severities for local and systemic reactogenicity: Comparing one group with another during a treatment period (the design has two periods following the first and second test article exposures), these are two-sample, cross-sectional comparisons of binary, ordinal, or continuous data. Comparing a group with itself between periods, these are paired, repeated measures data. Two-sample, cross-sectional comparisons are based on Fisher’s exact and Cochran-Mantel-Haenszel tests for binary and ordinal outcomes, respectively. Student’s-t and Wilcoxon tests are used for continuous outcomes. These methods permit statistical tests to address the following topics:   - 1. comparison of active and placebo after test article dose 1   2. comparison of active and placebo after test article dose 2, with and without testing for the impact of, and adjustment for, outcomes after dose 1   3. comparison of active and placebo in successive test article doses   4. comparison of placebo in successive test article doses   5. comparison of change from active to active with change from placebo to placebo   Items c. and d. are paired comparisons, which may be performed by McNemar tests or, more flexibly, by GEE models with a binary or continuous outcome. Items b. and e. are tests of interaction: dose 1 by dose 2 in item b., and period by vaccine in item e. and also accessible by GEE models.  Adverse events: Adverse events will be tabulated by body system using the COSTART dictionary, by severity, by seriousness, by relationship to study drug, and by elapsed time since last exposure to study drug. This yields primarily binary data (for each subject, the given COSTART event did or did not occur) which may be analyzed in the same manner as the binary reactogenicity events.  Vital signs & clinical laboratory measures: Mean values and S.D.s will be plotted separately by treatment group. Extreme values and/or outliers will be discussed individually. For clinical laboratory measures within cross-over groups, quantitative outcomes at baseline and day 28 will be compared by paired Student’s t-tests and Wilcoxon signed rank tests. Binary outcomes will be compared by McNemar’s tests.  Immunogenicity: Immunogenicity measures will include serum hemagglutination-inhibiting (HAI) antibody titers specific for the strains included in the vaccine, levels of secretory IgA (sIgA) specific for these viruses in nasal wash fluids. For the HAI titers, analyses will concern geometric mean titers (GMT), proportions of subjects with titer ≥ 40, and proportions with ≥ four-fold increase over baseline. For specific sIgA levels, analyses will concern geometric means and fold-rise from baseline. HAI GMTs and geometric mean nasal specific sIgA levels will be accompanied by 95% confidence intervals. Comparing active-to-placebo and active-to-active groups, titers/antibody levels will be contrasted on day 28 by t-tests on log_10_(titer). Within the groups, paired tests will be used (Student’s t and Wilcoxon signed rank). Fold-rises may be examined with and without covariate-adjustment for previous titers. Without such adjustment, there are two binary fold-rise outcomes, and these are compared within and between groups by chi-square and stratified chi-square tests. (In the latter case, 2 x 2 tables of treatment by outcome are stratified by previous titers.) With covariate adjustment, the fold-rise outcome is the log_10_-ratio of early and later titers. Adjustment after the first treatment is for pre-treatment titer and adjustment after the second treatment is for both baseline titer and for titers after the first treatment.  Response to viral challenge: The analysis concerns between-group comparisons of the three groups regarding clinical and immunologic binary outcomes. Alternative definitions provide six binary clinical outcomes that can be compared between groups by chi-square tests. More flexible analyses use logistic regression (with the presence or absence of illness, separately for each definition, as the outcome. Covariates are cross-over study group, age, gender, race, and, for some models, titers after vaccinations 1 and 2.  Additional analyses will be based on illness score and on virus shedding assessed by two methods: peak and a time-weighted average calculated as a trapezoidal-rule AUC. For both peak and AUC, viral shedding is in units of log_10_(titer). These continuous data will be compared between cross-over groups by Student’s t-tests and Wilcoxon tests and, in a more flexible analysis, by linear model adjusted for the demographic and clinical covariates cited above.  Finally, the three binary measures of influenza and the two viral load estimates (peak titer and AUC) will be assessed relative to the two immunologic measures, HAI reciprocal titer and nasal secretory IgA. For the binary measures, mean viral load (for each measure) is compared by t-tests between those with and without influenza (for each definition). For the two immunologic measures, the test of association with viral load will be based on Pearson correlations of log-titers. |

# List of Abbreviations

AE Adverse event

ALT Serum alanine amino transferase

AST Serum aspartate amino transferase

β- HCG *Beta* – human chorionic gonadotropin

COSTART Coding Symbols For Thesaurus Of Adverse Reaction Terms

CRF Case report form

ECG Electrocardiogram

ELISA Enzyme linked Immunosorbent Assay

ERC Ethical review committee

GCP Good Clinical Practice

HA (Influenza) Hemagglutinin antigen

HAI (Influenza) Hemagglutination Inhibiting (antibody)

HIV Human Immunodeficiency Virus

ICH International Conference on Harmonization

IgG Immunoglobulin G

Na / K Sodium / Potassium

LD_50_ 50% Lethal dose

OMP Outer Membrane Protein

RR Relative risk

SAE Serious Adverse Event

sIgA Secretory Immunoglobulin A

SID Subject Identification Number

# 1.0 Introduction and Background:

Influenza remains a major cause of morbidity and mortality in most areas of the world (1). Attack rates are highest in young children (especially infants 6 to 12 months of age [2]), but hospitalization and death are highest in the elderly and in patients with underlying metabolic, cardiovascular and pulmonary diseases. The average seasonal incidence of influenza-related death was reported to be 9.1 per 100,000 for the 1972-1992 period in the United States. In non-pandemic years, over 90% of deaths occur among persons 65 years of age or older (3). Although only a small proportion of influenza-related death occurs in the pediatric population, children have the highest morbidity rates and are also the major disseminators of the virus. Effective vaccination coverage of school-age children has resulted in a three-fold reduction in the overall rate of illness in all age groups (4).

Beneficial effects of the current inactivated, trivalent vaccines are most evident in healthy, young adults. A recent meta-analysis suggested that the frequently quoted estimate of 70% efficacy against serologically-confirmed influenza illness is borne out by the literature pertaining to the young adult age group (5). The virus-specific serum antibodies that are elicited in this group are pivotal elements in limiting or aborting spread of the virus and preventing illness, but are relatively ineffective at preventing infection *per se* or shortening viral shedding (1, 6, 7). In children, the current vaccine is immunogenic if two doses are given, but the requirement for two parenteral doses, repeated yearly, inhibits acceptance. In the elderly, more modest protection is achievable with the current vaccines, perhaps on the order of 40-60% (8, 9). This relative failure of protection in the elderly has been attributed to various factors, including lack of stimulation of a mucosal immune response (see below), and the senescence of T and B-cells and reduced IL-2 production (3, 10).

During natural infection influenza viruses gain entry through, and are essentially restricted to, respiratory mucosal surfaces. Consequently, mucosal immunity is a pivotal element in controlling these respiratory viral infections. The mucosal immune system consists of an integrated network of lymphoid cells working in concert with the innate mucosal barriers to promote host defense. The humoral arm of the mucosal immune system is comprises principally locally-synthesized polymeric IgA antibodies. Secretory IgA (sIgA) constitutes the majority (>80%) of all antibodies produced in mucosae-associated lymphoid tissues in humans and forms a first line of immune defense. The induction of sIgA antibodies has a key role in the *prevention* of mucosae-restricted respiratory infections. Secretory IgA functions in host defense have been demonstrated at three levels: a) in the lamina propria IgA antibodies bind antigens and cause them to be cleared into the lumen (11); b) antiviral IgA antibodies in transit through epithelial cells inhibit virus production by an intracellular action (12); and c) IgA antibodies secreted into the lumen can prevent antigens from adhering to and penetrating the epithelium (13). The mucosal immune response, as monitored by specific sIgA in the nose, has been shown to be a strong correlate of reduced infection rate, limited viral replication, and reduced illness in influenza challenge studies in adults (1, 6).

Although influenza immunization rates are increasing, the desired impact on morbidity and mortality has not been obtained. In order to address pediatric and elderly populations, mucosal immunization strategies, of which the most intensively investigated has been nasal immunization with live, cold-adapted reassortant viruses, have been developed. Assessment of the immunogenicity and efficacy of cold-adapted influenza vaccines have demonstrated that they can induce mucosal immune responses and show protective efficacy in children and in healthy young adult volunteers (5, 14 – 17). In naïve children, immune responses to these vaccines are vigorous in both the serum and mucosal compartments, whereas in seropositive children, and even sero-negative adults, serum responses are modest. In studies performed in the elderly, cold-adapted influenza vaccines have offered minimal or no advantage over inactivated virus vaccines in terms of serum or secretory antibody or local immunological memory induction in this group (18). A recent meta-analysis concluded that the efficacies of inactivated intramuscular and live, cold-adapted intranasal vaccines were similar, but included predominantly young adult data (19).

An alternative approach to intranasal influenza immunization is the use of inactivated vaccine antigens via the nose. Inactivated influenza antigens of various types, when given intranasally, have been safe in a large cumulative number of adults and children. They have induced significant mucosal and systemic immune responses, but crude, unfractionated virus and/or potentially uneconomic doses of more purified antigens were frequently required (20 - 28). Limited data suggest that inactivated influenza antigens given intranasally can have protective efficacy comparable to intramuscular vaccine (reviewed in 5, see also 20, 21, 23, 24). Lipid-based delivery systems have been reported to enhance influenza antigen immunogenicity via the nasal route (29). Native *E. coli* heat-labile toxin has also been shown to have potent nasal adjuvant properties for influenza antigens (30). However, this adjuvant has been shown to enter neural tissues and cause inflammatory pathology when given intranasally to rodents (31) – and may be associated with unacceptable safety concerns, such as facial nerve palsies, in humans.

Proteosomes are hydrophobic, proteinaceous nanoparticles comprising *Neisseria meningitidis* outer membrane proteins. Proteosomes can non-covalently associate with macromolecules containing hydrophobic domains and successfully present these macromolecules to the mucosal immune system (32). The proteosome mucosal vaccine delivery system is one of the limited number of

sub-unit approaches capable of eliciting mucosal and systemic responses *in vivo.* It is well-suited to the delivery of amphophilic viral membrane glycoproteins such as the influenza hemagglutinin and neuraminidase. Animal studies of mucosally-applied proteosome-formulated vaccines have demonstrated strong systemic and mucosal antigen-specific immune responses and protection against disease in several model systems using multiple classes of antigen, including influenza (32 - 34). Recently, intranasal administration to adult humans of proteosome-based vaccines against influenza or bacillary dysentery, the latter in doses containing up to 1.5 mg of proteosome protein, has proven safe, well-tolerated and immunogenic in both the systemic and mucosal compartments (35, and ID Biomedical unpublished data).

Murine studies of intranasally-delivered proteosome-influenza vaccines have shown induction of virus-specific serum IgG, hemagglutination-inhibiting, and virus-neutralizing antibodies in titers equivalent to those induced by classical inactivated intramuscular vaccine antigens given in the same dose, or by sub-lethal infection with homologous virus. Induction of statistically-significant virus-specific serum IgA titers (which in mice reflect mucosal production), nasal and pulmonary IgA titers was also observed. Neutralizing antibodies were observed in lung washes of mice that received intranasal proteosome-influenza vaccine, but not those of mice that received intramuscular vaccine. Mice immunized intranasally with proteosome-influenza vaccine showed solid protection against an intranasal challenge with 4 LD_50_ of homologous mouse-adapted virus. (36)

In humans, ID Biomedical has now carried out a total of six (6) clinical trials of proteosome-influenza vaccines given by the intranasal route (37 – 40). The first two (2) of these studies evaluated a monovalent prototype vaccine; the subsequent four (4) have utilized trivalent preparations including influenza A/H1N1 and A/H3N2 subtype antigens and influenza B antigens. These studies have evaluated a total of 387 healthy adult subjects of both genders and between the ages of 18 and 50; of these 304 have received active proteosome-influenza vaccine products and 83 have received either a saline placebo or another comparator. In the monovalent vaccine trials, intranasal doses containing between 7.5 and 45 μg of influenza virus hemagglutinin (HA) formulated with an approximate four-fold excess of proteosome proteins have been studied. In trivalent vaccine trials, doses containing between 15 and 45 μg of *each* of the three influenza virus HAs, also with an approximate four-fold excess of proteosome proteins, have been evaluated. Single-dose regimens and regimens providing two doses at a 14-day interval have been tested. Safety and immunogenicity results have been consistent between the monovalent and trivalent programs.

There have been no vaccine-attributable serious adverse events, and no ominous pattern of adverse events associated with a particular body system. Interestingly, several winter studies have demonstrated a suggestive, dose-related reduction in reports of “colds,” but no attempt has been made to correlate these reports with culture or serologically-confirmed influenza. In the immediate (30 minutes) post-dosing period, 10 – 15% of active vaccinees have reported a short-lived, mild nasal burning or stinging, but this has not prevented return to normal activities. There have been no hypersensitivity reactions or fevers. In the seven (7) days following vaccine doses, between 30 and 67% of vaccinees report mild nasal stuffiness, scant clear nasal discharge, or both (as compared to 20 – 30% of placebo recipients). In some, but not all, studies, transient sneezing is also associated with the active product. The overwhelming majority of these complaints have been very mild (grade 1, defined as “just noticeable”). These symptoms typically last one to three days and resolve spontaneously. Physical examination of the ears, nose, throat and cervical nodes has revealed minor erytema of the nasal mucosae and or scant rhinorrhea, but no erosive lesions. A few subjects have had mild tenderness over the paranasal sinuses, but the frequency of this finding after active doses does not clearly exceed that after placebo doses. There have been no fevers. No subject has refused a second dose based on his/her experience after the first dose. The frequency of vaccine reactogenicity complaints demonstrates a very shallow dose-response, and reactions after a second dose are not worse than those following the first.

Immunogenicity data have been consistent, particularly for the influenza A components. Between 40 and 60% of subjects entering the trials with serum hemagglutination inhibition (HAI) reciprocal titers < 40 for a given virus (a level typically associated with susceptibility) have demonstrated HAI titer rises of ≥ 4-fold and/or attained reciprocal titers ≥ 40. In unselected populations, HAI reciprocal titers ≥ 40, a four-fold rise, or both are seen in between 70 and 100% of subjects after immunization with any regimen containing at least 15 μg of influenza HA. Geometric mean nasal secretory IgA (sIgA) levels specific for the vaccine virus strains rise between two- and four-fold after immunization. Nasal sIgA responses tend to be stronger following two-dose regimens (3 to 4 –fold) than one-dose regimens (1.8 to 3-fold), but are statistically significant in almost all cases by repeated measures analysis of variance. These immune response levels are equal or superior to published responses to live, cold-adapted nasal influenza vaccines which have proven efficacious in the field.

As noted by Beyer *et al*., both increased serum HAI titers and specific nasal wash secretory IgA have been associated with protection against influenza (19). The relative importance of these factors in the induction of protection by non-living mucosal vaccines remains to be fully defined, rendering protective efficacy difficult to predict from immunogenicity data alone. Intranasal challenge of healthy young adults with egg-grown virulent influenza virus has been used extensively and safely in the United States and the United Kingdom to evaluate the immunogenicity and protective potential of a variety of live single-gene reassortant and subunit vaccine candidates (41, 42), to characterize the efficacy of the live, cold-adapted vaccine candidate and compare it to the intramuscular vaccine (43 – 45), and to test anti-viral compounds (46, 47).

Recently, ID Biomedical has collaborated with Retroscreen Virology limited to conduct a challenge study in subjects immunized intranasally with FluInsure™ or saline placebo. FluInsure™ regimens in this randomized, double-blinded study included either one or two intranasal doses of vaccine delivering 30 μg of each influenza HA (with an approximate four-fold excess of proteosome proteins). These subjects were subsequently challenged intranasally with egg-grown A/Panama/2007/99, the current A/H3N2 strain.

Safety and immunogenicity data continue to be analyzed, but are in general consistent with prior experience. Approximately 70% of both one- and two-dose vaccinees had signficant aserum HAI responses. A per-protocol analysis of the challenge study data revealed influenza-like illness in 66.7% of challenged placebo recipients, 62.5% of challenged one-dose recipients (6.3% efficacy), and 31.8% of challenged two-dose recipients (52.3% efficacy); yielding a chi^2^ p value of 0.041 for the 3 x 2 table and a p value of 0.021 for trend with number of vaccine doses. However, examination of the data has revealed that physician exam findings were non-contributory with one exception: pharyngitis; and that the definition of pharyngitis as applied by the study physicians led to fulfillment of the criteria for “illness” in a number of essentially asymptomatic subjects. In addition, the calibration of the disposable digital thermometers used to monitor temperature was questioned, as these yielded mean baseline temperatures just over 36ºC. Setting aside physician-observed pharyngitis and using a definition of fever based on the baseline mean + three standard deviations revealed a similar, but much stronger, efficacy trend in the data: 57.1% illness in placebo recipients; 45.8% illness in one-dose recipients (19.8% efficacy); and 4.5% illness (92.1% efficacy) in two-dose recipients (chi^2^ p value of 0.0007 for the 3 x 2 table and a p value of 0.0003 for trend with number of vaccine doses). An analysis based on laboratory-confirmed influenza illness (which requires illness and either isolation of influenza virus or a four-fold rise in reciprocal HAI titer across the challenge interval) demonstrates 65% efficacy in the one-dose group and 100% in the two-dose group. Because the sensitivity of influenza culture has proved poor for this strain however, this analysis remains preliminary pending data from molecular detection methods and immunoflouresence studies.

The current study is being undertaken to: a) confirm the efficacy of the two-dose regimen at a 30 μg dose level, b) re-examine the efficacy of the one-dose regimen at the 30 μg dose level with refined illness criteria, and c) examine the possible efficacy of a two-dose regimen at a 15 μg dose level, which is also highly immunogenic.

# 2.0 Objectives:

1. To assess the safety and tolerability of FluINsure™ when delivered intranasally to healthy adults 18 to 50 y.o. who are selected for susceptibility to A/Panama/2007/99. Regimens delivering two doses containing 15 µg of each of the three hemagglutinin (HA) antigens, or one or two doses containing 30 of each of the three (HA) antigens will be compared to a placebo treatment.

B. To assess the kinetics and magnitude of the immune responses to the above intranasal FluINsure™ regimens in healthy adults 18 to 50 y.o. who are selected for susceptibility to A/Panama/2007/99.

C. To evaluate the capacity of the three different regimens of FluINsure™ to provide protective immunity to previously-susceptible healthy young adults challenged with A/Panama/2007/99.

# 3.0 Study Administration:

## 3.1 Ethical Review and Informed Consent:

The clinical protocol will be conducted in conformance with the principles of the Declaration of Helsinki (Edinburgh, 2000), the International Ethical Guidelines for Biomedical Research Involving Human Patients, the ICH Consolidated Guideline for Good Clinical Practice, and all relevant local laws and regulations. The protocol and informed consent document will be reviewed and approved by a properly-constituted ethics committee and evidence of approval must be provided to ID Biomedical in writing prior to initiation of the trial. All aspects of the study will be explained in detail to prospective subjects and they will be informed of the voluntary nature of their participation. Written informed consent will be obtained from each subject using the approved documents, and each subject will receive a copy of his/her signed consents. Consent to screening will be obtained from each subject prior to obtaining an initial blood specimen for the sole purpose of determining sero-eligibility. Consent for participation in the immunization and challenge study will be obtained from sero-eligible subjects prior to any other study-related evaluations, procedures, or treatments.

## 3.2 Record Keeping, Monitoring, and Record Retention:

All required subject data will be recorded on case report forms (CRFs) or other study-specific media designed and provided by ID Biomedical (or equivalents reviewed and approved by ID Biomedical). CRFs will be completed in a timely manner and be kept available, along with supporting source documents, for periodic monitoring by an ID Biomedical representative. The Investigator is responsible for the accuracy of the data recorded on the CRFs. One copy of all CRFs and all documentation surrounding the trial must be securely retained by the Investigator for twenty-five (25) years.

# 4.0 Study Population:

## 4.1 Subject Number:

Approximately 112 healthy adults will be enrolled in the immunization portion of the study, i.e., 28 in each of four (4) treatment groups. Approximately one hundred of these will be randomly selected to participate in the challenge portion of the study (i.e., approximately 25 per treatment group).

## 4.2 Inclusion Criteria:

To be eligible for study, each subject will fulfill all of the following criteria:

• Age 18 to 50 years, inclusive,

• Comprehension of the study requirements, including willingness to forego the licensed 2003-2004 intramuscular influenza vaccine until completion of study participation; availability for the required study period, ability to attend scheduled study visits, and willingness to participate in the inpatient challenge,

- Willingness to provide written consent for participation after reading the Consent Form and after having adequate opportunity to discuss the study with an investigator or qualified deputy,
- Good general health status as determined by a screening evaluation no greater than 90 days prior to the first immunization,
- For female subjects, provision of a history of reliable contraceptive practices (hysterectomy or bilateral tubal ligation, oral or implanted contraceptive use, intrauterine device, barrier method plus spermicide, history of a single male partner with vasectomy, or a history of abstinence deemed credible by the investigator). *The provision of this history does NOT replace the requirement to perform, and obtain negative results in, pregnancy tests as per sections 4.3 and 7.2.5.*
- Reciprocal titer of serum hemagglutination-inhibiting (HAI) antibody to A/Panama/2007/99 (H3N2) ≤ 10.

## 4.3 Exclusion Criteria:

Subjects with any of the following will be excluded prior to enrollment in the immunization component of the study:

- Presence of significant acute or chronic, uncontrolled medical or psychiatric illness (subjects with uncomplicated chronic diagnoses stable and treated for ≥ three [3] months, e.g., mild hypertension well-controlled with medication, may be enrolled - provided the condition and its therapy are not known to be associated with an immunocompromised state or increased risk of complications of influenza),
- Venous access deemed inadequate for the phlebotomy demands of the study,
- Positive serologic test for HIV, **or evidence of illicit drug use by urine screening,**
- ALT or AST > twice the upper limit of normal; or any clinical laboratory value deemed by the investigator to indicate significant undiagnosed illness,
- Abnormal ECG,

• In female subjects, a positive urine β-HCG on the day of any test article dose, or on the day of challenge,

• Chronic use (*more than once a week in any two [2] of the four [4] weeks preceding the first test article dose*) of any medication or other product (prescription or over-the-counter), for symptoms of rhinitis or nasal congestion or any chronic nasopharyngeal complaint, or chronic use of *any* intranasal medication for any indication,

- Any history during adulthood of asthma of any etiology,
- Subjects who smoke and who are unwilling or unable to desist for the duration of the inpatient challenge component of the study,
- Acute use of any medication or other product, prescription or over-the-counter, for symptoms of rhinitis or nasal congestion within seven (7) days prior to the first test article dose,
- Any anatomic or neurologic abnormality impairing the gag reflex or associated with an increased risk of aspiration, or history suggestive of such a problem,

• Receipt of systemic glucocorticoids (in a dose ≥ 5 mg prednisone daily or equivalent) within one (1) month, or any other cytotoxic or immunosuppressive drug within six (6) months,

• Receipt of any investigational drug within one (1) month, or prior participation in a clinical trial of any influenza vaccine within one (1) year,

- Presence of any febrile illness or symptoms (greater than grade 1, “mild”) of upper respiratory infection on the day of first test article administration. (Such subjects may be re-evaluated for enrollment after resolution of the illness),
- Presence of any febrile illness or symptoms suggestive of influenza between admission for influenza challenge and administration of the challenge inoculum,
- History of hypersensitivity to mercurials (found in antiseptics such as merthiolate, some vaccines and skin test reagents, and contact lens solutions) or chicken eggs.

# 5.0 Study Design:

This is a randomized, double-blind, placebo-controlled phase II study of the safety, immunogenicity, and protective efficacy against challenge of FluINsure™ proteosome-trivalent influenza vaccine delivered as a nasal spray. The study has two (2) sequential components, as detailed below.

## 5.1 Immunization Phase:

FluINsure™ will be delivered as one of three different treatment regimens as detailed below in section 5.1.1. In addition, approximately one of every four subjects will receive a placebo control treatment regimen comprising two sequential doses of buffered saline placebo.

The study will enroll up to 112 healthy adult subjects selected for susceptibility to A/Panama/2007/99 into the immunization component.

Healthy normal subjects of both genders and 18 to 50 y.o. will be invited to give informed consent for preliminary screening phlebotomy, and then have a serum specimen obtained for baseline A/Panama/2007/99 HAI antibody testing. Screened subjects with serum reciprocal HAI titers ≤ 10 will be invited to return for a detailed explanation of the clinical trial and asked to give informed consent for participation in the trial itself. Consenting subjects will then undergo an assessment of their medical history, physical examination, ECG, and selected clinical laboratory tests to ensure eligibility according to the protocol. A nasal wash sample for measurement of secretory IgA (nasal sIgA) specific for the vaccine virus strains will be obtained during screening.

5.1.1 Treatments:

Eligible subjects will be randomized, using a gender-specific randomisation list prepared by the Sponsor, to one of four treatment groups in a ratio of approximately 1:1:1:1. The treatment groups will receive:

Treatment Group A) Two intranasal spray doses, each dose containing 15 μg of each of three hemagglutinins (HA) formulated with proteosomes. The first dose will be given on day 0 and the second given after a 14-day interval.

Treatment Group B) Two intranasal spray doses, the first containing 30 μg of each of three hemagglutinins (HA) formulated with proteosomes, and the second containing buffered saline (placebo). The first dose will be given on day 0 and the second given after a 14-day interval.

Treatment Group C) Two intranasal spray doses, each containing 30 μg of each of three hemagglutinins (HA) formulated with proteosomes. The first dose will be given on day 0 and the second given after a 14-day interval.

Treatment Group D) Two intranasal spray doses of buffered saline. The first dose will be given on day 0 and the second given after a 14-day interval.

All dose volumes will be 0.28 mL (as 0.14 mL per nostril). Each treatment group will include approximately 28 subjects.

5.1.2 Safety and Tolerability Evaluation:

Following each test article administration, each subject will maintain a written memory aid of potential vaccine reactogenicity for seven (7) days; and this data will be collected at an in-clinic interview at the end of the seven-day interval after each dose. Subjects who record complaints of severity grade 2 or greater at any time during the seven (7) days after each dose will return as soon as practicable to the clinic for a directed physician examination of the nose, throat, ears, and cervical nodes or any other implicated body system. Repeat clinical laboratory studies will be performed approximately 28 days after the first treatment (i.e., two weeks after the first exposure). Ascertainment of adverse events and changes in concomitant medications will be carried out through day 39 ± 3 following the first test article dose, and again on day 60 – 70 after the first exposure. Specific definitions and grading for vaccine reactogenicity and adverse events are outlined in section 8.2 and related appendices.

5.1.3 Immunogenicity Evaluation:

Venous blood and nasal wash specimens will be collected at specified intervals to examine the systemic and mucosal specific immune response. At day 39 ± 3 following the first test article administration, a physical examination, venous blood collection for antigen-specific serologies, and collection of nasal wash specimens will complete the immunization component of the study.

5.2 Challenge Phase:

At day 39 ± 3 following the first test article administration, all subjects who remain eligible and who consent will be admitted to a quarantine facility. Following admission, subjects will have oral temperature ascertained four times per day, and will be questioned twice daily regarding symptoms of influenza or upper respiratory infection using a standardized questionnaire. In addition, a directed physician examination of the ears, nose, throat, sinuses, and lungs will be performed daily following a standard worksheet. Any subject developing fever (oral temperature ≥ 37.9º C on two observations separated by at least 20 minutes) or complaints of rhinorrhea, nasal congestion, pharyngitis, or cough will be discharged to prevent introduction of an intercurrent upper respiratory infection to the population and thereby confounding of the clinical assessment.

On the morning of the second full challenge phase day (challenge phase day 2; day 41 ± 3 after first immunization), all remaining eligible and consenting subjects will receive a dose of viable, egg-grown A/Panama/2007/99 by intranasal drops. The challenge inoculum will be selected so as to achieve illness and infection rates of 50 - 70% in the subjects who received placebo during the immunization phase.

Between challenge phase day 2 (p.m.) and challenge phase day 9 (p.m.), subjects will complete a standard diary card of symptoms of influenza twice each day. During these days, oral temperatures will be measured at least four (4) times daily, and contents of the diary cards will be elicited by a directed interview and recorded, approximately every twelve (12) hours. Once per day, at approximately 24 hour intervals, each subject will have a directed physical examination of the ears, nose, throat, and chest. Once per day, and at approximately 24 hour intervals, each subject will have a nasal wash **and throat swab for detection and / or quantitation of influenza virus using culture, antigen detection or molecular biology techniques.** Tissue counts and weights will be collected throughout challenge as a surrogate of disease severity. Beginning on the morning of challenge phase day 8, each subject will begin five (5) days of twice-a-day treatment with oseltamivir to ensure termination of virus shedding. Amantidine will be available as an alternative treatment should intolerance to oseltamivir be a problem for any subject. Subjects will be re-evaluated in the p.m. hours of challenge phase day 9, and, if illness has resolved, be discharged. Any subject who is either febrile (oral temperature ≥ 37.9º C) or has persistent symptoms of grade 2 or greater will be retained in the facility for an additional 24 hours. Subjects remaining symptomatic will be referred for appropriate medical care. (Illness following inpatient influenza challenge is typically somewhat milder than community- acquired influenza, and short lived. This inpatient period, even without antiviral treatment, extends beyond the typical duration of virus shedding in this model.)

All subjects (challenged and unchallenged) will return between challenge phase days 20 to 30 and be queried regarding adverse events or changes in concomitant medications since discharge; a physical examination will be performed as needed to evaluate complaints and a follow-up ECG will be obtained if any abnormalities were noted at discharge. Serum and nasal wash specimens will be obtained for specific antibody determinations and the subjects discharged from the study.

# 6.0 Investigational Supplies:

## 6.1 Study Drugs:

Proteosome-trivalent influenza vaccine (*PINK LABEL*): a sterile, colorless to yellowish opalescent liquid containing 150 ± 30 μg / mL of hemagglutinin (HA) from each of A/New Caledonia /20/99 (H1N1), A/Panama/2007/99 (H3N2), and B/Shangdong/7/97 viruses in 10 mM Na/K phosphate-buffered isotonic saline, pH 7.4, with 0.01% thimerosal. Non-hemagglutinin viral proteins are also present. The virus components are formulated with outer membrane proteins (OMP) of *Neisseria meningitidis* strain 8047, at an initial ratio of OMPs to HA of 4:1. The overall total protein to hemagglutinin ratio in the final vaccine product is 2.5 – 5:1 (varies slightly with viral strain and non-hemagglutinin protein losses during the complexing process). The vaccine also contains trace amounts of egg proteins. The vaccine is delivered in 3 mL borosilicate glass vials containing **an 0.9** mL fill.

Phosphate-buffered normal saline diluent/placebo (*WHITE LABEL*): a sterile, colorless liquid comprising 10 mM Na/K phosphate-buffered isotonic saline, pH 7.4, with 0.01% thimerosal, delivered in 20 mL borosilicate vials with a 10 mL fill.

## 6.2 Delivery System for Study Drugs:

Valois VP3/140 nasal spray pumps comprising a screw-top vial, an actuator, and a nose piece will be supplied. These devices deliver a metered dose of 140 μL of liquid as an atomized spray with each full depression of the actuator. Delivery of the test article volume by this device has been shown to be ± 5% of nominal volume with each discharge, and median droplet size in the spray is 40 – 50 μm.

## 6.3 Storage Requirements for Study Drugs:

Proteosome-trivalent influenza vaccine should be stored at a controlled temperature of 5 ± 3° C in a secured refrigerator until use. It should NOT be frozen. The vaccine should be used within eight (8) hours of dilution of the stock material and within four (4) hours of removal from refrigeration. While the vaccine contains thimerosal, stock vaccine vials are NOT intended for repeated use over multiple days. Thus, while several doses may be prepared from a single vial of stock vaccine on a given day, residual contents of an opened vial must NOT be used thereafter.

Phosphate-buffered saline should be stored at room temperature away from direct light. Once opened, the contents of a vial may be used for up to eight (8) hours, but residual contents must NOT be used thereafter.

## 6.4 Test Article Dose Preparation:

### 6.4.1 Randomization / Immunization Phase Day 0:

On Study Day 0, the investigational pharmacist (or another unblinded member of site staff who is designated to serve as pharmacist and will have NO involvement whatever with any clinical outcome measurement) will be provided with each eligible, and consenting subject’s initials, subject ID number (SID), and gender. The investigational pharmacist will use the gender-specific randomization list provided by the sponsor to assign the next available randomization code and determine treatment assignment. For each subject randomized, a label bearing the subject’s SID, initials, and randomization code (a two-digit code that does not itself, in the absence of the unblinded treatment list, reveal treatment assignment) will be prepared and affixed to a metered dose pump. In addition, the SID will be transcribed onto the Treatment Group Enrollment Log. The investigational pharmacist will then prepare the appropriate test article for each subject.

For Immunization Phase Day 14 doses, the investigational pharmacist will consult the Treatment Group Enrollment Log to confirm the appropriate treatment group for each subject, and hence the appropriate test article, and then prepare a label bearing the subject’s SID, initials, and randomization code and affix it to a metered dose pump.

### 6.4.2 Preparation of Individual Test Article Doses:

A test article dose will be prepared for each subject to be dosed by placing a total of 0.9 mL of appropriately diluted active vaccine or placebo/diluent, as dictated by the treatment group assignment, into the vial of the previously labeled metered dose pump, after which the actuator and nose piece will be assembled to the pump. Since the target concentrations of each of the three HA species in the vaccine are approximately equal, dosing and dilution will be based on the mean HA concentration in the trivalent (i.e., the average of the concentrations of the three different HAs). Detailed instructions and dose preparation worksheets are provided as Appendix A. After assembly of the pump, the pharmacist will hold the metered dose pump upright, gently swirl once again, remove the nosepiece and then the safety collar, and firmly and fully depress the actuator three (3) times to prime the pump. The collar and then the nosepiece cover will be replaced after priming, and the labeled, filled, and primed pump will be delivered to the clinic.

After test article preparation is completed for a given day, any opened stock vaccine and/or saline placebo vials will be restoppered and retained for retrieval by an ID Biomedical representative.

## 6.5 Test Article Dose Administration:

All study drugs are to be administered under the supervision of the Investigator or a qualified physician subinvestigator / study doctor designated to ID Biomedical in writing prior to the trial and trained in both the protocol and contents of the Investigators’ Brochure. Under no circumstances will the Investigator allow study drugs to be used other than as directed by this protocol.

Test articles will be administered as follows:

• The nosepiece cover will be removed, and then the safety collar.

- The nose piece of the metered dose pump will be placed in the subject’s right nostril while the subject occludes the left.

• As the subject gently inhales through the nose, the actuator will be firmly and fully depressed.

• The nose piece of the metered dose pump will be placed in the subject’s left nostril while the subject occludes the right.

- As the subject gently inhales through the nose, the actuator will be firmly and fully depressed.

• The used pump will be retained for drug accountability and eventual return to the sponsor.

## 6.6 Accountability for Study Drugs:

The Investigator or a designee is responsible for maintaining complete drug inventory records accounting for receipt, storage, dispensation, and final disposition using forms supplied by (or local equivalents approved by) ID Biomedical. These records will be reviewed by ID Biomedical representatives. At the conclusion of the trial all vials of study drugs, used and unused, and all metered dose pumps will be returned to the sponsor.

6.7 Challenge Virus:

The challenge virus is A/Panama/2007/99 (H3N2) lot I B 44/3 produced in eggs by Berna Biotech Ltd. This material is sterile and mycoplasma-free. The titer of this virus as provided by the manufacturer is 10^8.46^ egg ID_50_ / mL, and it is provided in cryovials. Prior to challenge, the virus will be diluted to the predetermined desired challenge inoculum in sterile isotonic phosphate-buffered saline.

6.8 Storage of Challenge Virus:

Challenge virus should be stored at ≤ - 70º C in a secured freezer until use. Vials should be thawed just prior to dilution. Once thawed and entered, vials of stock virus should NOT be reused for human studies.

6.9 Disposal of Challenge Virus:

Used challenge virus stock vials and dilutions will be disposed of according to Retroscreen Ltd’s SOPs for the destruction of biohazardous waste.

# 7.0 Procedures and Methods:

## 7.1 Procedures:

The following procedures will be done at the indicated times (see also Appendix B: IDB-13005 Time and Events Tables B.1 and B.2).

### 7.1.1 First Screening Visit (days - 150 to - 7):

• Written, witnessed informed consent for screening will be obtained.

- A brief medical history will be obtained to ensure that the subject meets historical criteria for enrollment, and approximately 10 mL of blood will be drawn for A/Panama/2007/99 serum HAI antibody screening determination (see section 7.2).

### 7.1.2 Second Screening Visit (days -90 to - 5):

- This visit will occur within the above interval, but not prior to completion of A/Panama/2007/99 HAI determination on the “first screening” specimen.
- Subjects’ eligibility for enrollment based on history and HAI data will be reviewed.
- A detailed explanation of the clinical trial will be provided to subjects remaining eligible, and **written informed consent** to trial participation will be elicited.
- Vital signs and a complete baseline physical examination will be performed and recorded.
- An ECG will be performed.
- Approximately 25 mL of blood will be drawn and a urine specimen obtained for baseline clinical laboratory tests, **drug screening,** and HIV serologies (see section 7.2).
- A nasal wash specimen for specific influenza antibody assay will be obtained (section 7.2).

### 7.1.3 Immunization Phase Day 0, First Treatment:

• Subjects’ eligibility for enrollment based on ECG and clinical laboratory data from the prior screening visit will be reviewed.

• An interval medical history will be performed to ensure that each subject continues to meet all inclusion and no exclusion criteria.

- A urine specimen will be collected from each female subject and tested in the clinic for evidence of pregnancy. No test article will be administered until a negative result is obtained and documented.
- A directed examination of the ears, cervical lymph nodes, and nasopharynx will be done, the results of which will be recorded using the worksheet provided as Appendix C.
- Each subject will be interviewed regarding the presence of baseline upper respiratory complaints and / or symptoms of systemic illness prior to dosing, using a “Memory Aid” format consistent with that presented in Appendix D.
- Volunteers with any grade 2 physical findings or grade 2 symptoms among their baseline Memory Aid observations will not be given a test article dose. Such volunteers may return at a later time point and be considered for enrollment if findings/symptoms resolve.
- Approximately 30 mL of venous blood will be obtained for baseline *in vitro* cytokine stimulation studies and HAI antibody titers.
- Test article will be administered intranasally as described in section 6.5. (*Subjects who receive any amount of test article will be considered enrolled and followed for safety and immunogenicity through the Challenge Phase Day 20 -30 visit as specified below and in section 8.0 (regardless of whether the subject receives further test article or is challenged). No additional follow-up will be required of subjects who are screened but do not receive any test article*.)

• Subjects will be observed for thirty (30) minutes after dosing. At the end of this interval, each subject will have a repeat oral temperature measured and recorded and complete a questionnaire eliciting immediate post-immunization symptoms (present at the time of observation or in the preceding 30 minutes). Each will be graded as: none, mild (just noticeable), moderate (unpleasant/uncomfortable, but not incapacitating), or severe (preventing resumption of normal activities). The questionnaire is provided as Appendix E.

- Each subject will be provided with a Memory Aid (Appendix D) and a digital thermometer and instructed to take his/her temperature and complete a Memory Aid page each evening from Day 0 to Day 6. Subjects will be instructed to notify the clinic (Retroscreen) should they experience grade 2 or greater symptoms, and to return as instructed for physician evaluation.
- Subjects will be instructed to return to the clinic (Retroscreen) on Day 7, with their Memory Aid

### 7.1.4 Immunization Phase Days 0 – 6 (0 to 6 days after the First Treatment)

- Subjects will maintain their Memory Aids and measure evening temperatures daily.
- Subjects will report any grade 2 or greater symptoms, or fever (oral temperature ≥ 37.9º C) to study site personnel, who will arrange for a physician evaluation.
- Physician evaluations will be performed as needed, and recorded in a uniform manner utilizing the worksheet provided as Appendix C.

### 7.1.5 Immunization Phase Day 7 (± 1 day, Follow-up Visit One):

• Vital signs will be taken and recorded.

• Each subject’s Memory Aid will be reviewed, collected, and the data abstracted and recorded. Subjects who have lost their Memory Aids will be provided with new documents, which will be completed from memory with the assistance of the interviewer. Such data will be clearly annotated as RETROSPECTIVE.

• Subjects will be queried regarding the occurrence of adverse events not specified in the Memory Aid, and any new concomitant medications taken, and the results recorded.

• A brief, directed examination of the ears, cervical lymph nodes, and naso-pharynx will be performed to detect objective signs of vaccine-induced reactogenicity. The examiner will complete the brief exam worksheet provided as Appendix C.

• Subjects will be given an appointment to return to the clinic on Immunization Phase Day 14 ± 2.

### 7.1.6 Immunization Phase Day 14 (± 2 days; Second Treatment):

- Vital signs will be taken and recorded
- An interval medical history will be performed to ensure that each subject continues to meet all inclusion and no exclusion criteria.
- A urine specimen will be collected from each female subject and tested in the clinic for evidence of pregnancy. No test article will be administered until a negative result is obtained and documented.
- A directed examination of the ears, cervical lymph nodes, and nasopharynx will be performed, the results of which will be recorded using the worksheet provided as Appendix C.
- Each subject will be interviewed regarding the presence of baseline upper respiratory complaints and / or symptoms of systemic illness prior to dosing, using a “Memory Aid” format consistent with that presented in Appendix D.
- Volunteers with any grade 2 physical findings or grade 2 symptoms among their baseline Memory Aid observations will not be given a test article dose.
- These subjects may return to the clinic up to Immunization Phase Day 21 and, if symptoms or findings have resolved, receive the second test article dose. Up to Immunization Phase Day 16, this will not be recorded as a protocol deviation; from Immunization Phase Days 17 to 21 a deviation will be recorded but the dose will be given. Such subjects will complete all protocol-mandated follow-up per schedule and *will* be eligible for challenge.
- If grade 2 symptoms or findings persist beyond Immunization Phase Day 21, the second dose will be withheld. Such subjects will not be eligible for challenge, but will complete all other protocol-mandated follow-up.

• Test article will be administered intranasally as described in section 6.5.

• Subjects will be observed for thirty (30) minutes after dosing. At the end of this interval, each subject will have an oral temperature measured and recorded and complete a questionnaire eliciting immediate post-immunization symptoms (present at the time of observation or in the preceding 30 minutes). Each will be graded as: none, mild (just noticeable), moderate (unpleasant/uncomfortable, but not incapacitating), or severe (preventing resumption of normal activities). The questionnaire is provided as Appendix E.

• Each subject will be provided with a Memory Aid (Appendix D) and a new digital thermometer (if needed) and instructed to take his/her temperature and complete a Memory Aid page each evening from Immunization Phase Days 14 to 20. Subjects will be instructed to notify the clinic (Retroscreen) should they experience grade 2 or greater symptoms, and to return as instructed for physician evaluation.

• Subjects will be instructed to return on Immunization Phase Day 21, with their Memory Aid.

### 7.1.7 Immunization Phase Days 14 to 20 (0 – 6 days after the Second Treatment)

- Subjects will maintain their Memory Aids and measure evening temperatures daily.
- Subjects will report any grade 2 or greater symptoms, or fever (oral temperature ≥ 37.9º C) to study site personnel, who will arrange for a physician evaluation.
- Physician evaluations will be performed as needed, and recorded in a uniform manner utilizing the worksheet provided as Appendix C.

### 7.1.8 Immunization Phase Day 21 (± 2 days; Follow-up Visit Two):

• Vital signs will be taken and recorded.

• Each subject’s Memory Aid will be reviewed, collected, and the data abstracted and recorded. Subjects who have lost their Memory Aids will be provided with new documents, which will be completed from memory with the assistance of the interviewer. Such data will be clearly annotated as RETROSPECTIVE.

• Subjects will be queried regarding the occurrence of adverse events not specified in the Memory Aid, and any new concomitant medications taken, and the results recorded.

• A brief, directed examination of the ears, cervical lymph nodes, and naso-pharynx will be performed to detect objective signs of vaccine-induced reactogenicity. The examiner will complete the brief exam worksheet provided as Appendix C.

• Subjects will be given an appointment to return to the clinic on Immunization Phase Day 28 ± 3.

### 7.1.9 Immunization Phase Day 28 (± 3 days; Follow-up Visit Three):

- Subjects will be queried regarding the occurrence of adverse events and any new concomitant medications taken, and the results recorded.
- A physical examination will be performed *if required* to evaluate any complaints, and new abnormalities will be captured as adverse events.

• A nasal wash specimen for specific antibody assay will be obtained from each subject (see section 7.2).

• Approximately 40 mL of blood will be drawn from each subject for HAI antibodies, *in vitro* cytokine stimulation studies, and clinical laboratory tests (see section 7.2).

- Each subject will provide a urine specimen for determination of blood, glucose, and protein.

### 7.1.10 Challenge Phase Day 0 (39 ± 3 days after First Treatment; Admission to Quarantine):

• All subjects will be admitted to the quarantine unit.

- An interval medical history will be performed to ensure that each subject continues to meet all inclusion and no exclusion criteria (excepting influenza vaccine and serum HAI criteria, which will have become irrelevant due to the immunization component of this protocol).
- Subjects will be queried regarding the occurrence of adverse events and changes in concomitant medications, and the results recorded.
- Subjects will complete an Influenza Symptom Diary Card (see Appendix F), and will be interviewed by a physician re: potential symptoms of influenza or upper respiratory infection.
- A complete physical examination, including vital signs with oral temperature, will be performed and recorded.
- An ECG will be performed.

• A nasal wash specimen for specific antibody assay will be obtained from each subject (see section 7.2).

• Approximately 15 mL of blood will be drawn from each subject for specific HAI serologies (see section 7.2).

7.1.11 Challenge Phase Day 1:

• Subjects will have oral temperature measured and recorded four times a day.

- Subjects will complete an Influenza Symptom Diary Card (see Appendix F), and will be interviewed by a physician, approximately every 12 hours.
- Subjects will be queried regarding occurrence of AEs not specified in the Influenza Symptom Diary Card, and these will be recorded.
- A directed physical examination using the Challenge Physical Examination Worksheet (see Appendix G) will be performed and recorded in the evening.
- Subjects will be instructed in, and begin, collection of used tissues for 24 hour-counts and weights.
- Any subject with grade 2 findings or complaints and/or an oral temperature ≥ 37.9 º C (confirmed by repetition at an interval of not less than 20 minutes) will be immediately segregated from the remaining subjects and discharged if any suspicion of an **intercurrent** respiratory infection exists. The examining physician will provide medical referral for outpatient care if deemed necessary. The cause of such discharge will be recorded as an adverse event. The subject will be instructed to return for the day 60 outpatient visit.

### 7.1.12 Challenge Phase Day 2, Morning (Day of Challenge):

- Subjects will complete a morning Influenza Symptom Diary Card (see Appendix F).
- Subjects will be queried regarding occurrence of AEs not specified in the Influenza Symptom Diary Card, and these will be recorded.
- A urine specimen will be collected from each female subject and tested in the clinic for evidence of pregnancy. No challenge virus will be administered until a negative result is obtained and documented.
- Any subject with grade 2 complaints and/or an oral temperature ≥ 37.9 º C (confirmed by repetition at an interval of not less than 20 minutes and not more than 60 minutes) will be immediately segregated from the remaining subjects and discharged if any suspicion of an intercurrent respiratory infection exists. The examining physician will provide medical referral for outpatient care if deemed necessary. The cause of such discharge will be recorded as an adverse event. The subject will be instructed to return for the day 60 outpatient visit.
- Each subject remaining eligible will provide a throat swab for detection and / or quantitation of influenza virus **using culture, antigen detection and / or molecular biology techniques** (see section 7.2).

• Each subject remaining eligible will be challenged with A/Panama/2007/99 delivered nasally by dropper.

7.1.13 Challenge Phase Day 2 Afternoon to Challenge Phase Day 9 Morning.:

- Subjects will complete an Influenza Symptom Diary Card (see Appendix F), and will be interviewed by a **member of the medical team**, approximately every 12 hours.
- Subjects will have an oral temperature measured and recorded four times a day.
- Subjects will be queried regarding occurrence of AEs not specified in the Influenza Symptom Diary Card, and these will be recorded.
- A directed physical examination using the Challenge Physical Examination Worksheet (see Appendix G) will be performed and recorded.
- Subjects will continue 24-hour tissue collections for counts and weights.
- Every morning (beginning on Challenge Phase Day 3), *after physical examinations are completed*, a nasal wash and a throat swab will be obtained **from each subject for detection and / or quantitation of influenza virus using culture, antigen detection and/or molecular biology techniques** (see section 7.2).
- Symptomatic treatment with paracetamol (1000 mg orally no more than q.i.d.) will not be offered, but may be given upon subject request *if and only if*:
  - The subject has an oral temperature ≥ 38.5º C (confirmed by a second measurement separated by not less than 20 minutes or more than 60 minutes, or
  - The subject has a confirmed oral temperature of ≥ 37.9º C, and at least one concurrent symptom of grade 2 or greater severity, or
  - The subject has two or more concurrent symptoms of grade 2 or greater severity.
- **Specific treatment with oseltamivir 75 mg orally administered twice daily may be begun before Challenge Phase Day 8 *if and only if*:**
  - **The subject has a confirmed oral temperature ≥ 39.0º C, or**
  - **The subject has a confirmed oral temperature of ≥ 38.5º C, and at least two (2) concurrent symptoms of grade 2 or greater severity, or**
  - **The subject has three (3) or more concurrent symptoms of grade 2 or greater severity or two (2) symptoms of grade 3 severity**
- On the MORNING of Challenge Phase Day 8, all subjects will initiate treatment with oseltamivir (75 mg orally), to be administered twice daily at approximately 12 hour intervals for five (5) days. Subjects who are intolerant of oseltamivir – most commonly manifesting with nausea or other gastrointestinal symptoms which are atypical for influenza – may be treated with amantidine hydrochloride 200 mg orally once a day for three days or until influenza symptoms resolve.
- On either Challenge Phase Day 8 or 9, a complete physical examination, including vital **signs, and** an ECG will be performed and the results recorded.

7.1.14 Challenge Phase Day 9 p.m.:

- Oseltamivir or amantidine treatment will continue as per 7.1.13.
- Subjects will complete an Influenza Symptom Diary Card (see Appendix F), and will be interviewed by a physician.
- Subjects will be queried regarding occurrence of AEs not specified in the Influenza Symptom Diary Card, and these will be recorded.
- Subjects who are afebrile (oral temperature < 37.9º C) and free of influenza-like illness symptoms of grade 2 severity, and have no ominous findings on their Challenge Phase Day 8 or 9 physical examination may be discharged with instructions to return to the clinic for the Challenge Phase Day 20 – 30 outpatient visit. Subjects will be supplied with sufficient oseltamivir or **amantidine** to complete their five-day regimen, and reminded to do so. Any subject who is either febrile (temperature > 37.9º C) or has persistent symptoms of grade 2 or greater (see diary card) will be retained in the facility for 24 hours additional observation and drug treatment unless the Investigator records a clear and compelling medical rationale for allowing discharge. Subjects who continue significantly symptomatic will be referred for appropriate medical care as needed.

7.1.15 Challenge Phase Day 20 – 30 (56 – 72 days after First Treatment; Final Follow-up Visit)

- Subjects will be queried regarding the occurrence of adverse events and any new concomitant medications taken, and the results recorded.
- A complete physical examination, including vital signs, will be performed and recorded.
- An ECG will be repeated if significant new abnormalities were noted prior to discharge from the quarantine facility.
- A nasal wash specimen for specific antibody assay will be obtained from each subject (see section 7.2).
- Subjects will provide a 15 mL sample of blood for HAI antibody testing (see section 7.2).
- This visit marks completion of protocol IDB-13005.

## 7.2 Laboratory Tests:

### 7.2.1 Hematology:

The following will be done at the Second Screening Visit (days -90 to -5) and on Immunization Phase Day 28 (± 3 days; Follow-up Visit Three), and if clinically indicated to evaluate illness during challenge.

• Hematocrit

• Hemoglobin

• White blood cell count and differential

• Platelet count

### 7.2.2 Clinical Chemistry:

The following will be done at the Second Screening Visit (days -90 to -5) and on Immunization Phase Day 28 (± 3 days; Follow-up Visit Three), and if clinically indicated to evaluate illness during challenge.

• ALT and AST

• Blood urea nitrogen and Creatinine

### 7.2.3 Clinical Laboratory Serologies:

The following serum determinations will be done at the Second Screening Visit:

• Anti-HIV antibodies

### Urinalysis:

The following analytes will be recorded at the Second Screening Visit (days -90 to -5) and on Immunization Phase Day 28 (± 3 days; Follow-up Visit Three), and if clinically indicated to evaluate illness during challenge.

- Glucose, Protein, and Hemoglobin
- **Drugs of abuse screening (second screening visit only)**

Any sample with any positive glucose, or any protein or hemoglobin > trace, will trigger performance of a complete urinalysis, including microscopic examination.

### 7.2.5 Pregnancy Testing:

Urine β-HCG determination will be performed on a freshly-obtained urine specimen on the day of each test article dose and on the day of challenge dosing on each female subject not having a verifiable history of hysterectomy. A negative result must be obtained and recorded before any test article or challenge inoculum is administered.

### 7.2.6 Specific Immune Response Assays :

• Serum will be obtained for influenza-specific special serologies (serum hemagglutination-inhibition titers) at the First Screening Visit (A/Panama/2007/99 only), at the Immunization Phase Day 0 visit (all three strains tested for this and subsequent samples), and on Immunization Phase Day**,** 28 (± 3), Challenge Phase Day 0, and Challenge Phase Day 20 - 30. Should hemagglutination-inhibition prove unworkable for the influenza B virus component, single radial hemolysis assay may be utilized.

- Nasal washes for influenza-specific secretory IgA by ELISA will be obtained at the Second Screening Visit (all three strains tested for this and subsequent samples), and on Immunization Phase Day**,** 28 (± 3), Challenge Phase Day 0, and Challenge Phase Day 20 - 30.
- Whole blood will be obtained for *in vitro* cytokine stimulation studies on Immunization Phase Day 0 and Immunization Phase Day 28 (± 3).

### 7.2.7 Influenza Virus Detection:

- **On Challenge Phase Day 2 PRIOR to challenge, a throat swab will be obtained for detection and or quantitation of influenza virus using culture, antigen detection and / or molecular biology techniques.**
- **From Challenge Phase Day 3 to Challenge Phase Day 9, a nasal wash and throat swab will be obtained daily from each subject for detection and / or quantitation of influenza virus using culture, antigen detection and / or molecular biology techniques.**

## 7.3 Other Measurements:

### 7.3.1 History and Physical Examination:

A complete physical examination and medical history/review of systems will be performed and recorded before the first test article dose. Vital signs will be included in all complete physical examinations. Significant abnormalities in the physical examination will be recorded on the case report forms. The medical history should record significant problems active at the time of screening or within the last year. Problems which have been inactive within the last year, but which might alter the subject’s current or future management, should also be noted (e.g., known mitral valve prolapse, history of seizure disorder, etc.) The physical examination will be repeated as needed for evaluation of complaints during the trial, and a complete physical examination will be repeated prior to challenge. A third complete examination will be performed on Challenge Phase Day 8 or 9 prior to discharge from the isolation facility. Additional repeat examinations will be performed at the Challenge Phase Day 20 -30 visit only as necessary to evaluate any new complaints. Any new and clinically-significant abnormalities will be recorded as adverse events.

### 7.3.2 Brief, Directed Physical Examination after Treatments:

A brief, directed physical examination will be performed on Immunization Phase Days 0, 7, 14 and 21 to ensure eligibility for treatment (0 and 14) and to evaluate the presence of objective findings of vaccine reactogenicity (7 and 21). This examination will encompass the ears, nose, throat, and cervical lymph nodes, and will be recorded using a standardized worksheet (Appendix C). It is emphasized to site staff that continuity of observer for any given subject is important for these examinations and should be maintained if at all practicable.

### 7.3.3 Temperature Measurements During the Challenge Phase:

From Challenge Phase Day 1 to Day 9, oral temperature will be recorded at least four (4) times daily. Because these readings are critical to definitions of illness and to symptomatic and specific therapy criteria, temperatures of ≥ 37.6ºC must be confirmed by a repeat measurement not less than 20 minutes and not more than 60 minutes after the first reading. Subjects will be instructed not to eat or drink during the interval between the two measurements.

### 7.3.4 Directed Physical Examination During the Challenge Phase:

A directed physical examination, including pulse and blood pressure, will be performed and recorded on every day from Challenge Phase Day 1 to Day 9 in accordance with the Challenge Physical Examination Worksheet (see Appendix G). The physician investigator may, at his/her judgement, perform any additional vital signs or physical examination necessary to evaluate or manage clinical illness. It is emphasized to site staff that continuity of observer is again desirable and should be maintained if at all practicable.

### 7.3.5 Concomitant Medications:

Subjects should be instructed not to introduce new medications without consulting or notifying the investigator or his designee. Subjects will be questioned regarding new medications at each visit through Challenge Phase Day 20 – 30 (Final Follow-up Visit) and any such medications recorded on the case report form.

## 7.4 Early Discontinuation:

Every reasonable effort should be made to ensure that each subject complies with the protocol and completes all study visits. However, a subject may withdraw or be withdrawn from participation if:

• The subject withdraws consent,

- The investigator recommends discontinuation in the interest of the subject’s safety or because of significant and irremediable protocol non-compliance,

• ID Biomedical, the ethics committee, or the MCA terminates the clinical trial.

Subjects must be stringently counseled that early withdrawal from the challenge isolation portion of the trial will be strongly discouraged, as it poses a risk both to the subject and to his/her contacts. Any subject insisting on early withdrawal during challenge isolation will be started immediately on a five (5) day course of oseltamivir (or amantidine), and will be encouraged to remain in the isolation unit as long as possible.

A complete Challenge Phase Day 20 – 30 (Final Follow-up Visit) evaluation and Study Termination case report form should be completed on any subject prematurely withdrawn from the clinical trial. No subject prematurely withdrawing/withdrawn will be replaced.

# 8.0 Immediate Complaints, Vaccine Reactogenicity, Findings of Influenza and Adverse Events:

## Immediate Complaints:

A selection of immediate complaints reasonably anticipated to occur as a result of receipt of the test articles is provided as a questionnaire (Appendix E) to be completed approximately 30 minutes after each test article dose and transcribed to the case report forms. The data will be reported as immediate complaints and presumed to be related to the test article. *These findings should NOT be additionally recorded as adverse events (see below) unless they fulfill the criteria set forth in section 8.4.1.*

## 8.2 Vaccine Reactogenicity:

A selection of subjective complaints reasonably anticipated to occur as a result of receipt of the test articles are provided in the Memory Aid (see sample in Appendix D), and a selection of potential physical findings also reasonably anticipated to occur as a result of receipt of the test articles are provided on the Brief Examination Worksheet (Appendix C) to be used on Immunization Phase Days 0, 7, 14, 21 and at any time during the seven days after each dose that a subject has grade 2 or greater severity complaints. Findings from these two sources will be recorded on the case report forms, reported as vaccine reactogenicity, and presumed to be related to the test article(s). *These findings should NOT be additionally recorded as adverse events (see below) unless they fulfill the criteria set forth in section 8.4.1.*

Definitions used to grade reactogenicity events are included as an integral part of each Brief Examination Worksheet (Appendix C) and Memory Aid page (Appendix D).

8.3 Symptoms and Findings of Influenza:

During the challenge period (Challenge Phase Days 1 – 9), symptoms and signs of influenza will be collected using the Influenza Symptom Diary Card (see Appendix F) and the Challenge Physical Examination Worksheet (see Appendix G) and their integral definitions. During this interval, findings captured by these two tools will be presumed to represent influenza consequent to challenge, and *will not be additionally captured as adverse events (see below) unless they fulfill the criteria set forth in section 8.4.1.*

## 8.4 Adverse Events:

An adverse event (AE) is any unfavorable, harmful, or pathologic change in a research subject as indicated by physical signs, symptoms and/or clinically significant laboratory abnormalities that occurs in association with the use of a product (trial-related), whether or not considered to be product-related. This includes intercurrent illnesses, injuries, worsening of pre-existing conditions, and events occurring as a result of product abuse or overdose. Stable pre-existing conditions and/or elective procedures to address them are not adverse events. Clinical laboratory findings are considered to be adverse events if regarded as clinically significant by the investigator, or if these cause (or should have caused) a change in the investigational drug regimen, a further diagnostic evaluation, or institution of any therapy.

All events fulfilling any part of the AE definition must be recorded on the adverse event case report form (with the exception of pre-defined immediate complaints or symptoms/signs of vaccine reactogenicity in the first seven [7] days following immunizations, and pre-defined symptoms and signs of influenza during the challenge (Challenge Phase Days 2 – 9); see sections 8.1, 8.2, 8.3, and 8.4.1).

Treatments and procedures are not adverse events; rather, the illness which precipitates them should be recorded. For example, “cholecystectomy,” is not an AE, but the diagnosis of “cholecystitis” or “gall stones” leading to the surgical (or medical) treatment is an AE. Where possible, specific diagnoses are preferred in reporting AEs. Individual complaints or findings may be reported as AEs, but when multiple complaints or findings occur together and can be logically and defensibly assembled into a single clinical syndrome or diagnosis, the latter is preferred. For example, an isolated finding of elevated AST and ALT would be recorded as “elevated transaminases.” If accompanied by fever, anorexia, nausea, and jaundice, however; a report of “hepatitis” would be preferable to a list of individual findings. If the new presence of HBsAg is also found, a more specific diagnosis of “hepatitis B” would be recorded.

### 8.4.1 Double Reporting of Reactogenicity Findings/Complaints and Findings of Influenza as Adverse Events:

As per sections 8.1, and 8.2 above, findings and/or complaints specifically captured by the Immediate Complaints Questionnaire (Appendix E), the Brief Examination Worksheet (Appendix C), or the Memory Aid (Appendix D) should NOT, in general, be additionally recorded as AEs if they occur within seven (7) days of a test article dose.

The rationale for not in general doubly-reporting reactogenicity events as AEs is as follows. The specified range of reactogenicity complaints and findings in Appendices C, D, and E are those reasonably expected (theoretically and by experience) to be associated with a nasal vaccine. They are collected, *presumed to be drug-related*, analyzed, and reported to investigators and regulatory authorities in detail as expected vaccine reactions. Separation of these numerically very frequent, but minor and anticipated, vaccine reactions from other classes of adverse events prevents the obscuration of other potentially important, but less common, types of events in the statistical analysis of AEs.

There are *three exceptions* to the rule regarding double reporting:

1. Any of these findings or complaints which fulfills the definition of “serious adverse event” (see section 8.5) should be recorded as an AE (and reported as per section 8.5),
2. Any of these findings or complaints that persists beyond seven (7) days after a test article dose should be recorded as an AE,
3. Any of these findings or complaints which the investigator *unequivocally* categorizes, based on strong clinical evidence, as unrelated to the test article should be recorded as an AE. In this case the investigator *must* assign a causality of “probably not related” (not “unknown”) and record a clear rationale in source documents.

If a reactogenicity finding or complaint is also recorded as an AE, site personnel should take care to record a verbatim AE identical to the wording of the reactogenicity finding/complaint, and also an AE start date identical to the onset

date implied by the reactogenicity case report forms. The sponsor will report these events as both reactogenicity and AE findings, but will be able to link the two reports in the database for discussion and analysis purposes.

As per section 8.3 above, the symptoms and physical findings of influenza captured by the Influenza Symptom Diary Card (Appendix F) and the Challenge Physical Examination Worksheet (Appendix G) will also not in general be additionally recorded as AEs. The sole exception to this rule will be that symptoms or physical findings of influenza which fulfill, singly or collectively, the definition of “serious” as per section 8.5 will be recorded as serious AEs and be reported in accordance with section 8.5.

## 8.5 Serious Adverse Events:

A serious adverse event (SAE) is any adverse event that results in any of the following outcomes: a) death, b) an immediate threat to life, c) inpatient hospitalization or prolongation of an existing hospitalization, d) persistent or significant disability / incapacity, or e) a congenital anomaly / birth defect. Important medical events that do not result in one of these outcomes, but, based on appropriate medical judgement, are deemed to jeopardize the subject or require medical or surgical intervention to avert one of the listed outcomes, may also be considered SAEs. Serious adverse events must be reported to ID Biomedical within 24 hours of the investigator’s learning of the event. The initial report may be verbal, or may utilize the SAE worksheet provided by ID Biomedical and be transmitted by facsimile. Reports may be made to:

Dr. Louis F. Fries, at:

• 01−410-455-5610 (office)

• 01−410-455-5606 (fax)

• 01−443-253-5298 (mobile)

• 01−410-964-1326 (home)

Or alternatively: Dr. Peter Vink, a back-up contact, at:

• 01−410-455-5613 (office)

• 01−443- 226-2396 (mobile)

• 01−410-455-5606 (fax)

In the case of a verbal report, a written report using the ID Biomedical, SAE worksheet (which may subsequently be updated if additional data become available) should be provided by facsimile within 72 hours. All SAEs must also be reported as soon as possible to the ethics committee reviewing and approving the clinical trial.

# 9.0 Analysis Plan:

This is a phase II, four-group, placebo-controlled, randomized clinical trial. Approximately 28 subjects will be treated in each group. Volunteers will either receive two 15 µg treatments separated by two weeks, one 30 µg and one placebo treatment separated by two weeks, two 30 µg treatments separated by two weeks, or two placebo treatments separated by two weeks. In the immediate post-treatment period, outcomes are pre-specified local and systemic reactions, general adverse event rates, and immune responses assessed by antibody measurements.

Forty-one (± 3) days after the first treatment, subjects who remain eligible are challenged and then followed for an additional week regarding evidence of illness (by several alternative definitions), and also concerning virologic outcomes.

All analyses will be done with SAS, version 8.2 or later, primarily PROC UNIVARIATE, FREQ, LOGISTIC (for logistic regression with challenge data), and GENMOD (SAS PROC GENMOD implements the repeated measures regression model of Zeger and Liang [48].) Chi-square tests in PROC FREQ are exact. In this phase II trial which will not be used to provide definitive evidence of efficacy or safety, p-values of 0.05 or less for two-sided statistical tests will be considered significant and multiple comparison corrections will not be used (although the rather large number of significance tests – at least as regards safety -makes it likely that the type I error rate will be somewhat elevated.

Simple methods will be used to impute missing data. For binary outcomes, such as the presence/absence of an adverse event, alternative imputations will assume the event did, and did not, occur and the results of these contrary assumptions will be compared. For continuous outcomes, such as antibody titers, last-value-carried-forward will be used except that two consecutive missing values will not be imputed.

## 9.1 Immunogenicity:

Immunogenicity measures will include serum hemagglutination-inhibiting (HAI) antibody titers specific for the three virus strains included in the vaccine, and levels of secretory IgA (sIgA) specific for these viruses measured in nasal wash fluids. Antibody levels in nasal wash specimens will be assayed by kinetic ELISA and normalized on total content of sIgA. For the HAI titers, analyses will concern geometric mean titers (GMT), proportions of subjects with titer ≥ 40, and proportions with ≥ four-fold increase over baseline. For specific sIgA levels, analyses will concern geometric means and fold-rise from baseline.

HAI GMTs and geometric mean nasal specific sIgA levels will be accompanied by 95%, Student’s t-based confidence intervals. Between active-to-placebo and active-to-active cross-over groups, titers/antibody levels will be compared on day 28 by t-tests on log_10_ (titer). Within the groups, paired tests will be used (Student’s t and Wilcoxon signed rank). Fold-rises may be examined with and without covariate-adjustment for previous titers. Without such adjustment, there are two binary fold-rise outcomes, and these are compared within and between groups by chi-square and stratified chi-square tests. (In the latter case, 2 x 2 tables of treatment by outcome are stratified by previous titers.) With covariate adjustment, the fold-rise outcome is the log_10_-ratio of early and later titers. Adjustment after the first treatment is for baseline titer and after the second treatment is for both baseline titer and titer after the first treatment. This facilitates contemporaneous between-group comparisons of placebo and active after both periods 1 and 2.

9.2 Safety:

### 9.2.1 Power and Detectable Effect Size:

In regard to the immediate post-treatment component of the study design, we address the following two questions: What must be the chance of a given reactogenicity event/finding or adverse event in a single subject for there to be a high likelihood (probability 0.80) of a few occurrences in 28 (any one treatment group) and 84 (all active treatment) subjects? Secondly, comparing three cross-over groups in the same period, what must be the relative risk of a reactogenicity event/finding or adverse event for the groups to differ significantly in risk? Exact calculations are based on the binomial distribution.

| Chance of an event in a single subject so that there is an 80% chance of at least the given number of events in 28 or 84 subjects | | | |
| --- | --- | --- | --- |
|  | ≥ 1 | ≥ 2 | ≥ 3 |
| n = 28 | 0.056 | 0.103 | 0.146 |
| n = 84 | 0.019 | 0.035 | 0.050 |

Thus, events that occur with probability of about 6% and 2% in a single subject imply an 80% chance of seeing at least one event in, respectively, any given treatment group and all actively-treatment subjects. This phase II study is not intended to be powered to detect rare events.

Turning to comparison of any two cross-over groups, 28 subjects in each, in the same period, we compute the relative risk (RR) that gives 80% power, type I error 5%, to detect RR ≠ 1.

| Chance of an adverse event in a placebo vaccinee so that a given risk-ratio yields a significant effect (80% power, 5% type I error) | | | |
| --- | --- | --- | --- |
| relative risk | 5-fold | 3-fold | 2-fold |
| placebo rate | 0.074 | 0.178 | 0.381 |

Thus, for a five-fold relative risk of an adverse event or reactogenicity event, active-to-placebo, to be detectable (with good power), the chances of an adverse event in the placebo group must be 0.074. With a lower adverse event rate among placebo subjects, there would not be good power to detect even a five-fold increase in risk However, for the most frequent reactogenicity events observed with intranasal proteosome-influenza vaccine, mild rhinorrhea and nasal congestion, prior experience indicates that placebo rates will range from 0.2 – 0.3. Thus, there will be adequate power to detect between two- and three-fold increases in risk in active product recipients.

### 9.2.2 Immediate Complaints, Vaccine Reactogenicity Complaints, and Standardized Ear, Nose, and Throat Exams:

Immediate complaint data derived from 30 minute post-dose questionnaires will be tabulated for each treatment group by categories of complaints and by severity. Vaccine reactogenicity data derived from memory aids and physician examinations will be similarly tabulated by treatment group, categories of complaints or findings, severity, and duration.

Comparison of graded severities for local and systemic reactogenicity: Comparing one group with another during a period (the cross-over design has two periods), these are two-sample, cross-sectional comparisons of binary, ordinal, or continuous data. Comparing a group with itself between periods, these are paired, repeated measures data. Two-sample, cross-sectional comparisons are based on Fisher’s exact and Cochran-Mantel-Haenszel tests for binary and ordinal outcomes, respectively. Student’s-t and Wilcoxon tests are used for continuous outcomes. These methods permit statistical tests to address the following topics:

- 1. comparison of active and placebo after test article dose 1
  2. comparison of active and placebo after test article dose 2, with and without testing for the impact of, and adjustment for, outcomes after dose 1
  3. comparison of active and placebo in successive test article doses
  4. comparison of placebo in successive test article doses
  5. comparison of change from active to active with change from placebo to placebo

Items c. and d. are paired comparisons, which may be performed by McNemar tests or, more flexibly, by General Estimating Equation (GEE) models with a binary or continuous outcome. Items b. and e. are tests of interaction: dose 1 by dose 2 in item b., and period by vaccine in item e. and also accessible by GEE models.

### 9.2.3 Adverse Events:

Adverse events will be tabulated by body system using the COSTART dictionary, by severity, by seriousness, by relationship to study drug, and by elapsed time since last exposure to study drug. This yields primarily binary data (for each subject, the given COSTART event did or did not occur) which may be analyzed in the same manner as the binary reactogenicity events. Since multiple comparison corrections will not be used in this safety analysis, this analysis is vulnerable to grossly elevated type I error.

### 9.2.4 Vital Signs and Clinical Laboratory Measures:

Mean values for each measure will be plotted separately by treatment group, including ± one standard deviation. Extreme values and/or outliers will be discussed individually. For clinical laboratory measures within cross-over groups, quantitative outcomes at baseline and day 28 will be compared by paired Student’s t-tests and Wilcoxon signed rank tests. Binary outcomes will be compared by McNemar’s tests. This analysis is also vulnerable to elevated type I error.

## 9.3 Illness Definitions and Analysis for Challenge Component:

9.3.1 Fever:

Fever will be defined as any oral temperature ≥ 37.9°C which is confirmed by a repeat observation at an interval of not less than 20 minutes and not more than 60 minutes. Prior experience has shown that electronic digital thermometers used in climate-controlled environments may not precisely reflect the normal oral temperature range established in the era of mercury bulb instruments used in ambient conditions. Accordingly, an alternative definition of fever, i.e., ≥ 3 standard deviations above the mean of the third temperature observation on **Challenge** Phase Day 1, will be utilized if significantly lower than 37.9º C.

9.3.2 Upper Respiratory Illness:

A subject will be considered to have upper respiratory illness if he or she has any one of the following on two (2) consecutive days, at least one (1) day of which must feature grade 2 severity, or if any of the following attain grade 3 severity once:

- Self-reported symptoms:
  - Rhinorrhea (runny nose), or
  - Nasal congestion (stuffy nose), or
  - Sore throat.
- Physician findings:
  - Nasal discharge
  - Otitis
  - Sinus tenderness
  - Pharyngitis

9.3.3 Lower Respiratory Illness:

A subject will be considered to have upper respiratory illness if he or she has any one of the following on two (2) consecutive days, at least one (1) day of which must attain grade 2 severity, or if any of the following attain grade 3 severity once:

- Self-reported symptoms:
  - Cough, or
- Physician findings:
  - New wheezing, rhonchi, râles, or signs of consolidation

9.3.4 Systemic Illness:

A subject will be considered to have upper respiratory illness if he or she has any one of the following on two (2) consecutive days, at least one (1) day of which must attain grade 2 severity, or if any of the following attain grade 3 severity once:

- Headache
- Myalgia and/or athralgia

9.3.5 Illness (Any):

A subject will be considered to have illness if he/she fulfills the criteria for fever, or upper respiratory illness, or lower respiratory illness, or systemic illness or any combination thereof.

9.3.6 Infection:

A subject will be deemed to be infected if he/she has any one of:

- Influenza virus shedding in nasal wash at any level, or
- Detection of influenza virus in the throat swab by using **culture, antigen detection or** molecular biology techniques, or
- A ≥ 4-fold increase in serum A/Panama/2007/99 HAI antibody titer between day 39 (± 3) and the final follow-up visit, and absent clinical disease suggesting influenza between discharge from the isolation unit and the final follow-up visit.

9.3.7 Laboratory-confirmed Influenza Illness:

A subject will be deemed to have laboratory-confirmed influenza illness if he/she meets the definitions for BOTH of:

- Influenza infection, and
- Illness (Any).

9.3.8 Analysis:

9.3.8.1 Power Considerations:

The analysis will consider both binary outcomes (e.g., illness vs. no illness; virus shedding vs. no virus shedding, etc.) and continuous outcomes (e.g., treatment group mean temperatures, tissue counts, peak viral shedding and shedding AUC, etc.). The variability, and potential magnitudes of the differences in the latter are *a priori* unknown. However, the virus challenge inoculum will be selected to yield *approximately* 50 - 60%, based on past experience, of the binary outcome “any illness.” (Closer control of this rate is difficult to ensure.) Assuming that a minimal efficacy level of 70% (where efficacy = [rate _controls_ -rate_treated_ ] x 100 / rate _controls_) is of interest to justify further development of a given dose or formulation, it is possible to calculate that a group size of 28 will yield ≥ 0.80 power to detect a ≥ 70% reduction in the rate of the binary outcome “any illness.”

9.3.8.2 Analyses:

The analysis concerns between-group comparisons of the four cross-over groups regarding clinical and immunologic binary outcomes. Alternative definitions provide six binary clinical outcomes (fever, upper respiratory illness, lower respiratory illness, systemic illness, illness [any], and illness + infection) that can be compared between groups by chi-square tests. More flexible analyses use logistic regression (SAS PROC LOGISTIC) with the presence or absence of influenza, separately for each definition, as the outcome. Covariates are cross-over study group, age, gender, race, and, for some models, titers after vaccinations 1 and 2.

Additional secondary analyses will be based on tissue counts (and weights, if feasible) as a marker of illness severity, and on virus shedding assessed by two methods: peak and a time-weighted average calculated as a trapezoidal-rule AUC. For both peak and AUC, viral shedding is in units of log_10_ (titer). These continuous data will be compared between cross-over groups by Student’s t-tests and Wilcoxon tests and, in a more flexible analysis, by linear model adjusted for the demographic and clinical covariates cited above.

Finally, the three binary measures of influenza and the two viral load estimates (peak titer and AUC) will be assessed relative to the two immunologic measures, HAI reciprocal titer and nasal secretory IgA. For the binary measures, mean viral load (for each measure) is compared by t-tests between those with and without influenza (for each definition). For the two immunologic measures, the test of association with viral load will be based on Pearson correlations of log-titers.

# 10.0 References:

1. Betts RF, Treanor JJ. 2000. Approaches to improved influenza vaccination. Vaccine 18:1690-5.

2. Glezen WP, Taber LH, Frank A, *et al*. 1997. Influenza virus infection in infants. Pediatr Infect Dis J 16:1065-8.

3. Webster RG. 2000. Immunity to influenza in the elderly. Vaccine 18:1686-9.

4. Monto AS, Davenport FM, Napier JA, *et al*. 1969. Effect of vaccination of a school-age population upon the course of an A2-Hong Kong influenza epidemic. Bull World Health Org 41:537-42.

1. Demicheli V, Jefferson T, Rivetti D, *et al*. 2000. Prevention and early treatment of influenza in healthy adults. Vaccine 18:957-1030.

6. Clements ML, Betts RF, Tierney EL, *et al*. 1986. Serum and nasal wash antibodies associated with resistance to experimental challenge with influenza A wild-type virus. J Clin Microbiol 24:157-60.

7. Clements ML, Murphy BR. 1986. Development and persistence of local and systemic antibody response in adults given live attentuated or inactivated influenza A virus vaccine. J Clin Microbiol 23:66-72.

1. Nichol KL, Margolis KL, Wuorenma J, *et al*. 1994. The efficacy and cost-effectiveness of vaccination against influenza among elderly persons living in the community. N Eng J Med 331:778-84.
2. Gross PA, Hermogenes AW, Sacks HA, et al. 1995. The efficacy of influenza vaccine in elderly persons. Ann Intern Med 123:518-27.

10. Murasko DM, Goonewardene IM. 1990. T-cell function in aging: mechanisms of decline. Ann Rev Gerontol Geriatr 10:71-96.

11. Lamm ME, Robinson JK, Kaetzel CS. 1992. Transport of IgA immune complexes across epithelial membranes: new concepts in mucosal immunity. Adv Exp Med Biol 327:91-4.

12. Mazanec MB, Kaetzel CS, Lamm ME, *et al*. 1992. Intracellular neutralization of virus by immunoglobulin A antibodies. Proc Natl Acad Sci USA 89:6901-5.

13. Lamm ME. 1998. Current concepts in mucosal immunity. IV. How epithelial transport of IgA antibodies relates to host defense. Am J Physiol 274:614-7.

14. Boyce TG, Gruber WC, Coleman-Dockery SD, *et al*. 2000. Mucosal immune response to trivalent live attenuated intranasal influenza vaccine in children. Vaccine 18:82-8.

15. Belshe RB, Mendelman PM, Treanor J, *et al*. 1998. The efficacy of live attenuated, cold-adapted, trivalent intranasal influenza virus vaccine in children. New Eng J Med 338: 1405-12.

16. Treanor JJ, Kotloff K, Betts RF, *et al*. 2000. Evaluation of trivalent, live, cold-adapted (CAIV-T) and inactivated (TIV) influenza vaccines in prevention of virus infection and illness following challenge of adults with wild-type influenza A (H1N1), A (H3N2) and B viruses. Vaccine 18:899-906.

17. Nichol KL, Mendelman PM, Mallon KP *et al*. 1999. Effectiveness of live, attenuated intranasal influenza virus vaccine in healthy working adults: a randomized clinical trial. JAMA 282:137-44.

18. Powers DC, Fries LF, Murphy BR, *et al*. 1991. In elderly persons live attenuated influenza A virus vaccines do not offer an advantage over inactivated virus vaccine in inducing serum or secretory antibodies or local immunologic memory. J Clin Microbiol 29:498-505.

19. Beyer WEP, Palache AM, deJong JC, Osterhaus ADME. 2002. Cold-adapted live influenza vaccine versus inactivated vaccine: systemic vaccine reactions, local and systemic antibody response, and vaccine efficacy. A meta-analysis. Vaccine 20:1340-53.

20. Waldman RH, Bond JO, Levitt LP, *et al*. 1969. An evaluation of influenza immunization; influence of route of administration and vaccine strain. Bull World Health Org 41:543-8.

1. Waldman RH, Mann JJ, Small PA. 1970. Immunization against influenza. JAMA 207:520-4.
2. Waldman RH, Wood SH, Torres EJ, Small PA. 1970. Influenza antibody response following aerosol administration of inactivated virus. Am J Epidemiol 91:575-84.
3. Liem KS, Jacobs J, Marcus EA, van Strik R. 1973. The protective effect of intranasal immunization with inactivated influenza virus vaccine. Postgrad Med J 49:175-9.
4. Fukumi H. Experience of nasal application of inactivated influenza vaccine. Develop Biol Stand 33:155-61.
5. Oh Y, Ohta K, Kuno-Sakai H, *et al*. 1992. Local and systemic influenza haemagglutinin-specific antibody responses following aerosol and subcutaneous administration of inactivated split influenza vaccine. Vaccine 10:506-11.
6. Muszkat M, Friedman G, Schein MH, *et al*. 2000. Local SIgA response following administration of a novel intranasal inactivated influenza virus vaccine in community residing elderly. Vaccine 18:1696-9.
7. Muszkat M, Ben Yehuda A, Schein MH, *et al*. 2000. Local and systemic immune response in community-dwelling elderly after intranasal or intramuscular immunization with inactivated influenza vaccine. J Med Virol 61:100-6.
8. Kuno-Sakai H, Kimura M, Ohta K, *et al*. 1994. Developments in mucosal influenza virus vaccines. Vaccine 12 (14);1303-10.
9. Wilschut J, de Haan A, Geerligs HJ, *et al*. 1994. Liposomes as a mucosal adjuvant system: an intranasal liposomal influenza subunit vaccine and the role of IgA in nasal anti-influenza immunity. J Liposome Res 4:301-14.
10. Glück R, Mischler R, Durrer, et al. 2000. Safety and immunogenicity of intranasally administered inactivated trivalent virosome-formulated influenza vaccine containing Escherichia coli heat-labile toxin as a mucosal adjuvant. J Infect Dis 181:1129-32.
11. Bourguignon P, Bisteau M, Veenstra S, et al. 2001. Reactogenicity and passage into the brain of enterotoxins and CPG-oligonucleotides administered intranasally to mice. Abstracts of the Fourth Annual Conference on Vaccine Research, Arlington VA, 23 - 25 April, 2001; p. 53.
12. Lowell GH. 1997. Proteosomes for improved nasal, oral or injectable vaccines. *In:* Levine MM, Woodrow GC, Kaper JB, Cobon GS (ed.), New Generation Vaccines, 2^nd^ ed. Marcel Dekker, New York, p. 193-206.
13. ElGuink N, Kris RM, Goodman-Snitkoff G, *et al*. 1989. Intranasal immunization with proteoliposomes protects against influenza. Vaccine 7:147-151
14. Levi R, Aboud-Pirak E, LeClerc C, *et al*. 1995. Intranasal immunization of mice against influenza with synthetic peptides anchored to proteosomes. Vaccine 13:1353-9.
15. Fries LF, Montemarano AD, Mallett CP, et al. 2001. Safety and immunogenicity of a proteosome-*Shigella flexneri* 2a lipopolysaccharide vaccine administered intranasally to healthy adults. Infect Immun 69: 4545-53.
16. Plante M, Jones T, Allard F, Torossian K, *et al*. 2002. Nasal immunizations with subunit proteosome influenza vaccines induces serum HAI, mucosal IgA, and protection against influenza challenge. Vaccine 20:218-25.
17. Treanor J, Burt D, Lowell G, et al. 2001. Phase I evaluation of an intranasal proteosome-influenza vaccine in healthy adults. Abstracts of the Fourth Annual Conference on Vaccine Research, Arlington VA, 23 - 25 April, 2001; p. 55.
18. Fries L, Treanor J, Burt D, et al. 2001. Safety and Immunogenicity of one- and two-dose regimens of proteosome-monovalent influenza vaccine given intranasally to healthy young adults. IVth International Symposium on Viral Respiratory Infections, Willemstadt, Curaçao, 29 Nov – 02 Dec, 2001.
19. Halperin SA, McNeil S, Smith B, et al. 2002. Phase I safety and immunogenicity of FluINsure proteosome-trivalent influenza vaccine given intranasally to adults. 42^nd^ ICAAC, San Diego, CA, September 2002.
20. Fries LF. 2002. Proteosome™-based Intranasal Vaccines for Influenza.. Invited presentation to the Vth International Symposium on Respiratory Viral Infections, La Romana, Dominican Republic, Dec. 5 – 8, 2002.
21. Clements ML, Subbarao EK, Fries LF, et al. 1992. Use of single-gene reassortant viruses to study the role of avian influenza A virus genes in attenuation of wild type human influenza A virus for squirrel monkeys and adult human volunteers. J Clin Microbiol 30:655-62.
22. Fries LF, Dillon SB, Hildreth JEK, *et al*. 1993. Safety and immunogenicity of a recombinant protein influenza A vaccine in adult human volunteers, and protective efficacy against wild-type H1N1 virus challenge. J Infect Dis 167:593-601.
23. Clements ML, Betts RF, Tierney EL, Murphy BR. 1986. Resistance of adults to challenge with influenza A wild-type virus after receiving live or inactivated virus vaccine. J Clin Microbiol 23:73-6.
24. Treanor JJ, Kotloff K, Bets RF, et al. 2000. Evaluation of trivalent, live cold-adapted (CAIV-T) and inactivated (TIV) influenza vaccines in prevention of virus infection and illness following challenge of adults with wild-type influenza A (H1N1), A (H3N2), and B viruses. Vaccine 18:899-906.
25. Clark A, Potter CW, Jennings R, et al. 1983. A comparison of live and inactivated influenza A (H1N1) virus vaccines 1. Short term immunity. J Hyg Cambridge 90:351-9.
26. Hayden FG, Treanor JJ, Betts RF, et al. 1996. Safety and efficacy of the neuraminidase inhibitor GG167 in experimental human influenza. JAMA 275:295-9.
27. Calfee DP, Peng AW, Cass LM, et al. 1999. Safety and efficacy of intravenous zanamavir in preventing experimental human influenza A virus infection. Antimicrob Agents Chemother 43:1616-20.
28. Zeger SL, Liang K-Y. 1986. Longitudinal data analysis for discrete and continuous outcomes, Biometrics 42:121-30.

# Appendix A. Proteosome-Trivalent Influenza Vaccine Dose Preparation Worksheet

Date: ___________________ (dd/mm/yy)

Lot no. of proteosome-trivalent influenza vaccine: 1066

Mean Influenza hemagglutinin (HA) concentration (per strain) **____V___** μg / mL

(**V** concentration will be added by sponsor before study commencement)

Step 1. Estimate required volume of vaccine required at each dose level today:

| 15 μg dose: | ______________ | X | 1.0 mL | = | ________ mL (volume A) |
| --- | --- | --- | --- | --- | --- |
|  | No. of subjects to receive 15 μg dose |  |  |  |  |
|  |  |  |  |  |  |
| 30 μg dose: | ______________ | X | 1.0 mL | = | ________ mL (volume B) |
|  | No. of subjects to receive 30 μg dose |  |  |  |  |

Step 2. Calculate number of stock vaccine vials needed today:

| For 15 μg dose: | _____ mL | | X | ( **V** / 54 ) | = | _______mL (volume C) |
| --- | --- | --- | --- | --- | --- | --- |
|  | (volume A) | |  |  |  |  |
|  |  | |  |  |  |  |
| For 30 μg dose: | _____ mL | | X | ( **V** / 107 ) | = | _______mL (volume D) |
|  | (volume B) | |  |  |  |  |
|  |  | |  |  |  |  |
| Number of vials | = | ( vol. C + vol. D ) | | | ÷ | 0.9 |

Step 3. Remove requisite number of stock vaccine vials (PINK label) from refrigeration. Obtain sufficient vials of diluent/placebo (WHITE label). Diluent/placebo vials contain 10 mL of diluent; enough in one vial to yield ten (10) placebo doses or provide diluent for approximately 25 active doses. Obtain vaccine dilution vials.

Step 4. Prepare a nasal spray pump reservoir vial labeled with the ID no. of each subject to be dosed.

Step 5. Label vaccine dilution vials (vials and labels provided by sponsor) for the active and placebo test articles.

Initial this page: ___________

Vaccine Dose Preparation Worksheet, page 2.

Step 6. Swirl stock vaccine vial(s) before withdrawing vaccine to ensure complete mixing. Prepare the final vaccine dilution and placebo by adding the following volumes to the vaccine dilution vial(s), using the calculations performed on page 1 of this worksheet.

|  |  | Stock Vaccine |  | Placebo/Diluent |
| --- | --- | --- | --- | --- |
|  |  |  |  |  |
| 15 μg active |  | _____ mL | + | ______ mL |
|  |  | Volume C |  | (Volume A – Volume C) |
|  |  |  |  |  |
| 30 μg active |  | _____ mL | + | ______ mL |
|  |  | Volume D |  | (Volume B – Volume D) |
|  |  |  |  |  |
| Placebo |  | 0 | + | ______ mL |
|  |  |  |  | (number of placebo subjects X 1.0 mL) |

Swirl each dilution vial gently to ensure complete mixing.

Step 7. Referring to the treatment assignment list, determine the appropriate test article for each subject and place 0.9 mL of active vaccine dilution or placebo in the reservoir vial of the nasal spray device. Assemble the nasal spray pump onto the reservoir vial. Enter the required information for the subject in question into the Dose Preparation Log.

Step 8. Remove the nosepiece cover and then the safety collar from the nasal spray pump. Hold the assembled pump upright and fully depress the actuator three (3) times to prime the pump. Replace the safety collar, then the nosepiece cover. Deliver the pump to the clinic for test article administration.

Step 9. All stock vaccine and placebo vials and all vaccine dilution vials should be recapped and retained in a separate, secure location (which may be at room temperature) for return to the sponsor. NO VIAL THAT HAS BEEN PREVIOUSLY ENTERED SHOULD BE RE-USED.

Sign and date this form: ______________________________ _____________

Signature Date

# Appendix B. Table B.1

IDB-13005 Time and Events Schedule: Immunization Phase

| **Immunization Phase Day** | **-150 to**  **-7** | **-90 to - 5** | **0** | **1** | **2** | **3** | **4** | **5** | **6** | **7** | **14** | **15** | **16** | **17** | **18** | **19** | **20** | **21** | **28** |
| --- | --- | --- | --- | --- | --- | --- | --- | --- | --- | --- | --- | --- | --- | --- | --- | --- | --- | --- | --- |
| **Screening consent** | **x** |  |  |  |  |  |  |  |  |  |  |  |  |  |  |  |  |  |  |
| **Medical history** | **x** |  | **x** |  |  |  |  |  |  |  | **x** |  |  |  |  |  |  |  |  |
| **A/Panama HAI titer** | **x** |  |  |  |  |  |  |  |  |  |  |  |  |  |  |  |  |  |  |
| **Clinical Trial Consent** |  | **x** |  |  |  |  |  |  |  |  |  |  |  |  |  |  |  |  |  |
| **ECG** |  | **x** |  |  |  |  |  |  |  |  |  |  |  |  |  |  |  |  |  |
| **Clinical hematology/chem.** |  | **x** |  |  |  |  |  |  |  |  |  |  |  |  |  |  |  |  | **x** |
| **Urinalysis** |  | **x** |  |  |  |  |  |  |  |  |  |  |  |  |  |  |  |  | **x** |
| **HIV serology** |  | **x** |  |  |  |  |  |  |  |  |  |  |  |  |  |  |  |  |  |
| **Drugs of abuse screening** |  | **x** |  |  |  |  |  |  |  |  |  |  |  |  |  |  |  |  |  |
| **Nasal wash for spec. IgA** |  | **x** |  |  |  |  |  |  |  |  |  |  |  |  |  |  |  |  | **x** |
| **Serum for HAI antibody** |  |  | **x** |  |  |  |  |  |  |  |  |  |  |  |  |  |  |  | **x** |
| **Whole blood for cytokine studies** |  |  | **x** |  |  |  |  |  |  |  |  |  |  |  |  |  |  |  | **x** |
| **β-HCG (females)** |  |  | **x** |  |  |  |  |  |  |  | **x** |  |  |  |  |  |  |  |  |
| **Complete physical examination** |  | **x** |  |  |  |  |  |  |  |  |  |  |  |  |  |  |  |  |  |
| **Vital signs** |  | **x** | **x** |  |  |  |  |  |  | **x** | **x** |  |  |  |  |  |  | **x** |  |
| **Brief directed examination** |  |  | **x** | **c** | **c** | **c** | **c** | **c** | **c** | **x** | **x** | **c** | **c** | **c** | **c** | **c** | **c** | **x** |  |
| **Test article dose** |  |  | **X** |  |  |  |  |  |  |  | **X** |  |  |  |  |  |  |  |  |
| **Immed. complaint quest.** |  |  | **x** |  |  |  |  |  |  |  | **x** |  |  |  |  |  |  |  |  |
| **Memory Aid** |  |  | **x** | **x** | **x** | **x** | **x** | **x** | **x** |  | **x** | **x** | **x** | **x** | **x** | **x** | **x** |  |  |
| **AEs/Con. meds.** |  |  | **x** |  |  |  |  |  |  | **x** | **x** |  |  |  |  |  |  | **x** | **x** |

**x** = scheduled event; **c** = contingent event, performed only if required by reactogenicity symptoms

**Note: Study days following day 0 represent the nominal study schedule. Acceptable variability at each time point is indicated in protocol text.**

# Appendix B. Table B.2

IDB-13005 Time and Events Schedule: Challenge Phase

| ***Day Relative to First Treatment*** | ***39*** | ***40*** | ***41*** | ***42*** | ***43*** | ***44*** | ***45*** | ***46*** | ***47*** | ***48*** | ***49*** | ***50*** | ***51*** | ***60 - 70*** |
| --- | --- | --- | --- | --- | --- | --- | --- | --- | --- | --- | --- | --- | --- | --- |
| **Challenge Phase Day** | **0** | **1** | **2** | **3** | **4** | **5** | **6** | **7** | **8** | **9** | **10** | **11** | **12** | **20 - 30** |
| **Admit to quarantine** | **x** |  |  |  |  |  |  |  |  |  |  |  |  |  |
| **Interval medical history** | **x** |  |  |  |  |  |  |  |  |  |  |  |  |  |
| **ECG** | **x** |  |  |  |  |  |  |  | **x** | |  |  |  | **c** |
| **Nasal wash for spec. IgA** | **x** |  |  |  |  |  |  |  |  |  |  |  |  | **x** |
| **Serum for HAI antibody** | **x** |  |  |  |  |  |  |  |  |  |  |  |  | **x** |
| **Complete Physical exam (with vital signs)** | **x** |  |  |  |  |  |  |  | **x** | | **c** |  |  | **x** |
| **β-HCG (females)** |  |  | **x** |  |  |  |  |  |  |  |  |  |  |  |
| ***Challenge inoculum*** |  |  | ***X*** |  |  |  |  |  |  |  |  |  |  |  |
| **Temperature only, q.i.d.** |  | **x** | **x** | **x** | **x** | **x** | **x** | **x** | **x** | **x** |  |  |  | **x** |
| **Symptom Diary Card** | **x** | **x** | **x** | **x** | **x** | **x** | **x** | **x** | **x** | **x** |  |  |  |  |
| **24 hour tissue counts** |  | **x** | **x** | **x** | **x** | **x** | **x** | **x** | **x** | **x** |  |  |  |  |
| **Directed physical examination, BP and pulse** |  | **x** | **x** | **x** | **x** | **x** | **x** | **x** | **x** | **x** |  |  |  |  |
| **Throat swab for detection and / or quantitation of influenza virus** |  |  | **x** | **x** | **x** | **x** | **x** | **x** | **x** | **x** |  |  |  |  |
| **Nasal wash for detection and / or quantitation of influenza virus** |  |  |  | **x** | **x** | **x** | **x** | **x** | **x** | **x** |  |  |  |  |
| **Oseltamivir treatment** |  |  |  |  |  |  |  |  | **x** | **x** | **x** | **x** | **x** |  |
| **Discharge from isolation** |  |  |  |  |  |  |  |  |  | **x** | **c** |  |  |  |
| **AEs/Con. meds.** | **x** | **x** | **x** | **x** | **x** | **x** | **x** | **x** | **x** | **x** |  |  |  | **x** |

**x** = scheduled event; **c** = contingent event, performed only if required by illness or antecedent abnormal ECG

**Note: Study days represent the nominal study schedule. Acceptable variability at each time point is indicated in protocol text.**

# Appendix C. Brief Examination Worksheet (Post-Immunization)

Study IDB-13005 Subject ID:_____________ Date:________ Observer’s Initials ______________

Study Day: 0 7 ± 1 14 21±1

For each category of observation, place an “X” in the box most descriptive of the observations made.

|  | **Grade 0** | **Grade 1** | **Grade 2** | **Grade 3** |
| --- | --- | --- | --- | --- |
| **Nasal mucosal inflammation** | None | Erythema or edema | Erythema and edema | Ulceration |
| **Nasal discharge** | None | Clear, serous, scant | Purulent | Bloody ± purulent |
| **Pharyngeal inflammation** | None | Mild or patchy erythema | Severe erythema | Purulent exudate |
| **Sinusitis** | None | **NO GRADE 1** | Mild tenderness | Severe tenderness or overlying erythema |
| **Cervical/ post-auricular nodes** | None | Minimal enlargement, firm, nontender | Moderate enlargement, firm, slight tenderness | V. enlarged, soft/fluctuant, severe tenderness |
| Otic inflammation | None | Dull tympanic membrane | Injected tympanic membrane | Retracted or bulging tympanic membrane, fluid |

# Appendix D. Prototype Memory Aid Page

**SID #:** __________  **Initials: ___ ____ ____**

|  | Study Day X | | | | | | | |  | | | | | | | | | | | | | | | | |  |
| --- | --- | --- | --- | --- | --- | --- | --- | --- | --- | --- | --- | --- | --- | --- | --- | --- | --- | --- | --- | --- | --- | --- | --- | --- | --- | --- |
| Today’s date (day/month/year) |  | |  | | 200__ | | | |  | |  | | | |  | | | |  | | | | | | | |
| Evening oral temperature: | °C | | | | | | | |  | | | | | | | | | |  | | | | | | | |
|  | Grade (Mark the appropriate box) | | | | | | | |  | | | | | | | | | | | | | | | | |  |
|  | **0** | **1** | | **2** | | **3** | | |  | | | |  | | | |  | | | |  | | | | |  |
| Did you feel tired or have less energy than usual? |  |  | |  | |  | | |  | | | |  | | | |  | | | |  | | | | |  |
| Was your appetite poor? |  |  | |  | |  | | |  | | | |  | | | |  | | | |  | | | | |  |
| Did you have a headache? |  |  | |  | |  | | |  | | | |  | | | |  | | | |  | | | | |  |
| Did you have muscle or joint aches? |  |  | |  | |  | | |  | | | |  | | | |  | | | |  | | | | |  |
| Did you have a runny nose? |  |  | |  | |  | | |  | | | |  | | | |  | | | |  | | | | |  |
| Did you have a stuffy nose? |  |  | |  | |  | | |  | | | |  | | | |  | | | |  | | | | |  |
| Did your nose burn or itch? |  |  | |  | |  | | |  | | | | | | | | | | | | | | | | |  |
| Did you have bleeding from your nose? |  |  | |  | |  | | |  | | |  | |  | |  | |  | |  | | |  | |  | |
| Did you have red or puffy eyes? |  |  | |  | |  | | |  | | |  | |  | |  | |  | |  | | |  | |  | |
| Did you have any sneezing? |  |  | |  | |  | | |  | | |  | | | | | | | | | |  | | | | |
| Did you have a sore throat? |  |  | |  | |  | | |  | | |  | | | | | | | | | |  | | | | |
| Did you have a cough? |  |  | |  | |  | | |  | | |  | | | | | | | | | |  | | | | |
| Did you have shortness of breath or wheezing? |  |  | |  | |  | | |  | | | |  | | | |  | | | |  | | |  | | |
|  |  | | | | | | |  | |  | | |  | | | | | |  | | | | | | | |
| Did you take any medications on this day? | No | | | | | | Yes | | | | | | | | | | | |  | | | | | | | |
| *If yes,* list medication(s) below: | | | | | | | | | | | | | | | | | | |  | | | | | | | |
|  | | | | | | | | | | | | | | | | | | |  | | | | | | | |
|  | | | | | | | | | | | | | | | | | | |  | | | | | | | |
|  | | | | | | | | | | | | | | | | | | |  | | | | | | | |
|  | | | | | | | | | | | | | | | | | | |  | | | | | | | |
| Grade Definitions:  *Grade 0* = Not at all.  *Grade 1* = I noticed it, but it didn’t really interfere with any of my usual activities significantly.  *Grade 2* = I had it, and it was bad enough that I couldn’t do a significant part of my usual activities.  *Grade 3* = I had it, and it was bad enough that I was not able to do most of my usual activities, or I had to get prescription medicine from a doctor. | | | | | | | | | | | | | | | | | | |  | | | | | | | |

# Appendix E. Immediate Complaints Questionnaire

Please help us learn more about the vaccine in this study by telling us whether you had any of the following symptoms in the half hour immediately after your vaccine dose. Place an “X” in the column which describes the worst degree of each problem or symptom that you experienced. If you simply didn’t have a certain problem or symptom, put your “X” in the “NONE” column.

Your Subject ID Number:__________ Your initials: __________ Dose 1 2

|  | **NONE** | **MILD** | **MODERATE** | **SEVERE** |
| --- | --- | --- | --- | --- |
| You might say:  Symptoms: | “I didn’t have it at all.” | “I had it; but it wouldn’t stop me from doing anything.” | “I couldn’t have gone back to my usual activities if it lasted; but it went away.” | “It’s still bad enough that I can’t go back to my normal activities.” |
| Burning or stinging in the nose |  |  |  |  |
| Burning or stinging in the throat |  |  |  |  |
| Itching in the nose, throat, or eyes |  |  |  |  |
| Shortness of breath |  |  |  |  |
| Light-headedness or dizziness |  |  |  |  |
| A new or itchy skin rash you didn’t have before |  |  |  |  |
| Feverishness |  |  |  |  |

If you had another bothersome or worrisome symptom not listed here that started after getting the vaccine, *please tell the study doctor before you leave the clinic*.

Appendix F. Influenza Symptom Diary Card

If you have difficulty completing any part of this diary card, please ask a Retroscreen staff member for help.

Your Study ID Number: 40-_______ Your Initials: ___ ___ ___ Date: _____/ ____/ ____ (dd/mm/yy)

Challenge Phase Day (circle one): 0 1 2 3 4 5 6 7 8 9 Morning Evening ( tick one)

Place an “**X**” in the box in each symptom row that best describes how you have felt since completing your last diary card. Grade your symptoms based on the descriptions provided. Use the space to the right to note down any other symptoms you want to discuss with the doctor.

| **Grade:** | **0** | **1** | **2** | **3** | **Other Symptoms:** |
| --- | --- | --- | --- | --- | --- |
| **You might describe it as:** | I don’t have it. | It’s just noticeable. | It’s **bothersome** from time to time, but it doesn’t stop me from participating in activities | It’s quite bothersome most or all of the time, and it stops me from participating in activities |  |
| **SYMPTOMS:** |
| **Runny nose** |  |  |  |  |
| **Stuffy nose** |  |  |  |  |
| **Sore throat** |  |  |  |  |
| **Cough** |  |  |  |  |
| **Headache** |  |  |  |  |
| **Muscle and / or joint aches** |  |  |  |  |

# Reviewed by: ________________________________ Date: ____________ Time: (24 hour clock) __________

# Appendix G. Challenge Physical Examination Worksheet

Subject ID Number: 40-_______ Subject Initials: __________

Challenge Phase Day: 0 1 2 3 4 5 6 7 8 9 (circle one) ***OR*** if day not listed, (specify):______

Date: ___ (day) _____ (month) ______(year) Time: (24 hour clock): __________

BP: _____ / _____ mm Hg Pulse: _____ bpm Physician Initials: _________

|  | **GRADE 0** | **GRADE 1** | **GRADE 2** | **GRADE 3** |
| --- | --- | --- | --- | --- |
| **Upper Respiratory:** |  |  |  |  |
| **Nasal discharge** | None | Clear, serous; scant but slightly increased | Clear to white, obvious increased volume, ± minor blood streaks on tissue | Frankly purulent (yellow or green), obvious increased volume, or grossly bloody |
| **Otitis** | None | Dulled tympanic membrane | Inflamed, injected tympanic membrane | Retracted or bulging tympnainc membrane, obvious air-fluid level |
| **Pharyngitis** | None | Mild and / or patchy erythema | Marked and /or confluent erythema | Erythema and purulent exudate |
| **Sinus tenderness** | None | ***NO GRADE 1*** | Mild tenderness | Severe tenderness and / or overlying erythema |
| **Lower Respiratory:** |  |  |  |  |
| **New wheezes, râles, rhonchi, other** | None | ***NO GRADE 1*** | Scattered wheezes or rhonchi | Widespread wheezes or rhonchi; râles, dyspnea, or signs of consolidation |

# Comments: ___________________________________________________________________________
